# Muscle fibrosis and maladaptation occur progressively in CKD and are rescued by dialysis

**DOI:** 10.1172/jci.insight.150112

**Published:** 2021-12-22

**Authors:** Camille R. Brightwell, Ameya S. Kulkarni, William Paredes, Kehao Zhang, Jaclyn B. Perkins, Knubian J. Gatlin, Matthew Custodio, Hina Farooq, Bushra Zaidi, Rima Pai, Rupinder S. Buttar, Yan Tang, Michal L. Melamed, Thomas H. Hostetter, Jeffrey E. Pessin, Meredith Hawkins, Christopher S. Fry, Matthew K. Abramowitz

**Affiliations:** 1 Department of Athletic Training and Clinical Nutrition and; 2 Center for Muscle Biology, University of Kentucky, Lexington, Kentucky, USA.; 3 Department of Medicine and; 4 Institute for Aging Research, Albert Einstein College of Medicine, Bronx, New York, USA.; 5 Department of Nutrition and Metabolism, University of Texas Medical Branch, Galveston, Texas, USA.; 6 Department of Medicine, University of North Carolina School of Medicine, Chapel Hill, North Carolina, USA.; 7 Department of Molecular Pharmacology,; 8 Diabetes Research Center, and; 9 Fleischer Institute for Diabetes and Metabolism, Albert Einstein College of Medicine, Bronx, New York, USA.

**Keywords:** Muscle Biology, Nephrology, Chronic kidney disease, Extracellular matrix, Skeletal muscle

## Abstract

**BACKGROUND:**

Skeletal muscle maladaptation accompanies chronic kidney disease (CKD) and negatively affects physical function. Emphasis in CKD has historically been placed on muscle fiber–intrinsic deficits, such as altered protein metabolism and atrophy. However, targeted treatment of fiber-intrinsic dysfunction has produced limited improvement, whereas alterations within the fiber-extrinsic environment have scarcely been examined.

**METHODS:**

We investigated alterations to the skeletal muscle interstitial environment with deep cellular phenotyping of biopsies from patients with CKD and age-matched controls and performed transcriptome profiling to define the molecular underpinnings of CKD-associated muscle impairments. We examined changes in muscle maladaptation following initiation of dialysis therapy for kidney failure.

**RESULTS:**

Patients with CKD exhibited a progressive fibrotic muscle phenotype, which was associated with impaired regenerative capacity and lower vascular density. The severity of these deficits was strongly associated with the degree of kidney dysfunction. Consistent with these profound deficits, CKD was associated with broad alterations to the muscle transcriptome, including altered ECM organization, downregulated angiogenesis, and altered expression of pathways related to stem cell self-renewal. Remarkably, despite the seemingly advanced nature of this fibrotic transformation, dialysis treatment rescued these deficits, restoring a healthier muscle phenotype. Furthermore, after accounting for muscle atrophy, strength and endurance improved after dialysis initiation.

**CONCLUSION:**

These data identify a dialysis-responsive muscle fibrotic phenotype in CKD and suggest the early dialysis window presents a unique opportunity of improved muscle regenerative capacity during which targeted interventions may achieve maximal impact.

**TRIAL REGISTRATION:**

NCT01452412

**FUNDING:**

NIH, NIH Clinical and Translational Science Awards (CTSA), and Einstein-Mount Sinai Diabetes Research Center

## Graphical abstract

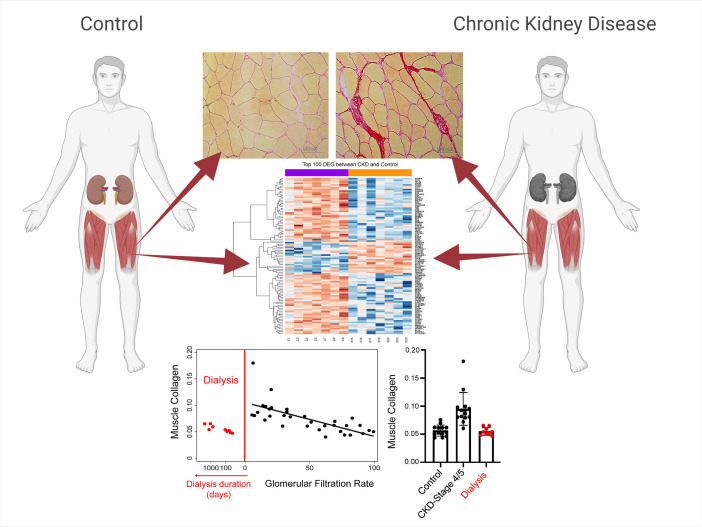

## Introduction

Chronic kidney disease (CKD) affects nearly 700 million people, approximately 1 in 11 individuals, worldwide (1). Patients with CKD are at markedly increased risk of disability, hospitalization, and death (2, 3); in 2017, 4.6% of deaths globally were attributable to CKD (1). Impaired physical function, common in CKD, is a major risk factor for these outcomes (3–5). CKD patients perform substantially below age-predicted norms on various clinically relevant physical performance tests, such as repeated chair stands, the timed-up-and-go test, and 6-minute walk distance (6–8). Previous studies have established muscle fiber–intrinsic deficits (e.g., alterations in protein metabolism, mitochondrial impairments) (9, 10), but to date interventions targeting these deficits have demonstrated marginal success at improving physical function (11–13). Greater interrogation of muscle fiber–extrinsic alterations in patients with CKD may offer novel therapeutic directions to preserve or enhance muscle function (14) critical to independent living.

Recent preclinical work underscores muscle-kidney crosstalk (15) and shared fibrogenic pathophysiology via dysregulation of the ECM in muscle and kidney tissue (16, 17). Our prior work was the first to translate these findings to humans, showing elevated collagen density in the quadriceps of patients with severely impaired kidney function (14). Importantly, we showed that fibrosis severity was associated with poorer leg extension strength and reduced endurance capacity, suggesting that muscle fibrosis in CKD patients is functionally significant (14). In skeletal muscle, intact ECM is required for optimal transmission of contractile force (18–21) and appropriate regulation of muscle regenerative mechanisms (22–24). ECM accumulation negatively affects muscle plasticity through alterations in muscle stem cell, also termed satellite cell, function. Thickened basal membrane due to collagen accumulation impedes satellite cell interaction with nearby muscle fibers (25), and increased stiffness impairs satellite cell proliferation, self-renewal, and activation (26, 27). Intriguingly, satellite cells and their activated daughter cells actively and concomitantly regulate the composition and remodeling of the ECM itself (18, 28), probably to promote the health of their niche to facilitate proper activation and renewal.

Critical to development of targeted interventions is whether muscle fibrosis in CKD is a complication observed only with severe disease or an insidious process progressing slowly beginning with early loss of kidney function, similar to well-defined sequelae of CKD, such as anemia and secondary hyperparathyroidism (29). Furthermore, the responsiveness of the fibrotic muscle phenotype to renal replacement therapy is unknown. We integrated transcriptomic profiling and immunohistochemical cellular phenotyping to determine alterations in skeletal muscle ECM in humans across a broad range of kidney function and in a subset of patients with end-stage renal disease (ESRD) following dialysis initiation. Using these procedures, we compared molecular and cellular alterations in muscle from patients with CKD and age-matched healthy controls. We tested the hypothesis that skeletal muscle fibrosis develops progressively as kidney function declines toward advanced CKD and that this associates with progressive loss of regenerative capacity and satellite cell homeostasis. Furthermore, we examined the role of dialysis therapy to restore a healthy muscle phenotype in patients with advanced CKD.

## Results

### Participant characteristics.

**Figure 1:**
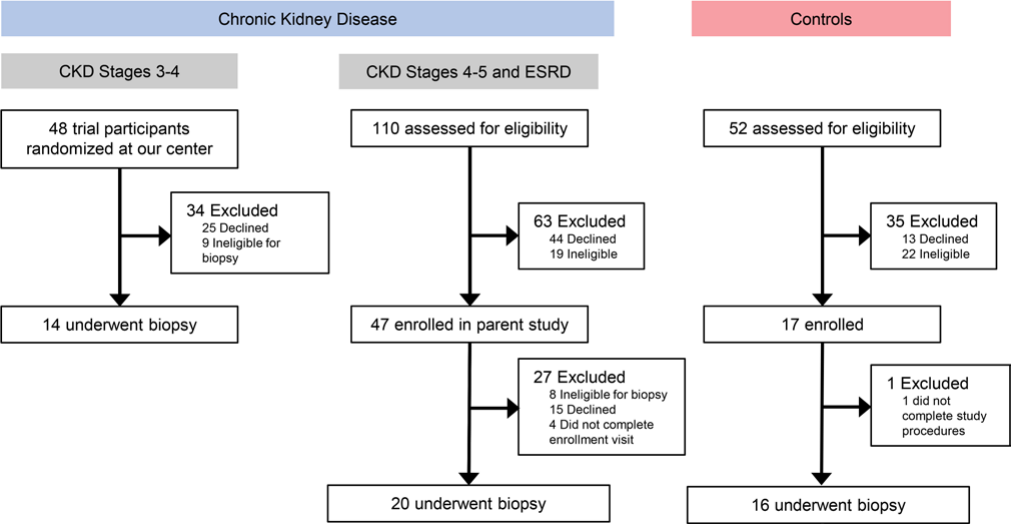
Flow diagram of study participation. ESRD indicates dialysis patients (transplant patients were not recruited).

Thirty-four patients and 16 controls participated in this study (Figure 1). At the first muscle biopsy, 29 patients with CKD were not receiving dialysis, and 5 had ESRD and were receiving dialysis therapy. Seven of the 29 CKD patients underwent a second muscle biopsy during follow-up; at the second biopsy, 3 patients had non-dialysis-dependent CKD, and 4 had reached ESRD and initiated dialysis. Age and sex were similar between the CKD and control groups (Table 1). Both groups were highly sedentary. Compared with controls, participants with CKD were more likely to have hypertension. Control participants lacked other comorbidities. Among the CKD patients, 55% had diabetes, 14% cardiovascular disease, and 7% peripheral vascular disease. The mean eGFR was 28.0 ± 15.5 mL/min/1.73 m^2^. As expected among the dialysis patients, hypertension was universal, and diabetes and cardiovascular disease were common (Table 1). Physical function testing and dietary assessments were performed in a subset of the cohort (Table 1). Performance on functional tests was similar between patients with CKD and controls, though numerically poorer among the CKD group. In total, scores on the SPPB ≤ 8 were present in 3 CKD patients, 2 controls, and 2 dialysis patients. DPI was similar between CKD patients and controls, whether assessed by food frequency questionnaire (FFQ) or 24-hour urine collection.

### Alterations in skeletal muscle collagen density and organization occur progressively through ESRD.

**Figure 2:**
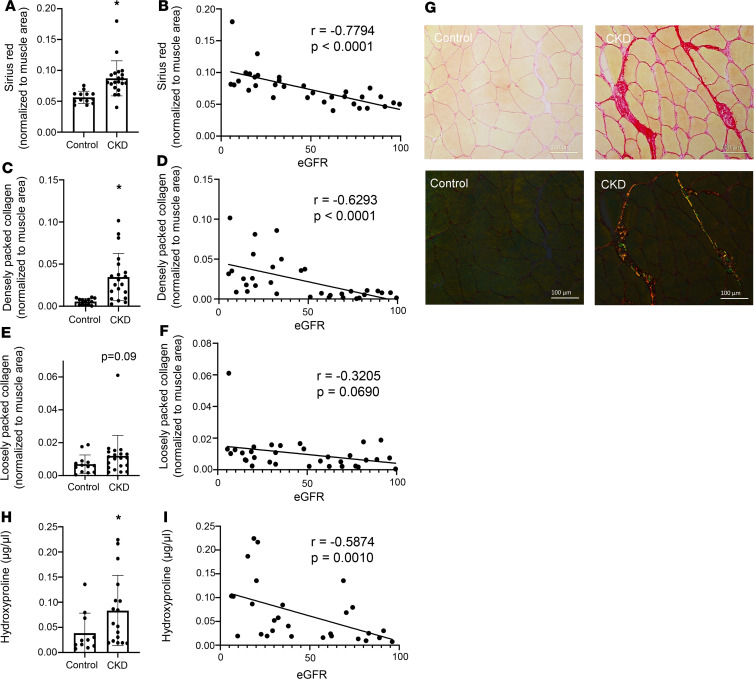
Collagen content and density are progressively elevated in skeletal muscle of patients with CKD. Proportion of ECM collagen content is elevated in CKD patients (**A**) and negatively associated with eGFR (**B**) (*n* = 33). Densely packed collagen content is elevated in CKD patients (**C**) and negatively associated with eGFR (**D**) (*n* = 33). Loosely packed collagen content was numerically higher but not significantly different in CKD patients (**E**) and not significantly correlated with eGFR (**F**) (*n* = 33). Representative images of picrosirius red staining in control and CKD muscle under both bright-field and polarized light (**G**). Total skeletal muscle collagen content assayed biochemically is elevated in CKD patients (**H**) and negatively associated with GFR (**I**) (*n* = 28). Comparisons made using 2-tailed *t* tests or Wilcoxon rank-sum tests. Spearman coefficients calculated to test correlations. Scale bar: 100 μm. *P < 0.05, control compared with CKD.

We collected vastus lateralis biopsies from participants with CKD and healthy age-matched controls that were assayed for collagen content via orthogonal histological and biochemical approaches. We showed striking fibrosis in patients with CKD histologically and biochemically. Collagen content in the ECM of vastus lateralis was significantly elevated in patients with CKD compared with healthy controls, as quantified by picrosirius red histological staining (Figure 2A; *P* = 0.006). A similar elevation was seen when comparing younger CKD patients with older controls (Supplemental Figure 1; supplemental material available online with this article; https://doi.org/10.1172/jci.insight.150112DS1). Strikingly, lower eGFR (i.e., more severe impairment in kidney function) was strongly associated with greater skeletal muscle ECM collagen content measured by picrosirius red histological staining, explaining 48% of the variance in muscle collagen (Figure 2B; *P* < 0.0001). Each 10 mL/min/1.73 m^2^ lower eGFR was associated with 0.6% (95% CI 0.4% to 0.9%, *P* < 0.001) higher muscle collagen content. This association was independent of age, sex, and race and diabetes, hypertension, and cardiovascular disease status (0.5% [95% CI 0.1% to 0.9%], *P* = 0.01) (see Supplemental Table 1 for unadjusted and adjusted associations of eGFR with histological parameters) and additional adjustment for fiber cross-sectional area (0.5% [95% CI 0.1 to 0.9%], *P* = 0.03). eGFR was independently associated with skeletal muscle collagen even when restricted to participants without a history of diabetes, cardiovascular disease, or peripheral vascular disease, in whom every 10 mL/min/1.73 m^2^ lower eGFR was associated with 0.4% (95% CI 0.1 to 0.7%, *P* = 0.01) higher muscle collagen content after adjustment for age, sex, race, and hypertension status. In the subset of participants with both accelerometer data and picrosirius red staining (*n* = 24), additional adjustment for sedentary time did not alter these results (multivariable model, 0.4%, 95% CI –0.02% to 0.9%, *P* = 0.061; additional adjustment for sedentary time, 0.5%, 95% CI –0.02% to 0.9%, *P* = 0.060).

Excessive collagen content (fibrosis) impedes muscle contractile function, and collagen alignment is associated with enhanced muscle passive stiffness (30). Densely packed collagen content was particularly elevated in skeletal muscle of CKD patients (Figure 2C; *P* < 0.001) and was strongly associated with eGFR (Figure 2D; *P* < 0.001). This association was also unchanged after multivariable adjustment (unadjusted, 0.3 log units [95% CI 0.2–0.4, *P* < 0.001], versus adjusted, 0.3 log units [95% CI 0.1–0.4, *P* = 0.01], per 10 mL/min/1.73 m^2^ lower eGFR). Loosely packed collagen was numerically higher in subjects with CKD, but neither this association nor its correlation with eGFR reached statistical significance (Figure 2, E and F; *P* = 0.09 and *P* = 0.069, respectively; see Figure 2G for representative images). The elevation in particular of densely packed collagen may impair muscle regenerative capacity, as elevated collagen density and low substrate elasticity suppress satellite cell activity and self-renewal (26). Skeletal muscle collagen measured biochemically by hydroxyproline content was also elevated in patients with CKD (Figure 2H; *P* = 0.026) and was associated with eGFR (Figure 2I; *P* = 0.0010), independently confirming our histological results. Furthermore, myofiber cross-sectional area (CSA) was not altered in patients with CKD (Supplemental Figure 2A) and was not associated with eGFR (Supplemental Figure 2B), suggesting that alterations to collagen content were not mediated by myofiber atrophy.

### Satellite cell abundance and activity are impaired in skeletal muscle of CKD patients.

**Figure 3:**
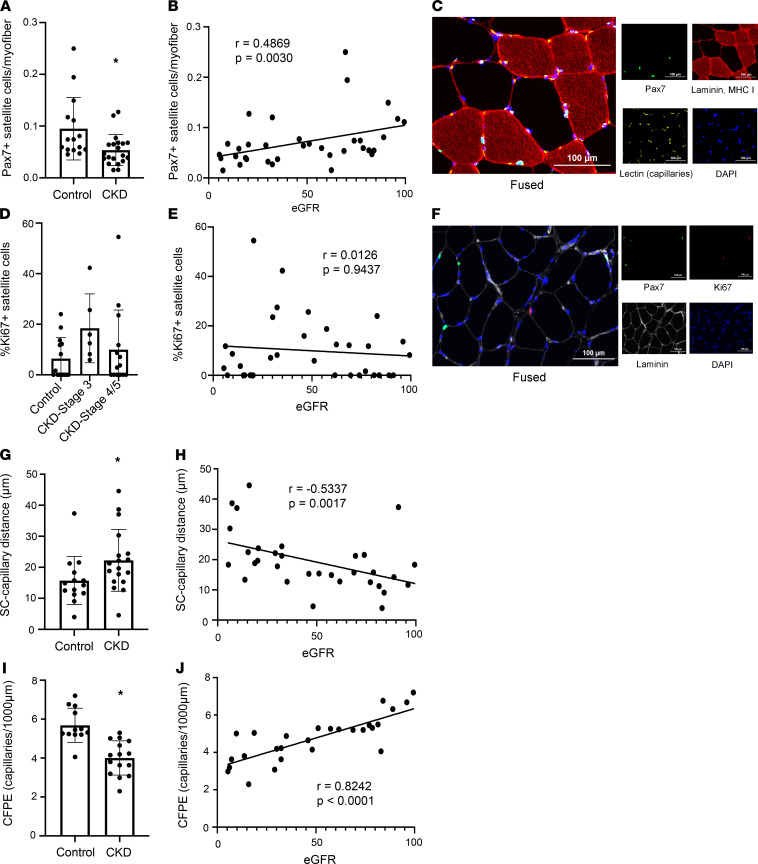
Satellite cell abundance, proximity of satellite cells to capillaries, and capillary density are lower in skeletal muscle of patients with CKD. Satellite cell abundance is lower in CKD patients (**A**) and positively associated with eGFR (**B**) (*n* = 35). Representative images of satellite cell and capillary staining (**C**). Satellite cell activation is numerically, but not statistically, elevated in patients with stage 3 CKD only (**D**) and not associated with eGFR (**E**) (*n* = 34). Representative images of activated satellite cell staining (**F**). The distance between satellite cells and nearest capillary is elevated in subjects with CKD (**G**) and negatively associated with eGFR (**H**) (*n* = 32). CFPE, an index of capillary density and blood-muscle exchange, is lower in subjects with CKD (**I**) and positively associated with eGFR (**J**) (*n* = 27). Comparisons made using 2-tailed *t* tests or Wilcoxon rank-sum tests. Spearman coefficients calculated to test correlations. Scale bar: 100 μm. *P < 0.05, control compared with CKD.

Satellite cells are the primary skeletal muscle stem cells that enter the cell cycle in the presence of an activating stimulus (i.e., injury, exercise) and differentiate to fusion-competent myoblasts to facilitate myonuclear accretion and promote regeneration and/or myofiber growth (31, 32). Satellite cells also communicate with fibrogenic cells to regulate ECM collagen deposition and subsequent fibrosis of skeletal muscle (18). Immunohistochemically, we determined that satellite cell abundance was significantly decreased in skeletal muscle of patients with CKD compared with healthy controls (Figure 3A; *P* = 0.04). Furthermore, there was a graded association of lower eGFR with lower satellite cell abundance (Figure 3B; *P* = 0.003) independent of age, sex, and race and diabetes, hypertension, and cardiovascular disease status (0.1 log units [95% CI 0.01–0.2] per 10 mL/min/1.73 m^2^ lower eGFR; *P* = 0.035; see Figure 3C for representative images). Consistent with these results, fiber type–specific satellite cell abundance was numerically lower in patients with CKD; similarly, modest correlations were observed with eGFR, though only statistically significant for type 1 satellite cell content (Supplemental Figure 3).

In healthy skeletal muscle, satellite cells remain reversibly quiescent until presented with an activating stimulus necessitating myofiber regeneration and/or growth. Using Ki67 as a marker of activation to indicate entrance to the cell cycle, we assessed satellite cell activation status. We observed a nonsignificant elevation in the mean frequency of Ki67^+^ satellite cells in CKD patients (control: 6.4% ± 2.2%, CKD: 12.5% ± 3.4%, *P* = 0.196). Separating CKD patients by disease stage, satellite cell activation was numerically elevated during stage 3 (Figure 3D) but not in stages 4/5. eGFR did not demonstrate a clear relationship with satellite cell activation status (Figure 3E; see Figure 3F for representative images). Myonuclear density and 2-dimensional myonuclear domain (μm^2^/myonucleus) were not altered in patients with CKD, and neither was significantly correlated with eGFR (Supplemental Figure 4).

### Capillary supply to satellite cells and myofibers is lower in skeletal muscle of CKD patients.

Capillaries are vital to supply oxygen and nutrients, and remove waste products, in skeletal muscle. Capillary density can be negatively affected during aging and physical inactivity (33), which may mitigate the responsiveness of skeletal muscle to anabolic stimuli like resistance exercise (34). Satellite cell–capillary crosstalk within skeletal muscle is integral to facilitate satellite cell activation and function (35) and support angiogenesis (35). We measured the distance between satellite cells and the nearest capillary and observed greater satellite cell–capillary distance in participants with CKD (Figure 3G; *P* = 0.048). Additionally, the proximity of satellite cells to capillaries was correlated with eGFR (Figure 3H; *P* = 0.0017), with lower eGFR independently associated with a greater distance between a satellite cell and the nearest capillary (*P* = 0.001 after multivariable adjustment). As with muscle collagen content, this association remained significant after exclusion of participants with a history of diabetes, cardiovascular disease, or peripheral vascular disease (*P* = 0.004).

We further interrogated capillary supply to myofibers via the capillary-to-fiber perimeter exchange index (CFPE), to assess the surface area of contact/exchange between capillaries and myofibers (36). CFPE was significantly reduced in participants with CKD (Figure 3I; *P* < 0.001), indicating impaired capillary supply to myofibers. eGFR was strongly associated with CFPE (Figure 3J; *P* < 0.0001; *P* = 0.0009 after multivariable adjustment; *P* = 0.0024 after exclusion of diabetes, cardiovascular disease, or peripheral vascular disease), demonstrating a similar relationship to that observed between eGFR and satellite cell–capillary distance. Thus, lower eGFR was associated with poorer capillary supply to myofibers and greater satellite cell–capillary distance, even among individuals without clinically apparent vascular disease.

### Skeletal muscle global transcriptomic differences reveal downregulation of angiogenic and fibrotic pathways in CKD.

**Figure 4:**
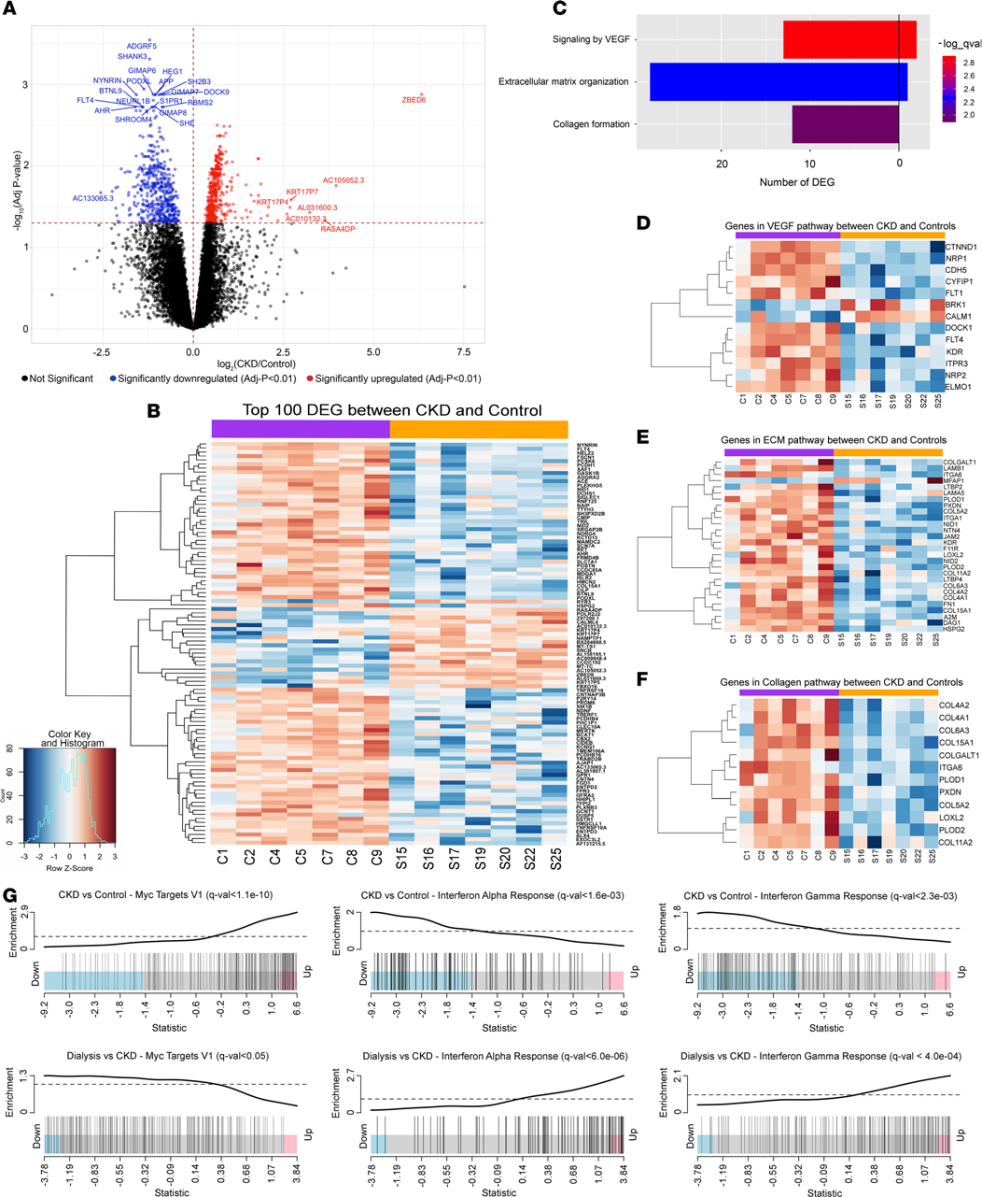
Transcriptomic differences in skeletal muscle of patients with CKD reveal alteration of genes within angiogenic and fibrotic pathways and rescue of Myc and interferon pathways postdialysis. Volcano plot highlighting global gene expression differences in CKD versus control (**A**). Heatmap with the top 100 differentially expressed genes in CKD (**B**). Orange bar, CKD; purple bar, control. Genes involved in VEGF signaling, ECM organization, and collagen formation are downregulated in CKD (**C**). Heatmaps of genes belonging to VEGF (**D**), ECM organization (**E**), and collagen formation (**F**) pathways that are differentially expressed in CKD versus control (*n* = 14). Orange bar, CKD; purple bar, control. GSEA plots reveal differential regulation of Myc, interferon-α, and interferon-γ pathways in CKD and rescue (change in regulation toward control values) after dialysis (**G**) (*n* = 3).

To investigate the transcriptomic differences in skeletal muscles from CKD patients, we profiled their global gene expression (*n* = 7 CKD, 7 control). Comparison of transcript abundances in the skeletal muscle between CKD patients and healthy individuals revealed 967 differentially expressed genes (*q* < 0.05) of the 14,995 genes tested (Figure 4, A and B, and Supplemental Table 2). Of these, 464 genes were upregulated while 503 genes were downregulated in CKD. Within the genes identified as differentially expressed, 10 protein-coding genes displayed an absolute log_2_ fold change > 2 (Supplemental Table 3). Principal component analysis demonstrated a clear shift in the transcriptomic profile of CKD patients compared with healthy individuals (Supplemental Figure 5). Pathway overrepresentation analyses identified 73 differentially regulated pathways, including several corresponding to ECM homeostasis, protein formation, mitochondrial function, DNA repair, Notch signaling, and cellular metabolism (Supplemental Table 4). We performed further analyses focusing on pathways closely related to our pathological findings. These identified that genes differentially expressed between CKD and controls primarily corresponded to downregulation of VEGF signaling, ECM organization, and collagen formation (Figure 4, C–F). Importantly, a number of the downregulated genes within the VEGF signaling pathway are integral to angiogenesis and tissue vascularization (*VEGFR1/FLT1*, *Cadherin 5/vascular endothelial cadherin*, *Neuropilin 1*) (see Supplemental Figures 6–9 for subsets of individual genes related to fibrosis, satellite cells/myogenesis, angiogenesis, and skeletal muscle atrophy). Gene set enrichment analysis (GSEA) on the global transcriptome characterized 26 pathways as significantly different between CKD patients and healthy individuals, of which 7 pathways were inferred to be upregulated and 19 pathways were identified as downregulated in CKD (*q* < 0.05) (Supplemental Table 5). Downregulated pathways in skeletal muscle of CKD patients included angiogenesis, Notch signaling, interferon-α response, interferon-γ response, and other inflammatory response pathways, while oxidative phosphorylation, Myc targets v1 and v2, reactive oxygen species, and fatty acid metabolism were upregulated (Figure 4G). Additionally, because CKD is associated with systemic inflammation, mitochondrial dysfunction, and oxidative stress, and pathway analyses identified related pathways as differentially regulated, we showed the impacts of CKD on transcription of individual genes related to inflammation (Supplemental Figure 10), DNA repair (Supplemental Figure 11), oxidative phosphorylation (Supplemental Figure 12), and reactive oxygen species (Supplemental Figure 13), which are consistent with overall downregulation of inflammatory response genes and upregulation of DNA repair, oxidative phosphorylation, and reactive oxygen species genes in muscle of CKD patients.

### Dialysis rescues pathological phenotype in skeletal muscle associated with CKD.

**Figure 5:**
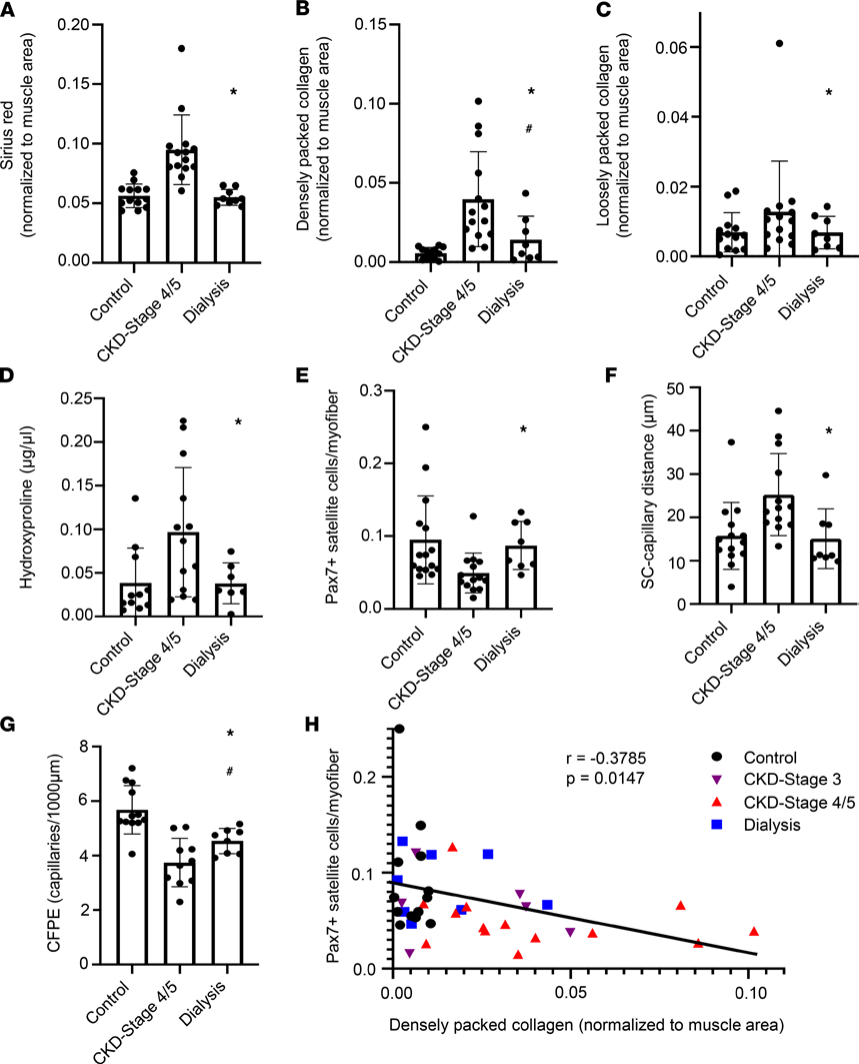
Dialysis rescues pathological phenotype in skeletal muscle associated with CKD. ECM collagen content (**A**) (*n* = 32), densely packed collagen (**B**) (*n* = 31), loosely packed collagen (**C**) (*n* = 31), and total muscle collagen assayed biochemically (**D**) (*n* = 29) are lower in subjects who have undergone dialysis compared with patients with advanced CKD. Total satellite cell abundance (**E**) (*n* = 33), the distance between satellite cells and their nearest capillary (**F**) (*n* = 31), and the CFPE ratio (**G**) (*n* = 27) are altered in patients who have undergone dialysis compared with those with advanced CKD, with a restoration toward control values. Data in **A**–**G** include only control, CKD stage 4/5, and dialysis patients. CKD stages 4/5 represent a subset of total CKD patients as presented in Figures 1 and 2 who have an eGFR < 30 mL/min/1.73 m^2^. Comparisons made using mixed effects models. Satellite cell abundance is negatively associated with densely packed collagen content (**H**) (*n* = 37). Spearman coefficient calculated to test correlation. *P < 0.05 compared with CKD stages 4/5. ^#^*P* < 0.05 compared with control.

To assess the impact of dialysis on the pathological muscle phenotype we observed in non-dialysis-dependent CKD patients, vastus lateralis biopsies were obtained from patients in our CKD cohort who reached ESRD and initiated dialysis and from additional ESRD patients undergoing dialysis. Of the 9 biopsies obtained from ESRD patients, 4 were from participants who had previously undergone biopsy as part of the CKD stage 4 and 5 cohort. Biopsies were obtained a median of 107 (range, 36–2600) days after the initiation of dialysis; 8 patients were receiving hemodialysis and 1 received peritoneal dialysis. Compared with patients with advanced (stage 4/5) non-dialysis-dependent CKD, total intramuscular collagen content was lower in dialysis patients (Figure 5A; *P* < 0.0001 after multivariable adjustment). This finding was independent of additional adjustment for fiber cross-sectional area (*P* < 0.0001) and decreased to levels similar to controls in all patients who had also undergone biopsy before dialysis initiation (Supplemental Figure 14). Further, densely and loosely packed collagen were similarly lower in dialysis patients (Figure 5B; adjusted *P* = 0.002 and Figure 5C; adjusted *P* = 0.112, respectively). Hydroxyproline content as a measure of muscle collagen content was also lower with dialysis (Figure 5D; adjusted *P* = 0.015). Furthermore, satellite cell abundance was restored with dialysis treatment (Figure 5E; adjusted *P* = 0.006), along with the proximity of satellite cells to capillaries (Figure 5F; adjusted *P* = 0.002) and capillary density measured as CFPE (Figure 5G; adjusted *P* = 0.001). Satellite cell function is regulated by the surrounding extracellular niche, with greater stiffness of the niche impairing satellite cell self-renewal (26). Strikingly, satellite cell abundance was inversely associated with densely packed collagen content (*P* = 0.015); this is shown in Figure 5H denoted by control, CKD stage 3, CKD stages 4/5, and dialysis patients, with control and dialysis values demonstrating a close grouping. Consistent with these results, we show alterations with dialysis to fiber type–specific satellite cell abundance and myonuclear density, with no differences observed in myofiber CSA or myonuclear domain (Supplemental Figure 15).

To explore whether muscle function might improve after dialysis initiation, we examined leg extension strength and endurance capacity in the 4 patients who underwent biopsy collection before and after dialysis initiation. Because of the multiple deleterious factors affecting muscle function in CKD (e.g., muscle atrophy, poor physical activity, mitochondrial dysfunction) and the potential for dialysis initiation to be associated with further deleterious effects (e.g., further decline in activity, accelerated muscle atrophy, hospitalization), we considered it unlikely to observe an overall improvement in function. Unsurprisingly, there was a general trend toward loss of strength and endurance over time beginning prior to dialysis initiation (Supplemental Figure 16). However, after adjustment for time and changes in weight and fiber CSA as measures of muscle atrophy, dialysis was associated with significantly increased leg extension strength measured in the right leg (8.1 kg, 95% CI 0.2–16.0, *P* = 0.043). Similar findings were observed with left leg extension strength (8.7 kg, 95% CI 0.8–16.6, *P* = 0.031). Endurance capacity, measured by the distance walked in 2 minutes, was also numerically higher after dialysis initiation when accounting for time, weight, and fiber CSA (25.3 m, 95% CI –2.4–52.9, *P* = 0.073). These data suggest that amelioration of the fibrogenic phenotype in CKD might be associated with an improvement in muscle function that is masked by the multitude of negative inputs affecting these patients.

Finally, we sought to identify a transcriptomic signature that could account for rescue of the pathological skeletal muscle phenotype in CKD. The dialysis samples (*n* = 4) included 3 paired CKD samples from the same patient obtained before starting dialysis and 1 unpaired sample after dialysis initiation. No genes were found to be differentially expressed (*q* < 0.05) after starting dialysis. However, GSEA identified 3 pathways in which the regulation after starting dialysis changed gene expression toward control levels, consistent with possible rescue of the pathway (Supplemental Table 6). Specifically, in skeletal muscle of dialysis patients, there was upregulation of the interferon-α response and interferon-γ response pathways, which were downregulated in CKD patients; in contrast, the gene set Myc targets v1 was upregulated in CKD but downregulated with dialysis (Figure 4G).

### Sequelae of CKD and associated medications do not explain increased muscle collagen or its regression after dialysis initiation.

We considered that muscle fibrosis could be due to complications of CKD, such as mineral bone disease, metabolic acidosis, and anemia, or that its severity or regression might be affected by medications commonly prescribed to CKD patients. We calculated a CKD severity score integrating the severity of perturbations in serum bicarbonate, potassium, calcium, phosphate, parathyroid hormone, and hemoglobin (see Supplemental Table 7 for laboratory data). There was a clear interrelationship between lower eGFR, greater muscle collagen, and higher CKD severity (Supplemental Figure 17). However, muscle collagen content was not significantly correlated with the CKD severity score (*r* = 0.43, *P* = 0.068) after excluding 1 highly influential observation representing a patient with the most extreme laboratory values for each parameter (except serum potassium) and the highest muscle collagen burden. When these parameters were examined individually, muscle collagen content was significantly correlated only with serum calcium (*r* = –0.54, *P* = 0.017) and hemoglobin (*r* = –0.64, *P* = 0.0001). However, these associations were not statistically significant after adjustment for eGFR (*P* = 0.63 for serum calcium, *P* = 0.54 for hemoglobin), suggesting that lower kidney function per se accounted for these associations.

The association of eGFR with muscle collagen content remained significant and quantitatively unchanged after additional adjustment in multivariable models for blockade of angiotensin signaling with ACE inhibitors or ARBs (0.5% [95% CI 0.2 to 0.9%] higher muscle collagen per 10 mL/min/1.73 m^2^ lower eGFR, *P* = 0.005). eGFR association with muscle collagen content remained when also adjusting for use of vitamin D or activated vitamin D compounds (0.5% [95% CI 0.1 to 0.9%], *P* = 0.023); the same was true for the decrement in muscle collagen content in dialysis patients (*P* < 0.0001 after adjustment for angiotensin blockade or vitamin D use, respectively). Furthermore, 2 patients who were taking ARBs at the time of their first biopsy had stopped these medications during the transition to starting dialysis, before their second biopsy demonstrating regression of fibrosis. We were unable to statistically examine the impact of erythropoietin-stimulating agents, which are prescribed nearly universally to patients beginning dialysis, as no patients received these agents prior to dialysis initiation. However, 1 patient who experienced a 33% reduction in muscle collagen content after initiating dialysis had not been treated with an erythropoietin-stimulating agent (ESA), indicating that ESA use is unlikely to account for the rescue of this muscle phenotype. Finally, because essential amino acid (EAA) supplementation attenuates muscle atrophy, increases satellite cell abundance, and improves function in older adults following total knee arthroplasty (38, 39), we investigated diet changes following the initiation of dialysis. Although we did not have information on specific amino acids, we did not find evidence of increased DPI after patients started dialysis to suggest increased EAA consumption (Supplemental Table 8).

## Discussion

Our findings demonstrate progressive alteration of the skeletal muscle ECM in association with increasing severity of CKD, manifesting as fibrosis, capillary rarefaction, and loss of muscle stem cells. Accumulating collagen was enriched with densely packed collagen, suggesting stiffer ECM (30, 39); furthermore, greater densely packed collagen was associated with a reduction in satellite cell abundance, consistent with increased stiffness and dysregulation of the ECM negatively regulating the satellite cell niche (26). This phenotype was associated with broad alteration of the skeletal muscle transcriptome, including downregulated angiogenesis, alteration of ECM organization, and altered regulation of pathways related to stem cell renewal. Importantly, our findings demonstrate that alterations in skeletal muscle architecture are not simply complications of kidney failure (40–43) but instead begin early in the course of CKD and are exacerbated with disease progression. Surprisingly, initiation of dialysis was associated with regression, and indeed near-normalization, of muscle collagen content, collagen density, and satellite cell abundance, suggesting improved regenerative capacity. Taken together, we provide evidence for a dysregulated myofiber-extrinsic environment that likely impairs plasticity and regenerative capacity (17) and contributes to decreased muscle performance (14, 44). These results identify a fibrotic phenotype in patients experiencing an increasingly common chronic disease, and we find its reversal upon initiation of extracorporeal therapy.

Previously, we reported skeletal muscle fibrosis in patients with severe CKD (14). Excessive collagen content was associated with reduced muscular strength and endurance capacity, underscoring the functional impact of muscle fibrosis in patients with CKD. The functional relevance of muscle fibrosis is further supported by the relative improvement in physical function, after accounting for muscle atrophy, that accompanied regression of fibrosis following dialysis initiation. Although optimal force transduction within muscle is reliant on the ECM, an overabundance of collagen together with abnormal collagen architecture in skeletal muscle results in a stiffer matrix in which transmission of contractile force may be impaired (19–21, 30). Progressive posttranscriptional modification of ECM components, namely collagens, promotes increased cross-linking of collagen fibers and results in a stiffer muscle, evidenced by a 4-fold elevation of Young’s modulus (39). Importantly, eGFR explained 48% of the variability in muscle collagen in our cohort, supporting a robust relationship between severity of kidney dysfunction and worsening fibrosis. Our transcriptome profiling showed downregulation of collagen formation pathways in CKD muscle despite the advanced nature of the fibrotic phenotype. Similar findings have been shown in severe fibrosis of the liver (cirrhosis) (45) and kidney (CKD) (46). In patients with CKD, we observed an overarching fibrotic muscle phenotype characterized by excessive accumulation of total and densely packed collagen that may be indicative of a stiffer matrix.

In addition to regulating force transmission and subsequent muscle function, skeletal muscle ECM is an integral controller of muscle regeneration through regulation of the satellite cell niche (47). Muscle damage and impaired repair mechanisms lead to fibrotic transformation of the ECM, which increases the susceptibility to injury and further impairs muscle repair (48). Specifically, satellite cell activity is suppressed when substrate elasticity is low (26), suggesting muscle stiffness via excessive collagen accumulation and cross-linking may impede satellite cell function and cause low satellite cell abundance, as we report in patients with CKD. Satellite cells are also reciprocal regulators of their own niche, regulating collagen biosynthesis by interstitial fibrogenic cells via extracellular vesicle paracrine action (28). In patients with CKD, we propose a fibrotic muscle phenotype impairs satellite cell function and abundance, thereby limiting muscle regenerative capacity. Whether satellite cells in this environment are compromised and less able to regulate their own niche is unknown but represents an alternative regulatory mechanism of CKD-associated muscle fibrosis.

Muscle fibrosis is speculated to result in reduced density and direct contact of capillaries with individual muscle fibers (49, 50). Work in other fields such as cancer shows an inverse relationship between ECM density and perfusion of the tumor (51). Recent evidence has underscored synergism between hypoxia and initiation of a fibrotic phenotype in skeletal muscle; in vitro evidence supports hypoxia-induced fibrogenic signaling within myofibers themselves (50). Intramuscular hypoxia occurs also in CKD: complementary methods have demonstrated reduced muscle oxygen uptake and oxygen supply dependence (52, 53), indicative of a hypoxic environment. This is mediated at least partially by progressive capillary rarefaction (52); the extent of capillary dropout increases with loss of kidney function (52, 54, 55) and is independent of changes in blood pressure (56). Capillary dropout in the setting of severe CKD has been observed even in children (57). We report markedly lower muscle capillarization in patients with CKD and a direct association with eGFR across a wide range of kidney function. Additionally, we report angiogenic signaling was downregulated. Similar downregulation of hypoxia- and angiogenesis-related pathways is found in CKD animal models (52, 54, 55), and rescue of HIF-1α activates proangiogenic VEGF target genes and prevents the loss of capillary density (55). In sum, these results suggest that impaired angiogenesis drives the loss of skeletal muscle capillary networks in CKD.

Further, microvascular rarefaction and satellite cell depletion likely have synergistic effects on the development of muscle fibrosis in CKD. Loss of capillary networks and ensuing hypoxia would be expected to activate signaling via the HIF/Notch axis (58), which promotes satellite cell self-renewal and enhances muscle regeneration (58, 59). However, in patients with CKD, Notch signaling was downregulated; absence of this renewal stimulus could contribute to exhaustion of satellite cell pools. Moreover, the physical distance between satellite cells and endothelial cells was significantly elevated in the muscle from patients with CKD, further impacting muscle reparative processes (60). The proximity of satellite cells and capillaries is associated with satellite cell self-renewal, as endothelial cells deliver the Notch ligand DII4, maintaining appropriate quiescence of satellite cells (61). An increased distance between each satellite cell and the nearest capillary likely limits delivery of DII4, impairing maintenance of satellite cell quiescence in the absence of a regenerative stimulus and ultimately resulting in the reduction in satellite cell abundance that we observe with CKD progression. Thus, our data indicate a failure of counterregulatory, homeostatic responses in patients with CKD, likely facilitating further fibrotic transformation and impairment of the regenerative capacity of skeletal muscle.

Remarkably, despite the seemingly advanced nature of this fibrogenic phenotype, it was responsive to dialysis initiation. Regression of organ fibrosis in humans has been reported only rarely (62). However, we documented reductions in muscle collagen deposition using orthogonal methods and found concomitant decrements in collagen density; satellite cell abundance and capillarization also increased toward control levels. Such a salutary response to dialysis initiation, although unexpected, is supported by prior work in aging. Aged skeletal muscle, like that in CKD, is characterized by diminished regenerative capacity and loss of Notch signaling (63); aging-induced impairment of muscle progenitor cell function is restored by exposure to the circulation of young mice, preventing fibrosis (59, 64). Thus, the effects of dialysis on the muscle interstitium could be due to removal of a circulating factor. Although dialysis does not restore a “youthful” milieu, it clears from the systemic circulation a number of potentially toxic solutes that accumulate due to kidney failure (65), likely beginning early in CKD (66, 67). For example, certain uremic toxins — which accumulate systemically with decreased GFR — impair myogenic progenitor proliferation and differentiation (68), blunting muscle regenerative capacity. Removal of these and other toxins by dialysis treatment may mediate a restoration of satellite cell activity and result in the rescued satellite cell abundance we observed in subjects with CKD upon initiation of dialysis, supporting restoration of muscle regeneration and a healthier skeletal muscle phenotype.

In addition, our data provide further support for the notion of CKD as a state of accelerated aging (69). Myc signaling was upregulated in CKD but downregulated in dialysis patients toward control expression levels; whereas long-term overexpression of Myc signaling depletes stem cell populations (70), reduction in Myc activity extends health span and reduces organ fibrosis (71). Our data also parallel those from aging studies in documenting a decoupling of muscle-specific inflammation from systemic inflammation. We previously found lower inflammatory marker gene expression and fewer proinflammatory, or M1 type, macrophages in the skeletal muscle of CKD patients compared with age-matched controls, despite greater systemic inflammation in CKD (14); our transcriptome profiling confirms a broad downregulation of inflammatory pathways in CKD muscle. Similarly, aged skeletal muscle is characterized by a relative paucity of M1 type macrophages (72), which fail to respond appropriately to anabolic stimuli (73). In addition, compared with young mice, the skeletal muscle of aged mice exhibits downregulation of interferon response genes following injury (74). Absence of interferon-γ signaling following muscle injury impairs regeneration (75), whereas restoration of interferon activity enhances satellite cell activation, promotes muscle regeneration, and inhibits a fibrotic response (74, 76). Thus, it is notable that interferon signaling was upregulated in dialysis patients. Over the past 2 decades, numerous studies have linked ESRD and the dialysis procedure itself with inflammation (77, 78); and in particular, the T cell secretome of patients receiving hemodialysis is characterized by an elevated interferon-γ profile (79). Last, our transcriptomic data are consistent with prior work showing broad metabolic dysfunction in CKD, including effects of oxidative stress and altered muscle bioenergetics (9, 80–82); these abnormalities are also implicated in aging-induced muscle dysfunction (83, 84). Whether these also contribute to muscle fibrosis and impaired regeneration in CKD requires further study; however, because metabolic dysfunction is well documented in ESRD patients (85–87), the regression of fibrosis in dialysis patients likely requires alternative mechanistic pathways. Taken together, these data suggest that dialysis may reverse the CKD muscle fibrogenic phenotype by partially counteracting mechanisms common to aging and CKD.

The reversal of this chronic fibrotic process was unexpected, especially given the poor physical performance often reported in chronic dialysis patients (88, 89). Our work indicates that uremic myopathy is a complex process with distinct components not affected uniformly by dialysis therapy. For example, mitochondrial dysfunction is associated with CKD severity but not ameliorated in patients receiving dialysis (9, 86). Thus, improvements in one aspect of muscle quality may be masked by other deficits. Remarkably, alterations of the ECM were uncoupled from muscle fiber atrophy following the initiation of dialysis, suggesting distinct regulatory programs driving these processes. The disconnect between atrophy and fibrosis in the current study is unique; while the molecular processes governing fibrogenesis and atrophy are fairly distinct, their pathophysiology is often entangled in chronic conditions like aging (90) and muscular dystrophy (91). Another consideration is that most studies of physical function in ESRD have failed to capture changes that occur during the transition period of dialysis initiation. Two recent studies that, like ours, investigated this transition period point toward improvements in muscle mass and physical function in some patients (92, 93). In addition, a recent study of ESRD patients demonstrated intact satellite cell function in response to high-intensity exercise (94), consistent with the restoration of satellite cell abundance and the muscle interstitial niche reported here. Revitalization of the satellite cell pool and restoration of vascular density in the transition period may serve as critical cellular effectors to enhance responsiveness to exercise interventions, prior to the adverse sequelae that accrue after years of dialysis treatment. Therefore, based on the improvement in muscle regenerative potential, the period following dialysis initiation may be an optimal time for exercise and nutritional interventions to enhance muscle function.

Finally, a major unmet need in nephrology is the development of biological indicators to optimize the timing of dialysis initiation. As the eGFR has proved unsatisfactory (95), current practice guidelines suggest basing the decision to start dialysis on signs and symptoms of kidney failure (96, 97), which puts patients at risk for malnutrition, sarcopenia, and frailty. Therefore, identification of a dialysis-responsive, functionally relevant complication of CKD has important implications. Although dialysis initiation is complicated by potential harms, the risk-benefit ratio may soon shift because of technological innovations, including the introduction of wearable technologies (98, 99). In the setting of a lower risk landscape, a novel biological indicator to guide the timing of dialysis initiation would maximize patient benefit. Additional investigation is warranted to determine if skeletal muscle fibrosis is such an indicator.

Strengths of the current investigation include transcriptome profiling, complementary measures of skeletal muscle collagen abundance, evaluation of both a dialysis and nondialysis CKD population, and demonstration of associations between kidney function and primary outcomes independent of age, sex, diabetes and cardiovascular disease status, and medication use. However, our study has limitations, which should be noted. One is the relatively limited number of CKD patients who have both pre- and postdialysis biopsies. Notably, few studies have followed patients through the transition period of dialysis initiation, and doing so while employing invasive procedures such as muscle biopsies presents substantial challenges. To our knowledge, this study is one of the first to do so using detailed clinical, histological, and molecular phenotyping. Future longitudinal studies will yield additional information on changes in muscle collagen and physical function in both the presence and absence of dialysis initiation. Transcriptomic profiling was performed in patients with advanced CKD; future work should examine alterations in relevant pathways in early CKD. Finally, another limitation of our study is the sole use of eGFR as an index of kidney function. eGFR measures one aspect of kidney function — filtration by the glomerulus — but does not account for secretion of organic solutes that accumulate in CKD patients and are likely toxic (100–102).

In conclusion, we demonstrate a progressive fibrotic muscle phenotype in patients with CKD that is associated with reduced regenerative capacity and is restored with dialysis treatment. Impairments within the interstitial muscle cellular environment may underlie poor physical performance (14, 44), leading to loss of muscle strength, reduced exercise capacity, and the development of mobility impairment, disability, and frailty (103–107). Having identified skeletal muscle fibrosis as a sequela of progressive CKD, these results provide the foundation for future research to further define cellular and molecular mechanisms and to determine its utility in CKD care.

## Methods

### Study population

The population of this study is drawn from 2 previously described studies (see Supplemental Methods for details) (14, 108). Participants with CKD stages 4 and 5 and ESRD and healthy sedentary controls were recruited between March 2015 and January 2020 from a prospective cohort study of patients with an eGFR < 30 mL/min/1.73 m^2^ (14). Healthy sedentary individuals without evidence of kidney disease (eGFR > 60 mL/min/1.73 m^2^ and urine albumin/creatinine ratio < 30 mg/g) were recruited as control participants. Dietary protein and energy intake were assessed using the Dialysis FFQ (109), which is a modified version of the Block FFQ (110). FFQs were analyzed by NutriQuest using the Minnesota Nutrition Data System for Research (111). In addition, 24-hour UUN was used to calculate DPI among CKD patients and controls as follows: DPI = 6.25 × (UUN in g/d + [weight in kg × 0.031]) (112). Unilateral knee extensor strength was measured using isometric dynamometry with a handheld dynamometer (Manual Muscle Test System, Lafayette Instrument). To ensure assessment of maximum strength, subjects were instructed to perform a maximal exertion contraction, and 2 trials were recorded. The highest result achieved in each leg was used for analysis. Endurance capacity was measured by the 2-minute walk test (113): participants were asked to walk back and forth over a 50-foot course as far as possible over 2 minutes. The distance covered is highly correlated with 6-minute walk distance (114). The SPPB is an established measure of mobility in older adults (115). It includes a 4-meter walk test, a standing balance test, and a 5-repetition sit-to-stand test and is scored 0–12, with higher scores indicating better function. Handgrip strength was measured twice in each hand using a handheld dynamometer (North Coast Medical). The maximum value attained using the dominant hand was used for analysis. Physical activity level was measured using triaxial accelerometers (Actigraph GT3X-BT) worn around the waist for 7 consecutive days. Wear time validation was performed as previously described (116). Intensity levels were defined based on counts per minute (117, 118). Sedentary time was classified according to daily time spent in sedentary bouts of 10 or more consecutive minutes, excluding sleep time (116). We previously reported ECM collagen content by picrosirius red staining in a subset of these participants (10 CKD patients and 10 control participants). Additional participants with CKD stages 3 and 4 were enrolled in a multicenter, double-blind, randomized, placebo-controlled trial of sodium bicarbonate therapy (ClinicalTrials.gov identifier NCT01452412) and underwent muscle biopsy between September 2011 and May 2015 (108). Only participants enrolled at the Albert Einstein College of Medicine were eligible for the biopsy component of the study. Data reported here, including muscle biopsy results, were collected at baseline before initiating study treatment. Lower extremity performance was assessed using a 10-repetition sit-to-stand test; the split time required to complete 5 repetitions was also recorded. Handgrip strength was measured as described above. All laboratory tests were conducted in the clinical laboratory of Montefiore Medical Center. eGFR was calculated by the Chronic Kidney Disease Epidemiology Collaboration equation (119). Study data were collected and managed using Research Electronic Data Capture tools hosted at the Albert Einstein College of Medicine (120).

### Muscle biopsies and tissue processing

Biopsy procedures have been previously described (14). Participants were admitted to the Clinical Research Center at 8 am following an overnight fast. Through an incision site 15 cm proximal to the superior border of the patella, approximately 100–150 mg of muscle tissue was collected from vastus lateralis using a 12-gauge biopsy needle (Bard Monopty, Bard Biopsy Systems). Additional details are available in the Supplemental Methods. Sample size for outcomes listed below was dependent on sufficient tissue quantity; certain participant biopsy samples provided insufficient muscle tissue for all downstream assays.

### Immunohistochemistry

Seven-micrometer-thick sections were cut with a cryostat at –25°C (HM525-NX, Thermo Fisher Scientific) and air-dried on slides for 1 hour. Slides were then stored at –20°C until immunohistochemical/histochemical staining was performed. Immunohistochemical techniques were performed as previously described by our group (121, 122). Details for individual assays are available in the Supplemental Methods.

### Image acquisition and analysis

Images were captured at ×100–×400 original magnification at room temperature using a Zeiss upright microscope (AxioImager M1). Image analysis was performed in a blinded manner using ImageJ Fiji or Zen software (v3.1, Zeiss). Picrosirius red staining was quantified to measure collagen content of the ECM using ImageJ Fiji software as previously described (121). The area of picrosirius red^+^ collagen was normalized to the total muscle area (mm^2^). Picrosirius red was also imaged under polarized light to quantify densely packed (red) and loosely packed (green) collagen relative to total muscle area (123, 124).

Satellite cell abundance was determined by costaining of Pax7 and DAPI within the laminin border. Pax7^+^DAPI^+^ cells within the laminin border were counted as satellite cells and normalized to total number of myofibers. Proliferation of satellite cells was assessed by costaining of Ki67 and Pax7. Ki67^+^Pax7^+^DAPI^+^ cells inside the laminin border were counted as proliferating satellite cells and normalized to total satellite cell number. Capillaries were measured as *Ulex Europaeus* agglutinin-positive cellular structures outside the myofiber laminin border, as previously described (125). CFPE was used to assess capillary density relative to myofiber perimeter and quantified as the ratio between the number of capillaries of each myofiber with a correction for capillary sharing and myofiber perimeter, as previously described (34). CFPE was calculated using at least 50 myofibers for each sample per published recommendations (126). Myovision software generated automated analysis of myofiber CSA, myonuclear density (total number of myonuclei normalized to total number of myofibers), and myonuclear domain (area of each individual myofiber normalized to number of myonuclei within that same myofiber) using laminin and DAPI (127).

### Hydroxyproline biochemical assay

To assess total muscle collagen content, hydroxyproline was assayed from approximately 10 mg of muscle tissue similar to our prior methods (128) using a modified protocol with a commercially available Hydroxyproline Assay Kit (MAK008, MilliporeSigma). Details are available in the Supplemental Methods.

### RNA-Seq

#### RNA isolation, sequencing, preprocessing, and alignment.

Total RNA was isolated, then sequenced on Illumina HiSeq 4000 system at Novogene Corporation, using a paired-end 150 bp dual-indexing protocol. Details are available in the Supplemental Methods (129–134). The raw sequences and quantified data are available in the Gene Expression Omnibus database under the accession code GSE157712.

#### Statistical modeling, differential gene expression, and GSEA.

All statistical tests for RNA-Seq analysis were carried out using the R statistical software R 4.0.2.

The raw counts were filtered using a counts-per-million cutoff of 0.5 (10/minimum library size in millions), in at least 7 samples (number of samples in the smallest group of comparison). The raw counts were normalized using the trimmed mean of M-values normalization in limma (v3.42.2). The voom function was applied to the normalized data to minimize heteroskedasticity and include precision weights for the mean-variance relationship for all genes (135). A principal component analysis was run to visually inspect any evident batch effects within the data, following which a linear model was fit on the voom-normalized data, using sample ID as the blocking variable to account for patient-specific variability between the paired CKD and dialysis samples. Differential gene expression was calculated for the following contrasts using the empirical Bayes statistic in limma: CKD patients versus healthy controls; CKD patients pre- versus postdialysis. Raw *P* values were adjusted for multiple testing using Benjamini-Hochberg correction, and a threshold of *q* < 0.05 was used to categorize the genes as differentially expressed. Pathway overrepresentation within the differentially expressed genes was carried out using ConsensusPathDB (136). GSEA was carried out on the voom-normalized gene expression data using the CAMERA algorithm within limma (137). The Hallmark gene sets for humans curated by the Walter and Eliza Hall Institute were used for GSEA and downloaded from http://bioinf.wehi.edu.au/software/MSigDB/human_H_v5p2.rdata

### Statistics

Histochemistry, immunohistochemistry, and hydroxyproline data were compared between CKD patients and controls using 2-tailed *t* tests or Wilcoxon rank-sum tests. Spearman correlation coefficients and linear regression models were used to test associations of eGFR with muscle outcome measures. Analyses including data collected from ESRD patients receiving dialysis were performed using mixed effects models including random intercepts to account for repeated measures in the subset who had undergone serial biopsies before and after the initiation of dialysis or who had second biopsies while not dialysis dependent. Patients noted as CKD stages 4/5 in Figure 4 represent a subset of all CKD patients with an eGFR < 30 mL/min/1.73 m^2^. Satellite cell abundance, satellite cell–capillary distance, densely packed collagen content, and loosely packed collagen content were log-transformed to satisfy model assumptions. Multivariable linear regression and mixed effects models were adjusted for age, sex, race, and history of diabetes, hypertension, and cardiovascular disease unless otherwise noted. To calculate the CKD severity score, we computed the sum of standardized differences from the mean for serum bicarbonate, potassium, calcium, phosphate, parathyroid hormone, and hemoglobin. For serum bicarbonate, serum calcium, and hemoglobin, we input the negative of the standardized value into the summative score, as progression of non-dialysis-dependent CKD induces a decrease in each parameter. All analyses were performed with Stata 13.1 (StataCorp). *P* < 0.05 was considered statistically significant.

### Study approval

The study protocols were approved by the Institutional Review Board of the Albert Einstein College of Medicine. Before inclusion in the study, written informed consent was provided by all participants.

## Author contributions

MH, JEP, CSF, and MKA designed and implemented the study. CRB, ASK, WP, KZ, JBP, KJG, MC, HF, BZ, RP, RSB, YT, MLM, and THH conducted experiments and acquired data. CRB, ASK, WP, KZ, JBP, KJG, CSF, and MKA analyzed data. CRB, ASK, CSF, and MKA made figures and drafted the manuscript. CRB, ASK, WP, KZ, JBP, KJG, MC, HF, BZ, RP, RSB, YT, MLM, THH, JEP, MH, CSF, and MKA revised and approved the manuscript.

## Supplementary Material

Supplemental data

Trial reporting checklists

ICMJE disclosure forms

## Figures and Tables

**Table 1:**
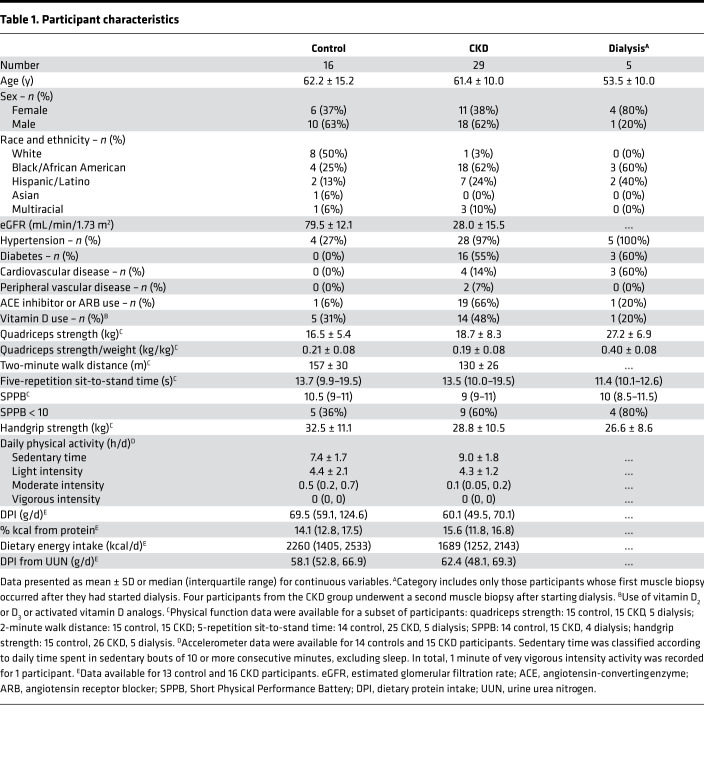
Participant characteristics

## Version 1 11/16/2021

In-Press Preview

## Version 2 12/22/2021

Electronic publication
