# Supplementary material for: Muscle fibrosis and maladaptation occur progressively in CKD and are rescued by dialysis
Source: JCI Insight. 2021 Dec 22;6(24):e150112. doi: 10.1172/jci.insight.150112 (PMC8783691; doi:10.1172/jci.insight.150112)
Supplement: Supplemental data [file jciinsight-6-150112-s076.pdf]

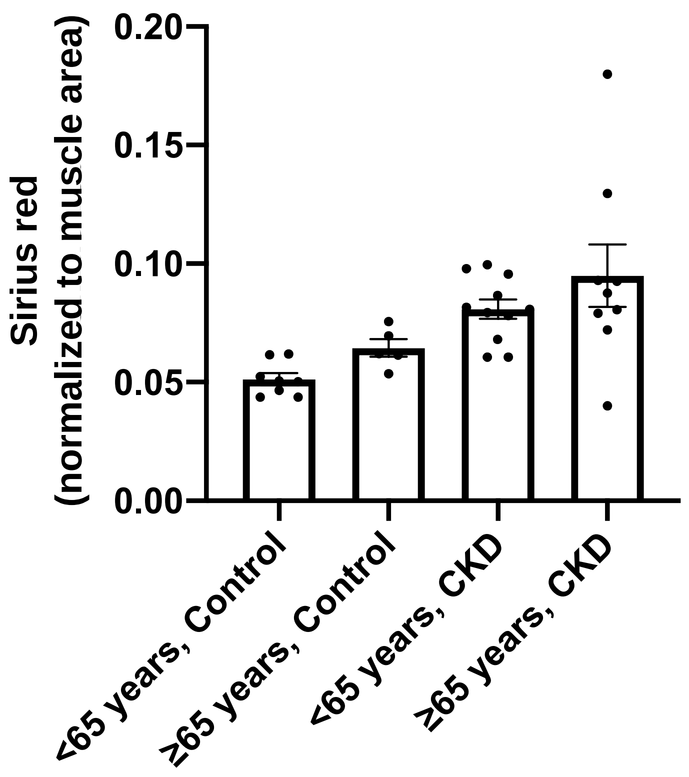

Supplementary Figure 1. Extracellular matrix collagen content is elevated in subjects with chronic kidney disease irrespective of age. n=33.

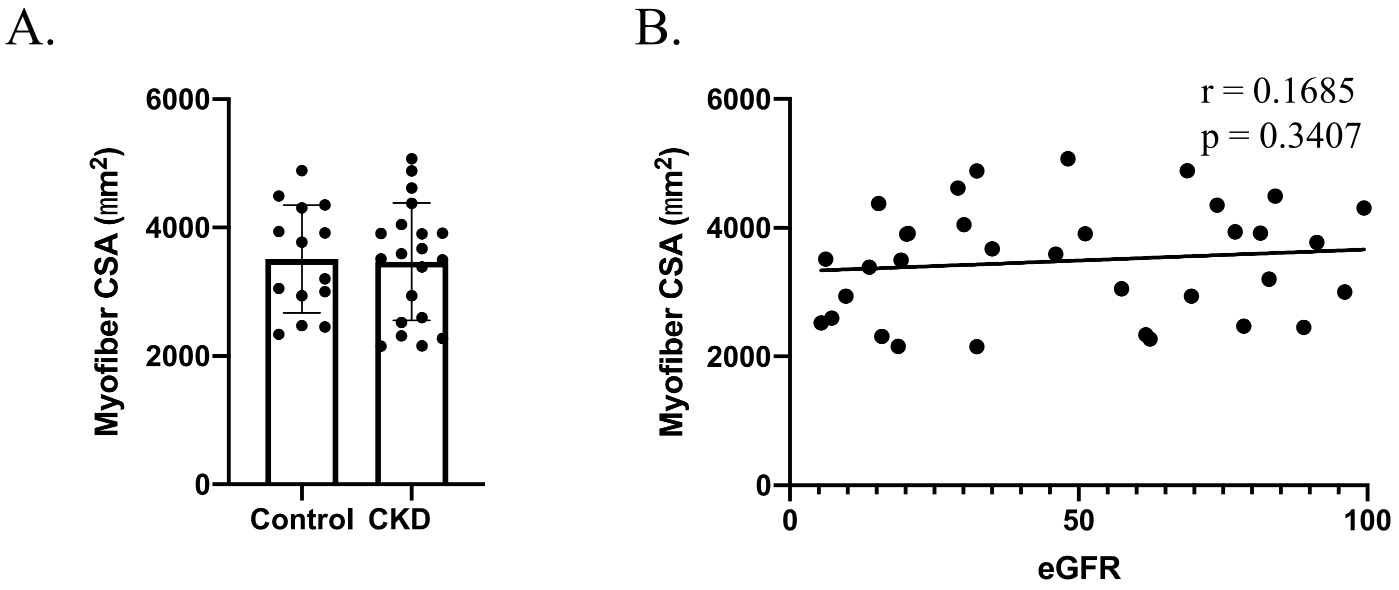

Supplementary Figure 2. Myofiber cross-sectional area (CSA) is not altered by chronic kidney disease. A. Myofiber CSA is not different in patients with CKD compared to healthy age-matched controls. B. Myofiber CSA is not associated with eGFR. n=33.

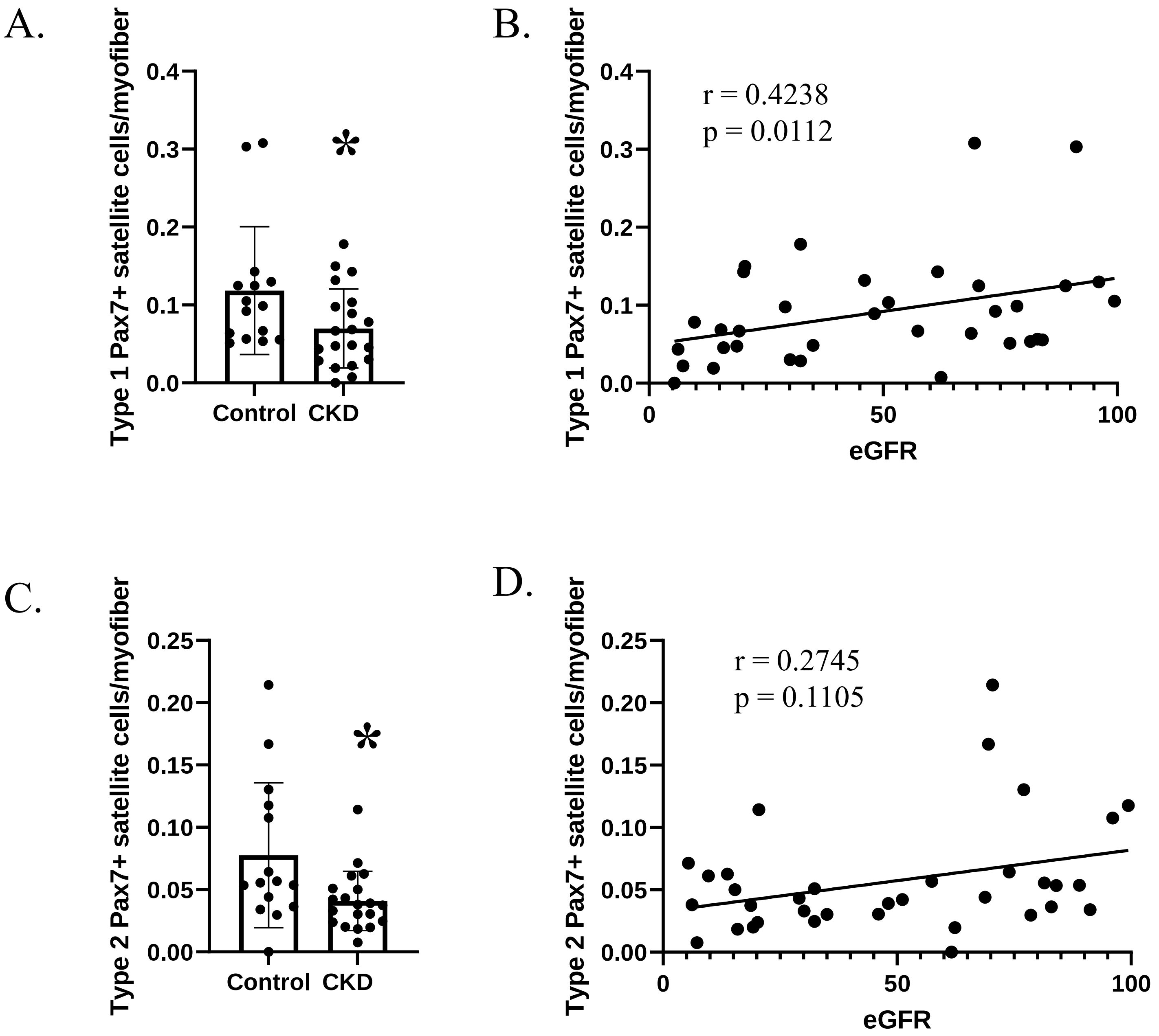

Supplementary Figure 3. Fiber type-specific satellite cell abundance is numerically lower in patients with CKD. A. Type 1 satellite cell abundance is numerically lower in patients with CKD compared to controls. B. Correlation between eGFR and type 1 satellite cell abundance. C. Type 2 satellite cell abundance is numerically lower in patients with CKD compared to controls. D. Correlation between eGFR and type 2 satellite cell abundance. n=33. *p<0.05 vs Control.

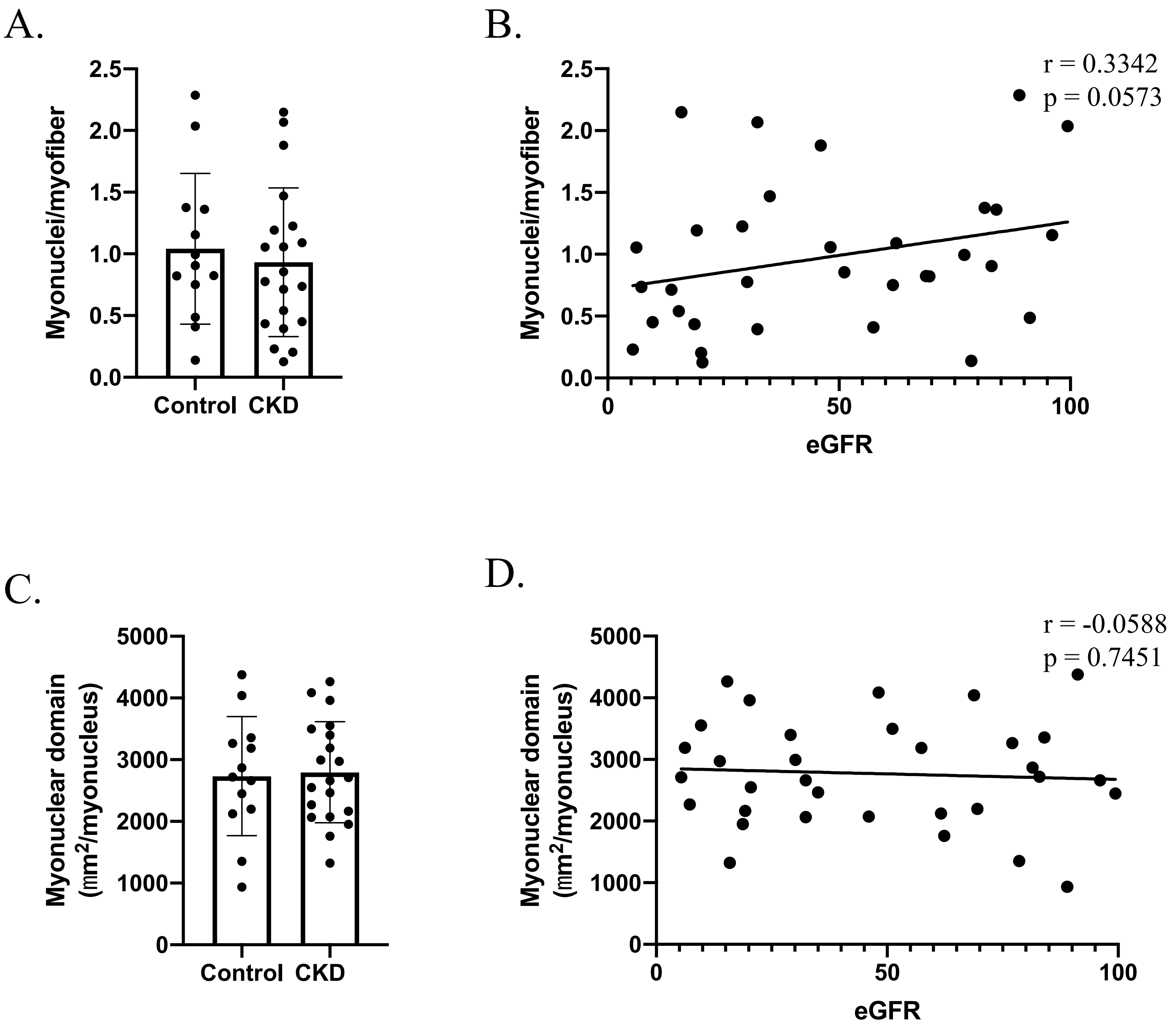

Supplementary Figure 4. Myonuclear density and myonuclear domain are not altered by CKD. A. Myonuclear density is not different in patients with CKD compared to controls. B. Correalation between myonuclear density and eGFR. C. Myonuclear domain is not different in patients with CKD compared to controls. D. Correlation between myonuclear domain and eGFR. n=33.

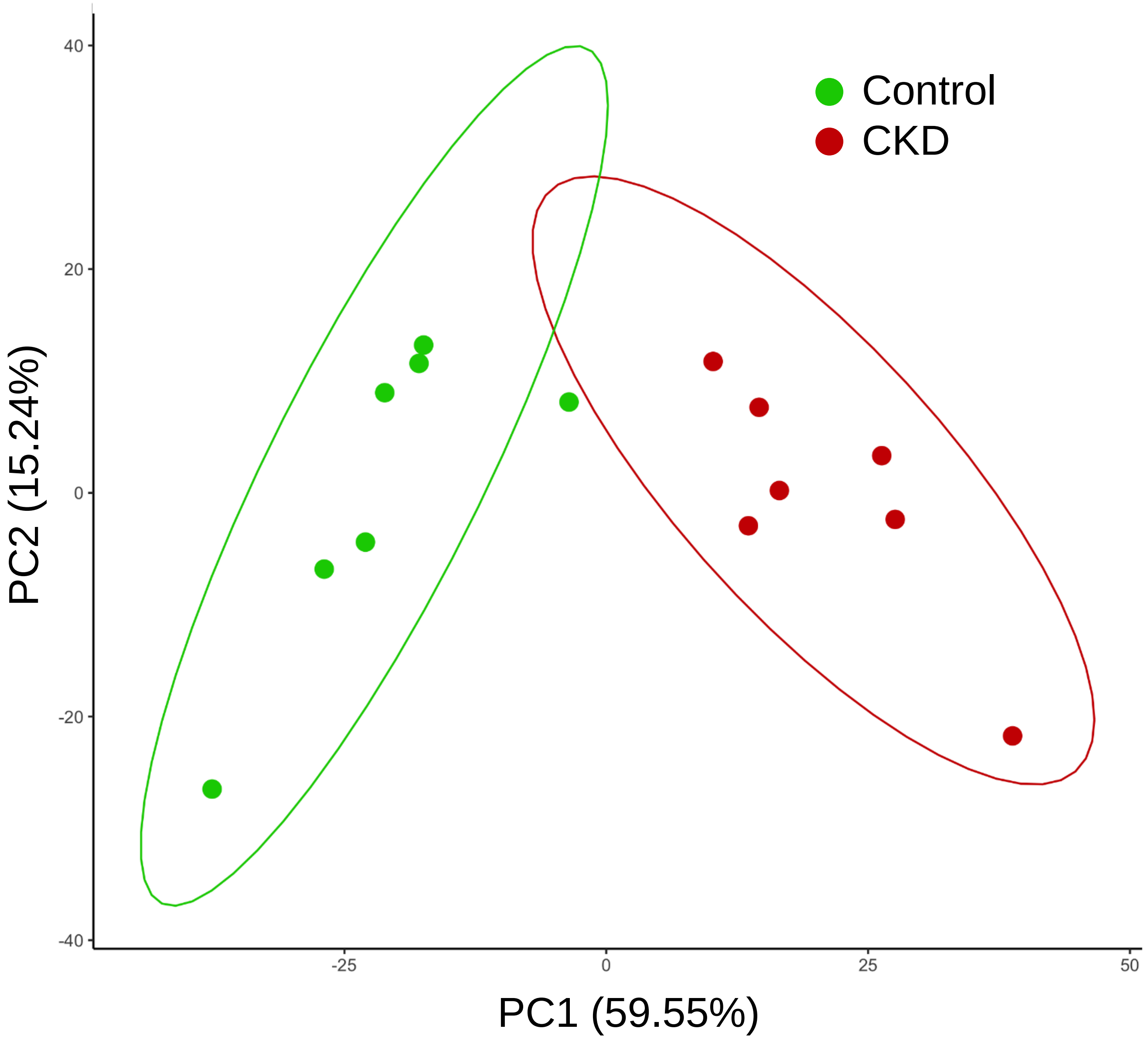

Supplementary Figure 5. Principal component analysis of skeletal muscle transcriptome of CKD patients compared to healthy individuals. n=7 Control; 7 CKD.

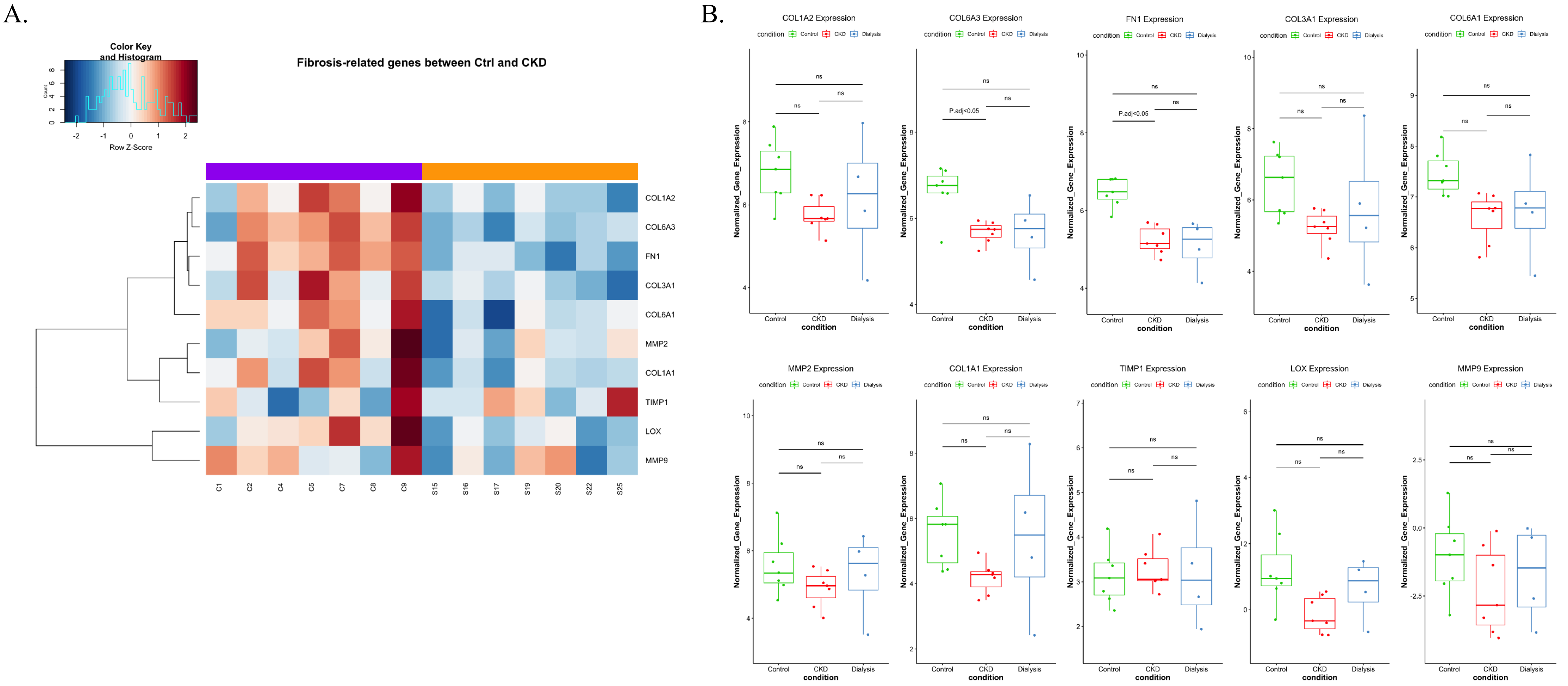

Supplementary Figure 6. Select fibrosis-related genes between control and CKD subjects. A. Heatmap indicating expression of select fibrosis-related genes. B. Box plots showing expression of select fibrosis-related genes. Orange bar=CKD, purple bar=Control. n=7 Control, n=7 CKD, n=4 Dialysis.

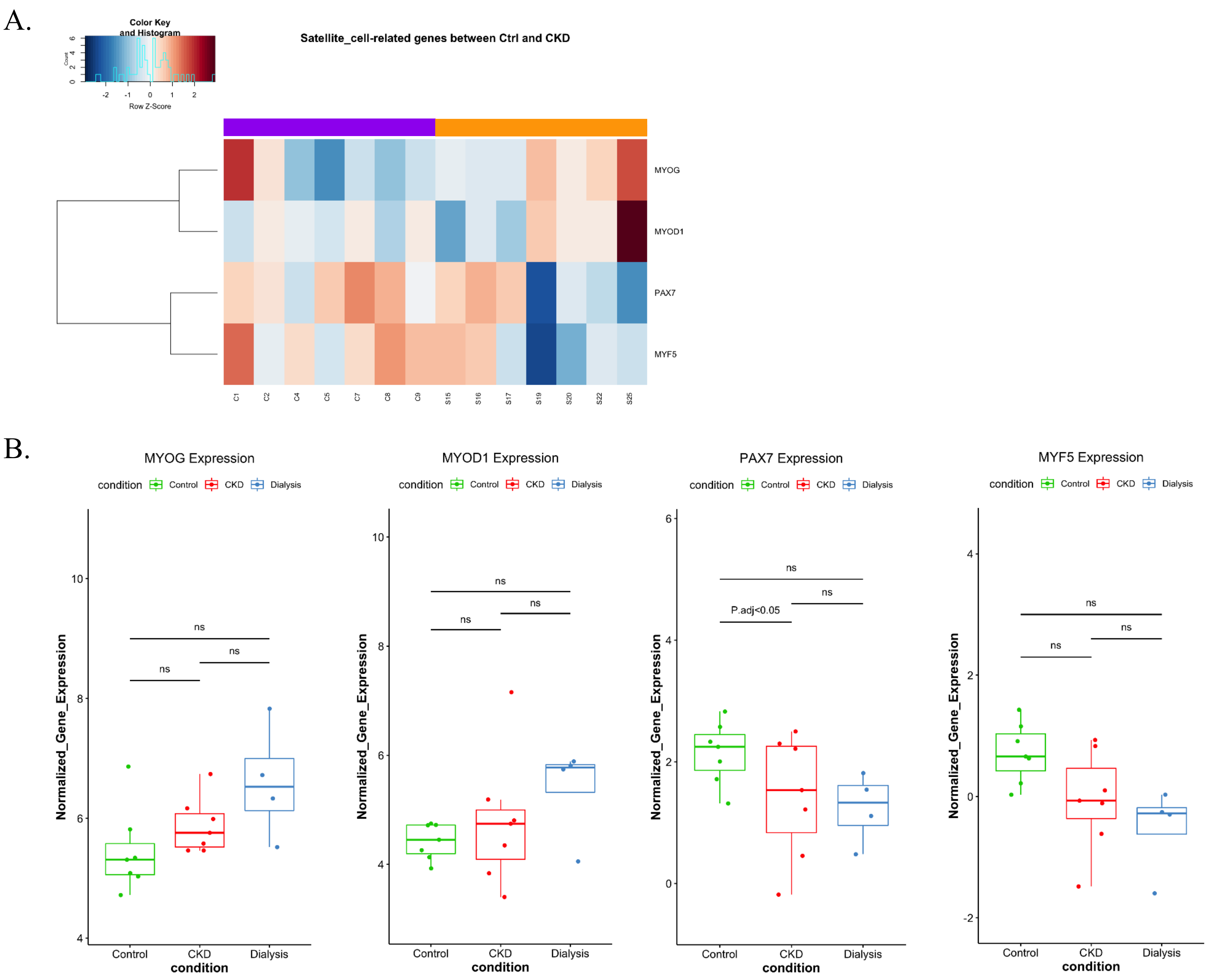

Supplementary Figure 7. CKD negatively impacts PAX7 transcription with no significant alteration in other satellite cell-related transcripts. A. Heatmap indicating expression of select satellite cell-related genes. B. PAX7 expression is lower is patients with CKD compared to controls, while other satellite cell-related transcripts are not significantly different. Orange bar=CKD, purple bar=Control. n=7 Control, n=7 CKD, n=4 Dialysis.

A.

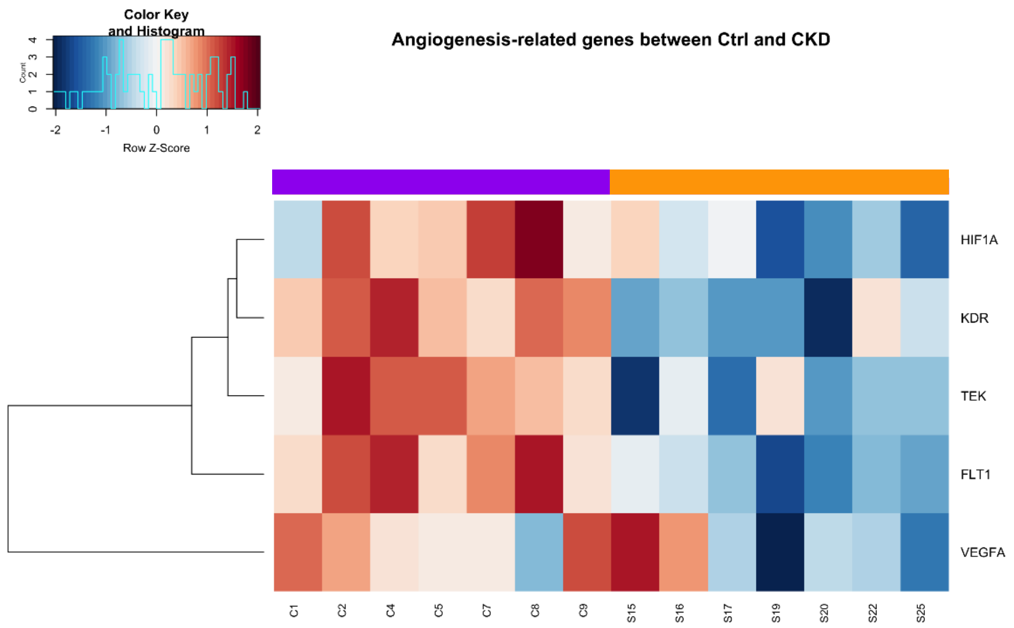

B.

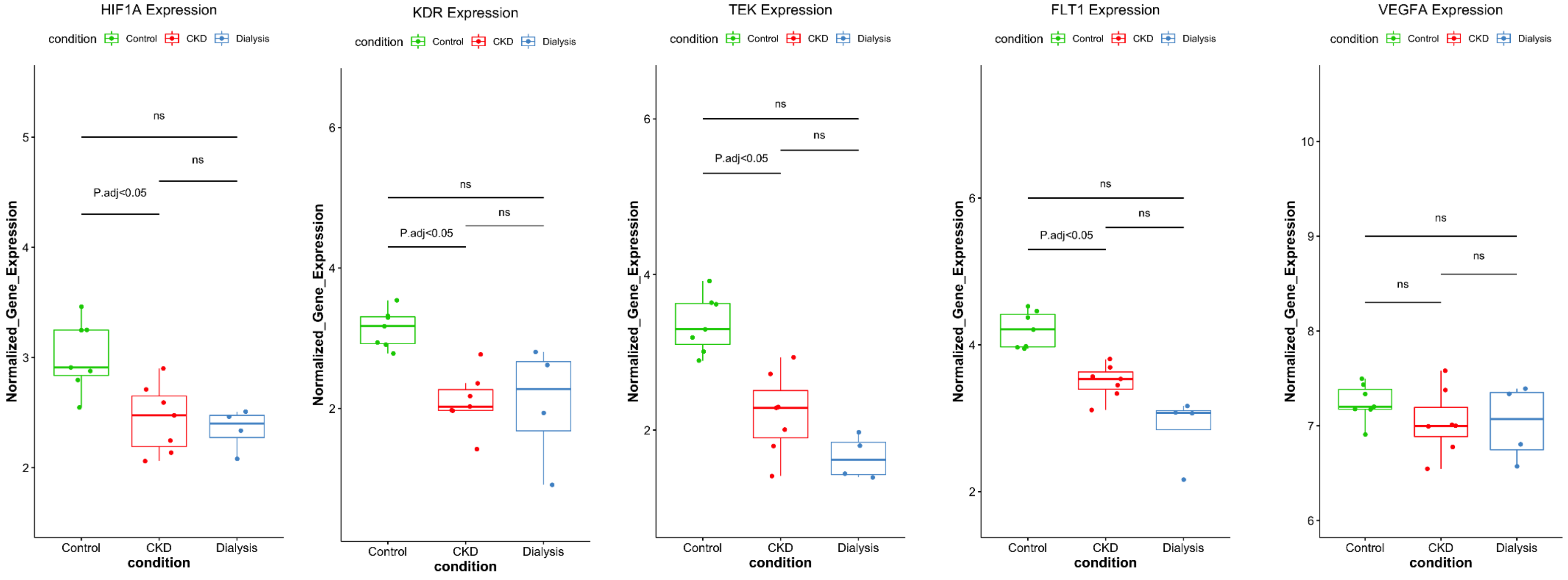

Supplementary Figure 8. CKD negatively impacts many angiogenesis-related transcripts. A. Heatmap indicating expression of select angiogenesis-related genes. B. Expression is significantly lower for most individual angiogenesis-related genes in patients with CKD compared to controls. Orange bar=CKD, purple bar=Control. n=7 Control, n=7 CKD, n=4 Dialysis.

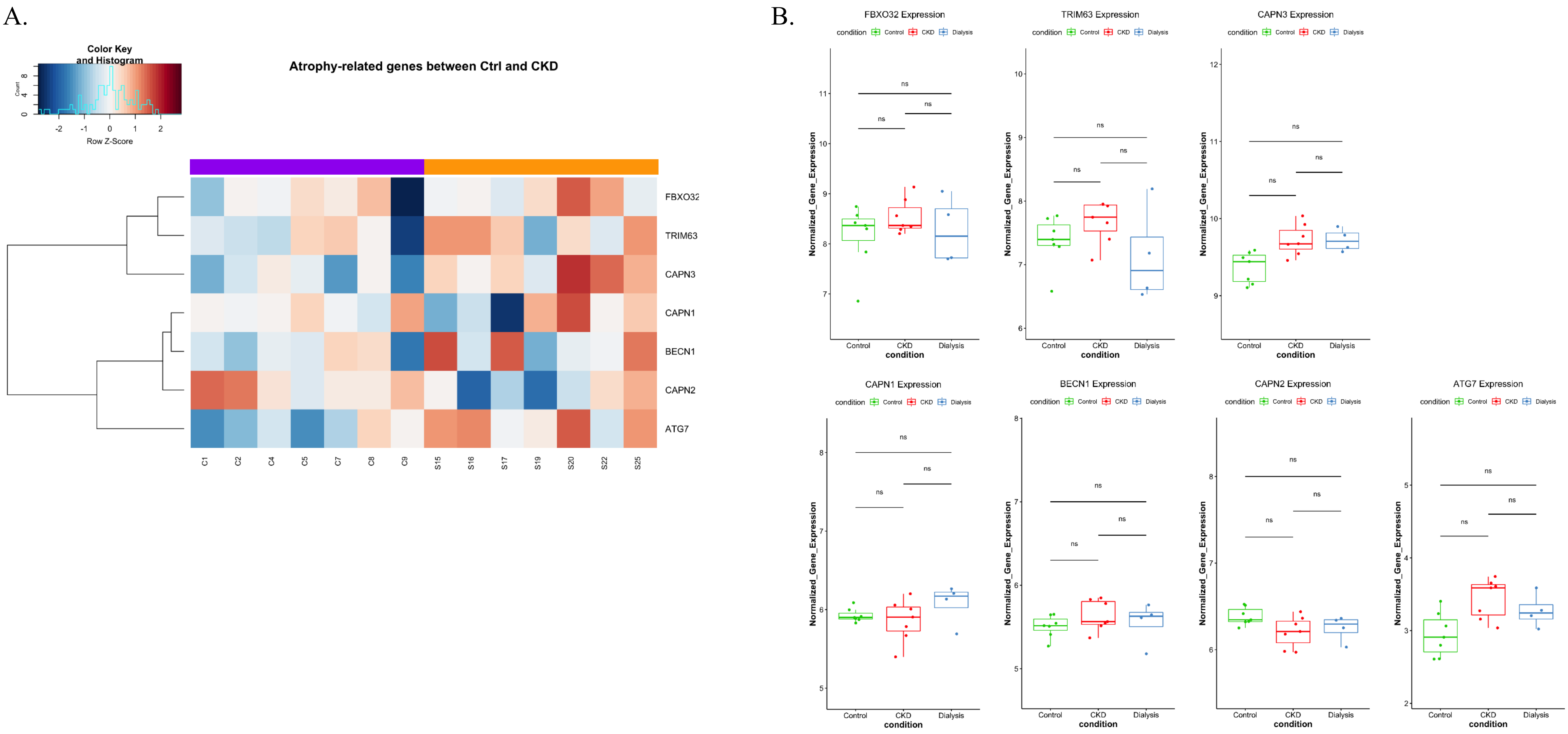

Supplementary Figure 9. Select atrophy-related transcript levels between control and CKD subjects. A. Heatmap indicating expression of select atrophy-related genes. B. Expression is not significantly altered for individual atrophy-related genes. Orange bar=CKD, purple bar=Control. n=7 Control, n=7 CKD, n=4 Dialysis.

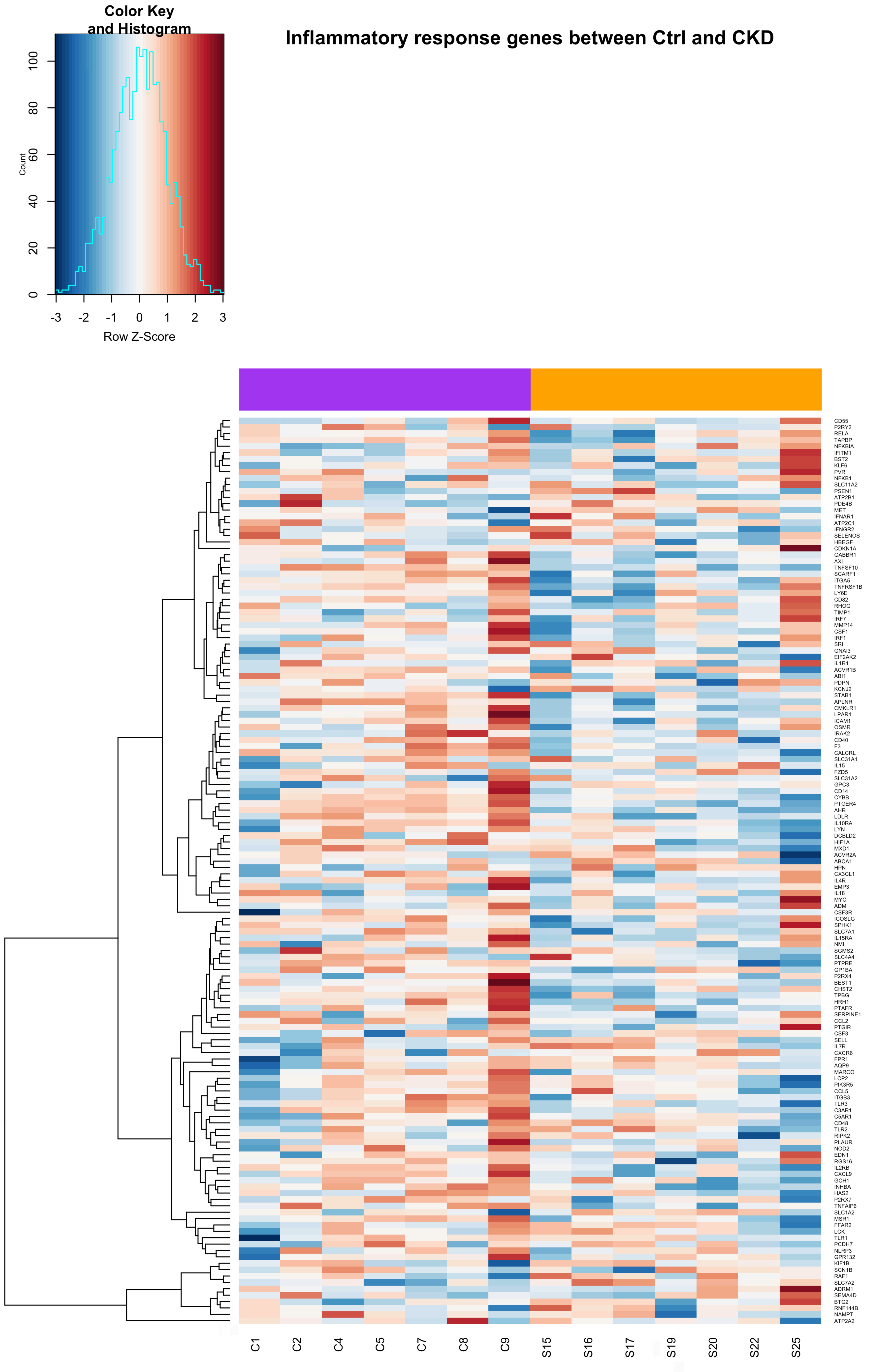

Supplementary Figure 10. Heatmap indicating expression of inflammation-related genes. Orange bar=CKD, purple bar=Control. n=7 Control, n=7 CKD.

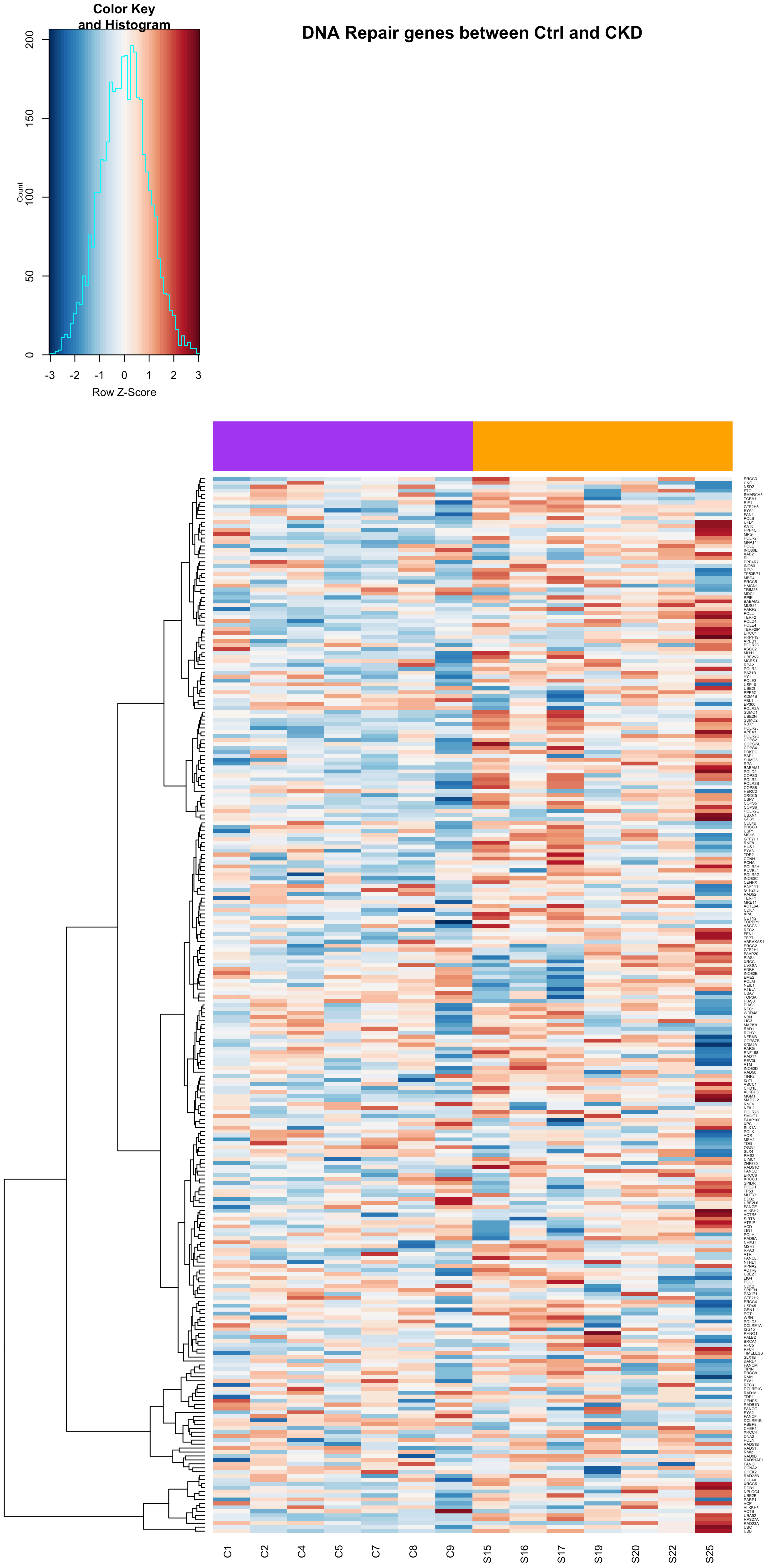

Supplementary Figure 11. Heatmap indicating expression of DNA repair-related genes. Orange bar=CKD, purple bar=Control. n=7 Control, n=7 CKD.

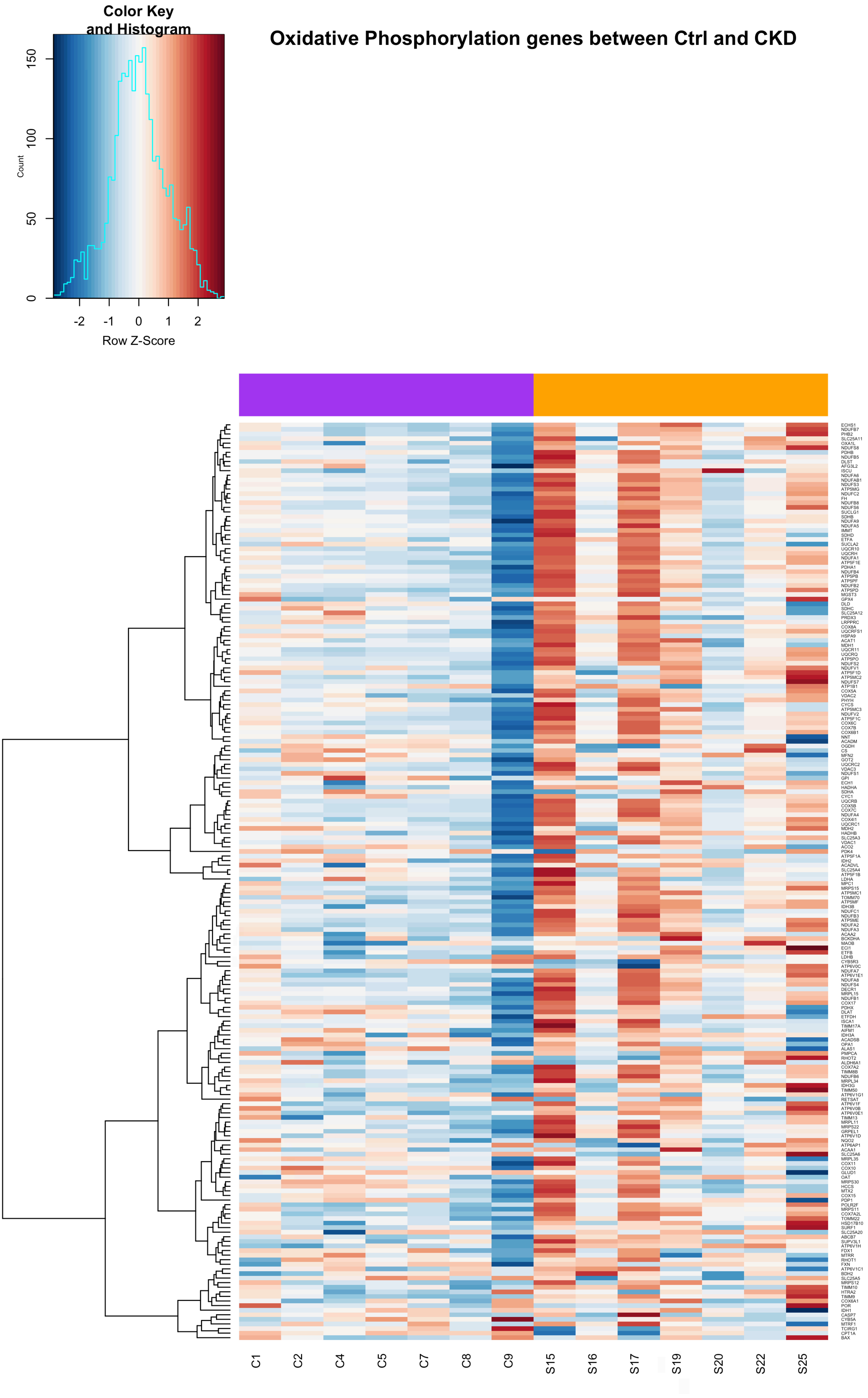

Supplementary Figure 12. Heatmap indicating expression of oxidative phosphorylation-related genes. Orange bar=CKD, purple bar=Control. n=7 Control, n=7 CKD.

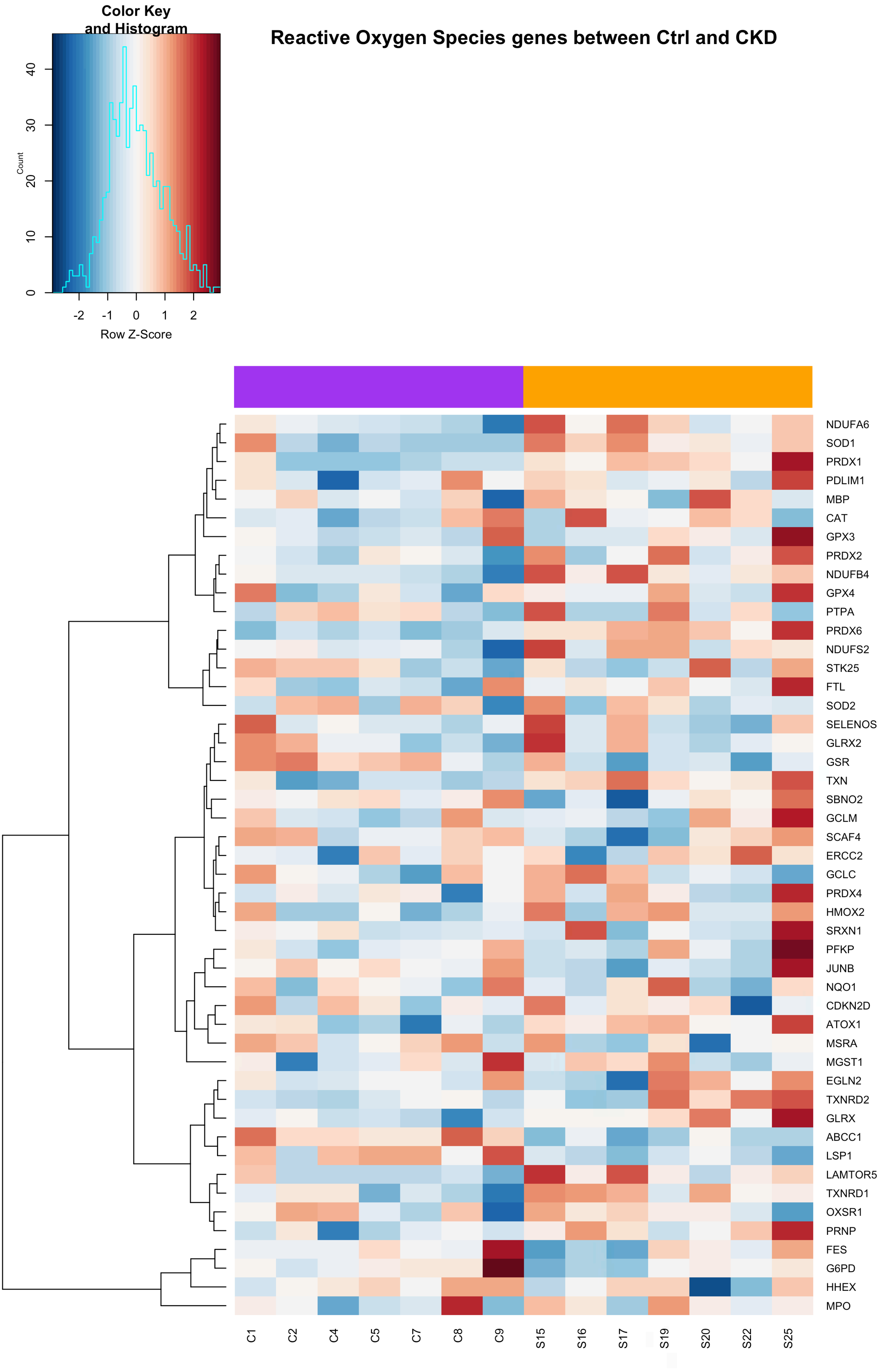

Supplementary Figure 13. Heatmap indicating expression of reactive oxygen species-related genes. Orange bar=CKD, purple bar=Control. n=7 Control, n=7 CKD.

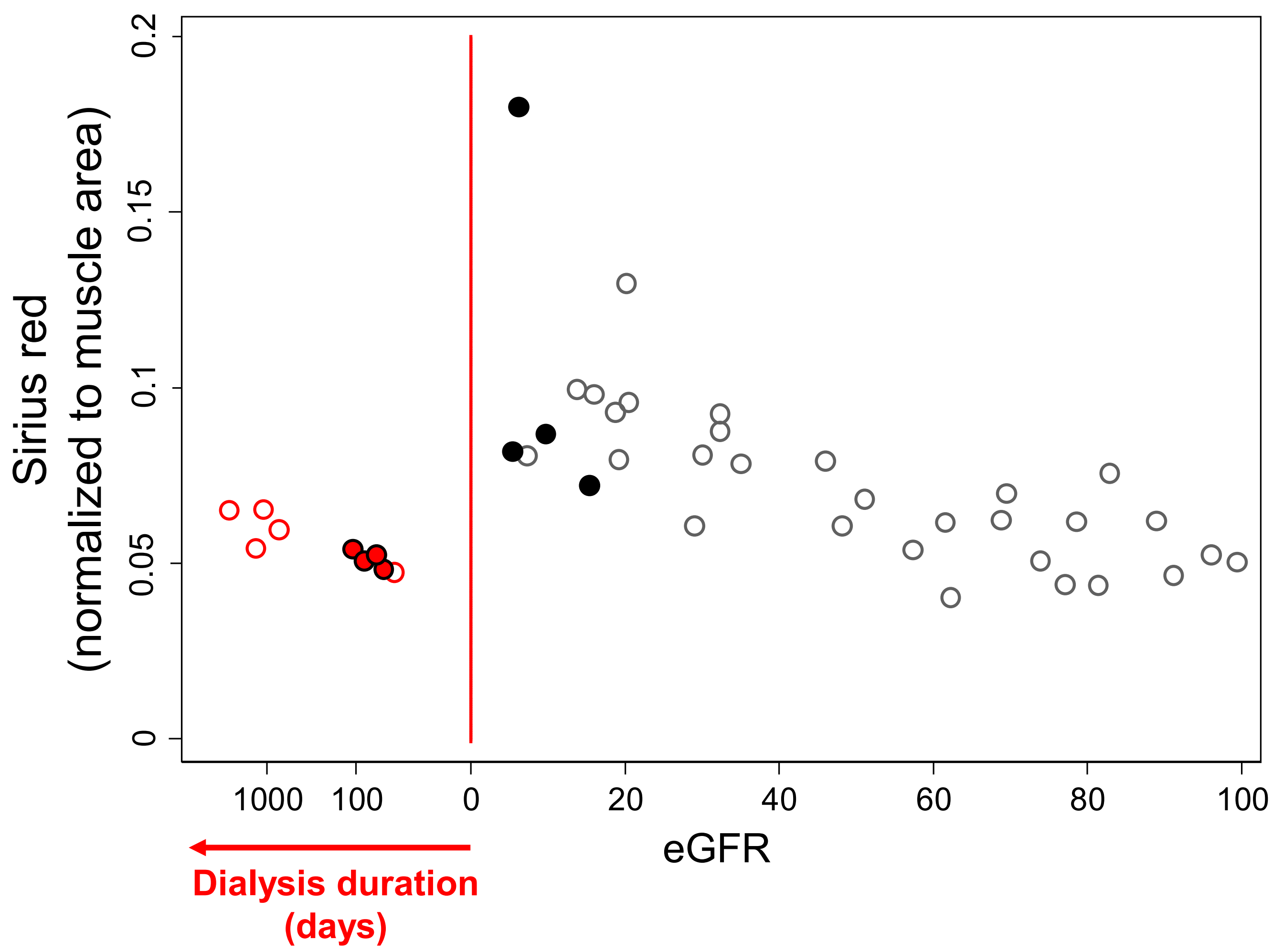

Supplementary Figure 14. Post-dialysis muscle collagen content is decreased in patients with CKD compared to muscle biopsied before initiation of dialysis. Red = Dialysis. Solid circles = paired samples. n=38 with 4 paired subjects.

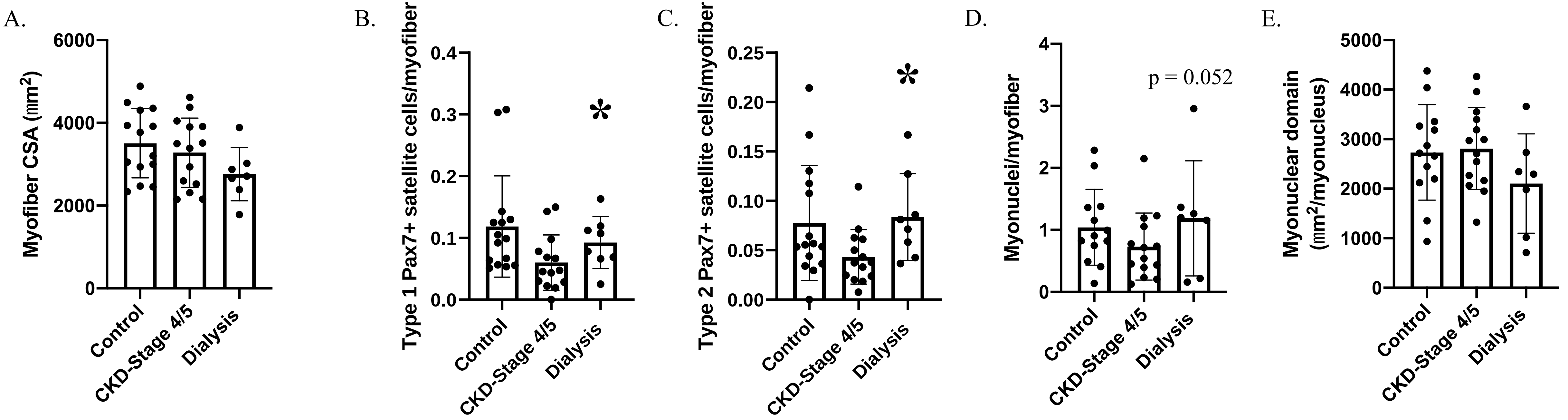

Supplemental Figure 15. The impact of dialysis on myofiber cross-sectional area (CSA), fiber type specific satellite cell abundance, and myonuclear content. A. Myofiber CSA in control, advanced stage 4/5 CKD and dialysisis subjects. B. Type 1 satellite cell abundance is elevated in patients with CKD undergoing dialysis compared to patients with advanced stage 4/5 CKD not undergoing dialysis. C. F. Type 2 satellite cell abundance is elevated in patients with CKD undergoing dialysis compared to stage 4/5 patients with CKD not undergoing dialysis. D. Myonuclear density in control, advanced stage 4/5 CKD and dialysis subjects. E. Myonuclear domain in control, advanced stage 4/5 CKD and dialysisis subjects. n=38. *p<0.05 compared to CKD-Stage 4/5.

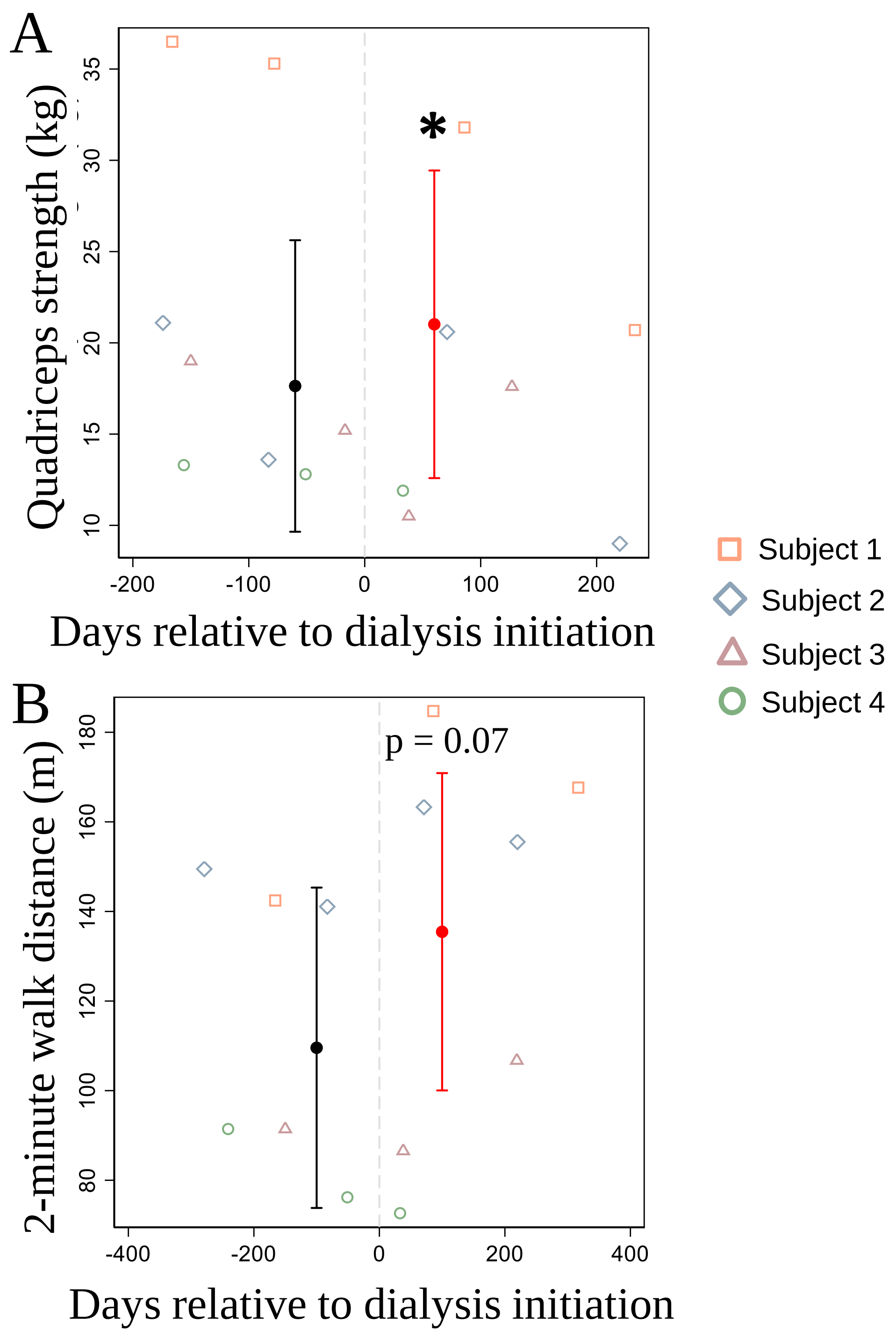

Supplementary Figure 16. Physical function in patients with CKD before and after initiation of dialysis. A. Adjusted quadriceps strength in patients with CKD before and after initiation of dialysis. B. Adjusted 2-minute walk performance in patients with CKD before and after initiation of dialysis. n=4. *p<0.05 compared to pre-dialysis.

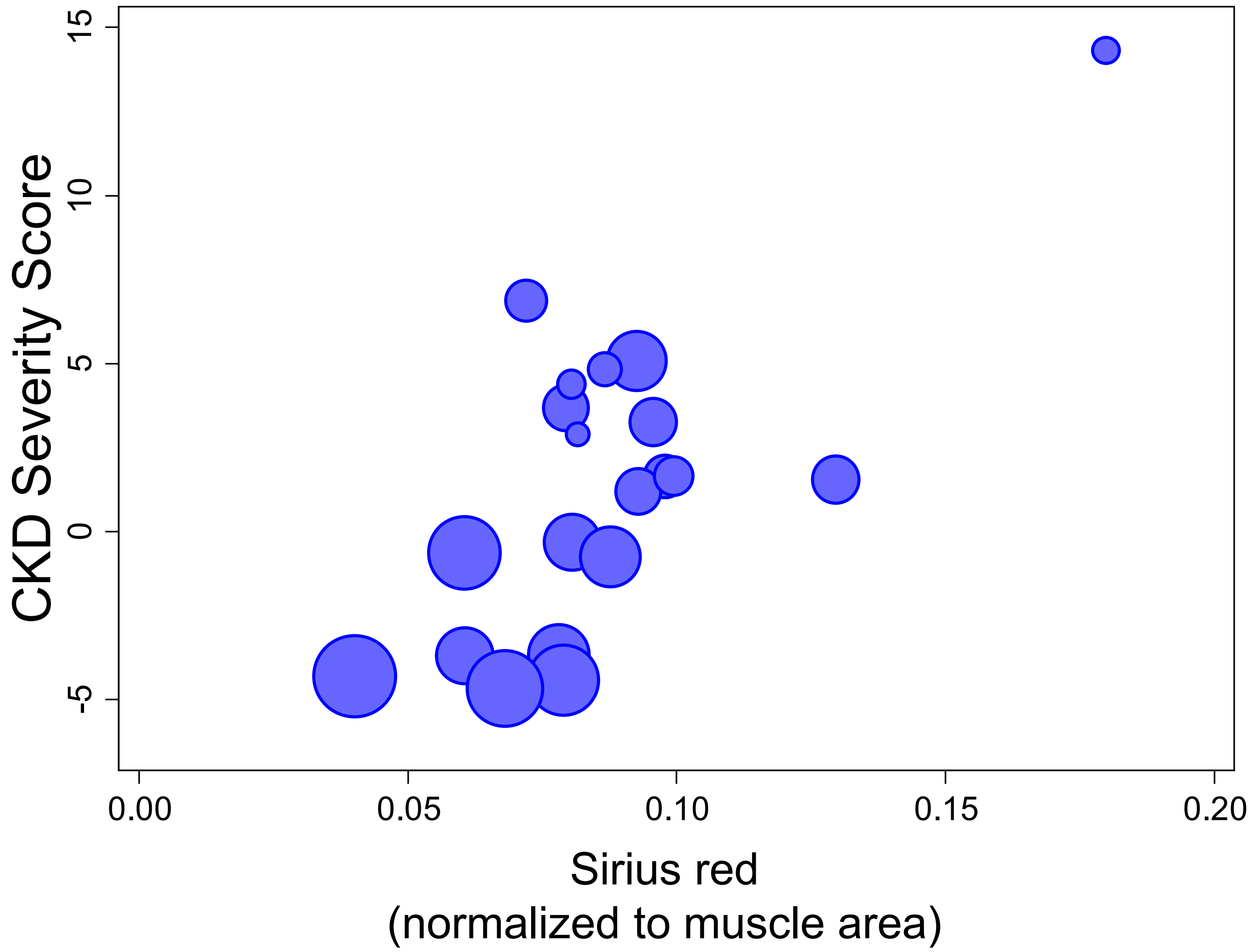

Supplementary Figure 17. Interrelationship of extracellular matrix collagen content, kidney function, and CKD severity score. The size of each data point is weighted by the estimated GFR, e.g. larger points correspond to higher GFR. The CKD severity score was calculated as the sum of standardized differences in serum bicarbonate, potassium, calcium, phosphate, parathyroid hormone, and hemoglobin. n=20.

Supplementary Table 1. Associations of eGFR (per 10 mL/min/1.73m^2) with histological parameters

	Unadjusted		Adjusted	
	Coefficient (95% CI)	p	Coefficient (95% CI)	p
Sirius red (normalized to muscle area)	-0.6 (-0.9 to -0.4)	<0.0001	-0.5 (-0.9 to -0.1)	0.012
Densely packed collagen (normalized to muscle area)*	-0.3 (-0.4 to -0.2)	<0.0001	-0.3 (-0.4 to -0.1)	0.014
Loosely packed collagen (normalized to muscle area)*	-0.1 (-0.2 to -0.0)	0.016	-0.2 (-0.3 to 0.0)	0.076
Myofiber cross sectional area (μm^2)	34.5 (-67.8 to 136.8)	0.497	115.1 (-34.3 to 264.6)	0.125
Pax7+ satellite cells/myofiber*	0.1 (0.0 to 0.2)	0.007	0.1 (0.01 to 0.2)	0.035
Type 1 Pax7+ satellite cells/myofiber	0.02 (0.002 to 0.03)	0.028	0.009 (-0.01 to 0.03)	0.420
Type 2 Pax7+ satellite cells/myofiber	0.01 (-0.0002 to 0.02)	0.054	0.01 (-0.004 to 0.03)	0.142
Myonuclei/myofiber	0.1 (-0.01 to 0.1)	0.116	0.1 (-0.04 to 0.2)	0.230
Myonuclear domain (μm^2/myonucleus)	-18.1 (-121.7 to 85.5)	0.724	3.9 (-158.7 to166.6)	0.961
Satellite cell-capillary distance (μm)*	-0.1 (-0.1 to -0.02)	0.008	-0.1 (-0.2 to -0.1)	0.001
Capillary-to-fiber perimeter exchange ratio (capillaries/1000 μm)	0.3 (0.2 to 0.4)	<0.0001	0.3 (0.1 to 0.4)	0.0009

Abbreviations: eGFR, estimated glomerular filtration rate; CI, confidence interval.
All results from linear regression models with histological parameter as dependent variable and eGFR as independent variable.
Adjusted results from models adjusted for age, sex, race, and diabetes, hypertension, and cardiovascular disease status.
*Dependent variable was log-transformed.

Supplementary Table 2. Differentially expressed genes (DEG) between CKD and control.

ENSEMBL_ID	HUGO Gene Symbol	Approved Gene Name	HGNC ID	Location	logFC	AveExpr	t	P.Value	adj.P.Val	B
ENSG00000069122.19_ADGRF5	ADGRF5	adhesion G protein-coupled receptor F5	HGNC:19030	6p12.3	-1.2106942	4.18576718	-9.021991	1.90E-08	0.00028531	9.40175963
ENSG00000251322.7_SHANK3	SHANK3	SH3 and multiple ankyrin repeat domains 3	HGNC:14294	22q13.33	-1.2118483	5.04184589	-8.3482065	6.51E-08	0.00048826	8.3200914
ENSG00000128567.17_PODXL	PODXL	podocalyxin like	HGNC:9171	7q32.3	-1.3698975	4.4409812	-7.692881	2.28E-07	0.00114002	7.14967746
ENSG00000133561.15_GIMAP6	GIMAP6	GTPase, IMAP family member 6	HGNC:21918	7q36.1	-1.1170069	3.65123552	-7.2731588	5.25E-07	0.00132431	6.34293101
ENSG00000179144.5_GIMAP7	GIMAP7	GTPase, IMAP family member 7	HGNC:22404	7q36.1	-0.9030641	2.64920203	-7.2681721	5.30E-07	0.00132431	6.21092915
ENSG00000111252.10_SH2B3	SH2B3	SH2B adaptor protein 3	HGNC:29605	12q24.12	-0.9579406	3.27609788	-7.1865094	6.25E-07	0.00133795	6.14963336
ENSG00000173706.14_HEG1	HEG1	heart development protein with EGF like domains 1	HGNC:29227	3q21.2	-1.0585684	3.96291082	-7.0801465	7.76E-07	0.00133795	6.00067763
ENSG00000142192.21_APP	APP	amyloid beta precursor protein	HGNC:620	21q21.3	-1.0570508	5.8656643	-6.965123	9.81E-07	0.00133795	5.80540284
ENSG00000088387.19_DOCK9	DOCK9	dedicator of cytokinesis 9	HGNC:14132	13q32.3	-0.8292133	3.41222043	-6.9823362	9.47E-07	0.00133795	5.78712552
ENSG00000205978.6_NYNRIN	NYNRIN	NYN domain and retroviral integrase containing	HGNC:20165	14q12	-1.5799027	2.45575406	-7.0175949	8.81E-07	0.00133795	5.69125358
ENSG00000214357.8_NEURL1B	NEURL1B	neuralized E3 ubiquitin protein ligase 1B	HGNC:35422	5q35.1	-1.1100071	3.84119345	-6.8462103	1.25E-06	0.00156731	5.54988891
ENSG00000165810.17_BTNL9	BTNL9	butyrophilin like 9	HGNC:24176	5q35.3	-1.394087	4.68599307	-6.517927	2.49E-06	0.00189167	4.9234907
ENSG00000171115.4_GIMAP8	GIMAP8	GTPase, IMAP family member 8	HGNC:21792	7q36.1	-0.8659558	3.10481551	-6.5389326	2.38E-06	0.00189167	4.91555761
ENSG00000037280.16_FLT4	FLT4	fms related receptor tyrosine kinase 4	HGNC:3767	5q35.3	-1.4183613	2.2697795	-6.6069508	2.07E-06	0.00189167	4.90873823
ENSG00000076067.13_RBMS2	RBMS2	RNA binding motif single stranded interacting protein 2	HGNC:9909	12q13.3	-0.8392912	3.89218297	-6.5121862	2.52E-06	0.00189167	4.90217184
ENSG00000170989.9_S1PR1	S1PR1	sphingosine-1-phosphate receptor 1	HGNC:3165	1p21.2	-1.1144055	3.08079865	-6.5125406	2.52E-06	0.00189167	4.86464544
ENSG00000169291.10_SHE	SHE	Src homology 2 domain containing E	HGNC:27004	1q21.3	-1.0551347	2.4216914	-6.5342662	2.41E-06	0.00189167	4.8232022
ENSG00000158352.15_SHROOM4	SHROOM4	shroom family member 4	HGNC:29215	Xp11.22	-1.1484479	2.27483866	-6.5401039	2.38E-06	0.00189167	4.80985133
ENSG00000031081.10_ARHGAP31	ARHGAP31	Rho GTPase activating protein 31	HGNC:29216	3q13.32-q13.	-1.2968455	2.50407403	-6.4402596	2.94E-06	0.00209823	4.65376991
ENSG00000116962.15_NID1	NID1	nidogen 1	HGNC:7821	1q42.3	-1.471553	3.63133203	-6.3892909	3.28E-06	0.00210289	4.64472653
ENSG00000127329.15_PTPRB	PTPRB	protein tyrosine phosphatase receptor type B	HGNC:9665	12q15	-1.1015828	4.00736329	-6.3797174	3.34E-06	0.00210289	4.63951668
ENSG00000166341.8_DCHS1	DCHS1	dachsous cadherin-related 1	HGNC:13681	11p15.4	-1.5854788	3.2630433	-6.3764672	3.37E-06	0.00210289	4.60059953
ENSG00000179776.19_CDH5	CDH5	cadherin 5	HGNC:1764	16q21	-1.2851393	4.67586958	-6.3400922	3.63E-06	0.00217757	4.56876113
ENSG00000106546.14_AHR	AHR	aryl hydrocarbon receptor	HGNC:348	7p21.1	-1.6068804	0.91864387	-6.6232289	2.00E-06	0.00189167	4.47697126
ENSG00000178695.5_KCTD12	KCTD12	potassium channel tetramerization domain containing 12	HGNC:14678	13q22.3	-1.0237906	3.76595161	-6.26585	4.26E-06	0.00245938	4.40795827
ENSG00000099250.18_NRP1	NRP1	neuropilin 1	HGNC:8004	10p11.22	-1.0652634	4.68986532	-6.2247121	4.66E-06	0.00258718	4.33387015
ENSG00000272047.2_GTF2H5	GTF2H5	general transcription factor IIH subunit 5	HGNC:21157	6q25.3	0.66520675	4.77513271	6.09946228	6.11E-06	0.00315686	4.07808316
ENSG00000090975.12_PITPNM2	PITPNM2	phosphatidylinositol transfer protein membrane associated 2	HGNC:21044	12q24.31	-1.1717244	2.65613585	-6.1243851	5.78E-06	0.00309781	4.06060062
ENSG00000144713.12_RPL32	RPL32	ribosomal protein L32	HGNC:10336	3p25.2	0.85013396	8.76831	6.03905987	6.96E-06	0.00326178	3.9525533
ENSG00000150760.12_DOCK1	DOCK1	dedicator of cytokinesis 1	HGNC:2987	10q26.2	-1.1983502	2.46780327	-6.0744461	6.45E-06	0.00322178	3.94496637
ENSG00000101384.12_JAG1	JAG1	jagged canonical Notch ligand 1	HGNC:6188	20p12.2	-0.9393933	3.31359329	-6.0034396	7.52E-06	0.00341796	3.8662019
ENSG00000171960.11_PPIH	PPIH	peptidylprolyl isomerase H	HGNC:14651	1p34.2	1.01339473	2.06247323	6.04776691	6.83E-06	0.00326178	3.80584116
ENSG00000078596.11_ITM2A	ITM2A	integral membrane protein 2A	HGNC:6173	Xq21.1	-0.9391871	3.56979872	-5.9403549	8.63E-06	0.00380722	3.74437867
ENSG00000171858.18_RPS21	RPS21	ribosomal protein S21	HGNC:10409	20q13.33	0.89993908	7.89589051	5.91050362	9.22E-06	0.00394814	3.68786048
ENSG00000221983.7_UBA52	UBA52	ubiquitin A-52 residue ribosomal protein fusion product 1	HGNC:12458	19p13.1-p12	0.74661444	8.35944458	5.82413083	1.11E-05	0.00426254	3.50822601
ENSG00000178449.9_COX14	COX14	cytochrome c oxidase assembly factor COX14	HGNC:28216	12q13.12	0.7465524	5.5683276	5.82245562	1.12E-05	0.00426254	3.50369636
ENSG00000122026.10_RPL21	RPL21	ribosomal protein L21	HGNC:10313	13q12.2	0.72530009	8.62109644	5.81874444	1.13E-05	0.00426254	3.49707109
ENSG00000164125.15_GASK1B	GASK1B	golgi associated kinase 1B	HGNC:25312	4q32.1	-1.4763714	2.84093841	-5.8198391	1.12E-05	0.00426254	3.46933146
ENSG00000096433.11_ITPR3	ITPR3	inositol 1,4,5-trisphosphate receptor type 3	HGNC:6182	6p21.31	-1.1826544	2.9658644	-5.8119098	1.14E-05	0.00426254	3.4649626
ENSG00000100065.15_CARD10	CARD10	caspase recruitment domain family member 10	HGNC:16422	22q13.1	-1.2389964	2.95598871	-5.8035957	1.17E-05	0.00426254	3.43868316
ENSG00000169567.11_HINT1	HINT1	histidine triad nucleotide binding protein 1	HGNC:4912	5q23.3	0.66494432	6.58953624	5.78797863	1.21E-05	0.00429765	3.43151094
ENSG00000183011.13_NAA38	NAA38	N-alpha-acetyltransferase 38, NatC auxiliary subunit	HGNC:28212	17p13.1	0.83956575	5.30402534	5.77827007	1.23E-05	0.00429765	3.41135991
ENSG00000125810.10_CD93	CD93	CD93 molecule	HGNC:15855	20p11.21	-1.1493891	4.64584946	-5.7627686	1.28E-05	0.00434614	3.37988917
ENSG00000161791.14_FMNL3	FMNL3	formin like 3	HGNC:23698	12q13.12	-0.8712225	3.54431311	-5.7454976	1.32E-05	0.0044148	3.34156546
ENSG00000257315.2_ZBED6	ZBED6	zinc finger BED-type containing 6	HGNC:33273	1q32.1	6.33009678	-1.2721545	7.39247641	4.13E-07	0.00132431	3.30581311
ENSG00000148357.16_HMCN2	HMCN2	hemicentin 2	HGNC:21293	9q34.11	-1.3945726	5.38111206	-5.7254504	1.38E-05	0.0044185	3.30051062
ENSG00000166265.12_CYYR1	CYYR1	cysteine and tyrosine rich 1	HGNC:16274	21q21.3	-0.9155695	2.66052339	-5.7345526	1.36E-05	0.0044185	3.29299708
ENSG00000175899.14_A2M	A2M	alpha-2-macroglobulin	HGNC:7	12p13.31	-0.8328248	7.14349815	-5.6877011	1.51E-05	0.00470362	3.22128981
ENSG00000116016.14_EPAS1	EPAS1	endothelial PAS domain protein 1	HGNC:3374	2p21	-1.0075254	5.97363879	-5.6492238	1.64E-05	0.00501823	3.13927679
ENSG00000066056.14_TIE1	TIE1	tyrosine kinase with immunoglobulin like and EGF like domain	HGNC:11809	1p34.2	-1.1976617	3.42603944	-5.5852113	1.89E-05	0.00555919	3.00717177
ENSG00000163531.15_NFASC	NFASC	neurofascin	HGNC:29866	1q32.1	-1.1324967	2.41092124	-5.5910529	1.87E-05	0.00555919	2.98131325
ENSG00000111145.8_ELK3	ELK3	ETS transcription factor ELK3	HGNC:3325	12q23.1	-1.1717048	2.43822212	-5.569231	1.96E-05	0.00564997	2.93707385
ENSG00000233927.5_RPS28	RPS28	ribosomal protein S28	HGNC:10418	19p13.2	0.74067915	8.19842878	5.54189373	2.08E-05	0.00577523	2.91436216
ENSG00000108219.15_TSPAN14	TSPAN14	tetraspanin 14	HGNC:23303	10q23.1	-0.967872	3.82242007	-5.5384266	2.10E-05	0.00577523	2.91028288
ENSG00000138326.19_RPS24	RPS24	ribosomal protein S24	HGNC:10411	10q22.3	0.74584401	8.84154364	5.53426836	2.12E-05	0.00577523	2.89998571
ENSG00000111678.11_C12orf57	C12orf57	chromosome 12 open reading frame 57	HGNC:29521	12p13.31	0.70773737	5.33632084	5.47989143	2.39E-05	0.00629316	2.77975176
ENSG00000130147.16_SH3BP4	SH3BP4	SH3 domain binding protein 4	HGNC:10826	2q37.2	-1.0682025	1.76377082	-5.5218553	2.18E-05	0.0058316	2.76971794
ENSG00000121858.11_TNFSF10	TNFSF10	TNF superfamily member 10	HGNC:11925	3q26	-0.8656956	3.45432112	-5.4469672	2.58E-05	0.00646727	2.71799528
ENSG00000123349.14_PFDN5	PFDN5	prefoldin subunit 5	HGNC:8869	12q13.13	0.70767805	6.80222191	5.45065537	2.55E-05	0.00646727	2.71731987
ENSG00000198561.13_CTNND1	CTNND1	catenin delta 1	HGNC:2515	11q12.1	-0.7174858	5.01470939	-5.4448252	2.59E-05	0.00646727	2.70579357
ENSG00000148400.11_NOTCH1	NOTCH1	notch receptor 1	HGNC:7881	9q34.3	-1.244921	3.68554562	-5.4321236	2.66E-05	0.00651099	2.68681084
ENSG00000103196.12_CRISPLD2	CRISPLD2	cysteine rich secretory protein LCCL domain containing 2	HGNC:25248	16q24.1	-1.1931733	2.79406144	-5.4272089	2.69E-05	0.00651099	2.66570762
ENSG00000120156.21_TEK	TEK	TEK receptor tyrosine kinase	HGNC:11724	9p21.2	-1.139331	2.53443053	-5.3924568	2.91E-05	0.00665358	2.58815961
ENSG00000267060.5_PTGES3L	PTGES3L	prostaglandin E synthase 3 like	HGNC:43943	17q21.31	0.89903454	5.30557091	5.38840854	2.94E-05	0.00665358	2.58441947
ENSG00000183978.8_COA3	COA3	cytochrome c oxidase assembly factor 3	HGNC:24990	17q21.2	0.75840884	4.99826752	5.38506649	2.96E-05	0.00665358	2.57818254
ENSG00000169504.15_CLIC4	CLIC4	chloride intracellular channel 4	HGNC:13518	1p36.11	-0.8417987	4.31338252	-5.3789238	3.00E-05	0.00665358	2.56817586
ENSG00000136942.15_RPL35	RPL35	ribosomal protein L35	HGNC:10344	9q33.3	0.79432083	8.65750629	5.35761369	3.15E-05	0.00684138	2.5235559
ENSG00000064989.13_CALCRL	CALCRL	calcitonin receptor like receptor	HGNC:16709	2q32.1	-1.1541373	1.83507718	-5.3764661	3.02E-05	0.00665358	2.49218752
ENSG00000074416.14_MGLL	MGLL	monoglyceride lipase	HGNC:17038	3q21.3	-0.979514	5.79489607	-5.3377271	3.29E-05	0.00699291	2.47498886
ENSG00000147123.10_NDUFB11	NDUFB11	NADH:ubiquinone oxidoreductase subunit B11	HGNC:20372	Xp11.3	0.72489674	7.38757573	5.33520771	3.31E-05	0.00699291	2.47104059
ENSG00000142798.20_HSPG2	HSPG2	heparan sulfate proteoglycan 2	HGNC:5273	1p36.12	-1.4205899	6.88480258	-5.3210252	3.42E-05	0.00711986	2.44062105
ENSG00000182533.6_CAV3	CAV3	caveolin 3	HGNC:1529	3p25.3	0.74658292	6.08036639	5.30831678	3.52E-05	0.00717945	2.41142357
ENSG00000074527.12_NTN4	NTN4	netrin 4	HGNC:13658	12q22	-1.021249	2.68965518	-5.3025208	3.56E-05	0.00717945	2.40977297
ENSG00000213741.10_RPS29	RPS29	ribosomal protein S29	HGNC:10419	14q21.3	0.82690122	7.31436219	5.29425805	3.63E-05	0.00717945	2.38280467
ENSG00000130255.13_RPL36	RPL36	ribosomal protein L36	HGNC:13631	19p13.3	0.79220321	7.21315452	5.29323387	3.64E-05	0.00717945	2.38031325
ENSG00000154065.17_ANKRD29	ANKRD29	ankyrin repeat domain 29	HGNC:27110	18q11.2	-1.0881912	0.9637588	-5.4032214	2.84E-05	0.00665358	2.37272899
ENSG00000188352.12_FOCAD	FOCAD	focadhesin	HGNC:23377	9p21.3	0.72404353	3.80788855	5.28210449	3.73E-05	0.00717945	2.36746558
ENSG00000197958.13_RPL12	RPL12	ribosomal protein L12	HGNC:10302	9q33.3	0.73921787	8.51168035	5.2818757	3.73E-05	0.00717945	2.36070124
ENSG00000102309.13_PIN4	PIN4	peptidylprolyl cis/trans isomerase, NIMA-interacting 4	HGNC:8992	Xq13.1	0.6355323	3.68928136	5.26545218	3.88E-05	0.00735663	2.33369991
ENSG00000213626.13_LBH	LBH	LBH regulator of WNT signaling pathway	HGNC:29532	2p23.1	-1.1139948	2.97217631	-5.2495621	4.02E-05	0.0075305	2.30068235
ENSG00000141428.17_C18orf21	C18orf21	chromosome 18 open reading frame 21	HGNC:28802	18q12.2	0.62463516	3.39789158	5.23754978	4.13E-05	0.00754929	2.27609695
ENSG00000147604.14_RPL7	RPL7	ribosomal protein L7	HGNC:10363	8q21.11	0.65851561	9.06153839	5.22269599	4.27E-05	0.00764703	2.23660093
ENSG00000165072.10_MAMDC2	MAMDC2	MAM domain containing 2	HGNC:23673	9q21.12	-1.5880112	2.03993458	-5.2408689	4.10E-05	0.00754929	2.2300503
ENSG00000187498.16_COL4A1	COL4A1	collagen type IV alpha 1 chain	HGNC:2202	13q34	-1.3210897	5.53138569	-5.2188663	4.31E-05	0.00764703	2.21933605
ENSG00000074181.9_NOTCH3	NOTCH3	notch receptor 3	HGNC:7883	19p13.12	-1.2326417	5.39445242	-5.2138029	4.36E-05	0.00764703	2.2085369
ENSG00000156453.13_PCDH1	PCDH1	protocadherin 1	HGNC:8655	5q31.3	-1.3834417	2.77425625	-5.2056148	4.44E-05	0.00764703	2.20354799
ENSG00000214736.7_TOMM6	TOMM6	translocase of outer mitochondrial membrane 6	HGNC:34528	6p21.1	0.72027769	5.49504036	5.20394381	4.45E-05	0.00764703	2.18647498
ENSG00000033327.13_GAB2	GAB2	GRB2 associated binding protein 2	HGNC:14458	11q14.1	-1.3327381	2.4161816	-5.195146	4.54E-05	0.00765689	2.1695041
ENSG00000234851.4_RPL23AP42	RPL23AP42	ribosomal protein L23a pseudogene 42	HGNC:35942	3q26.1	0.66903632	6.99365756	5.18253342	4.68E-05	0.00770593	2.14097694
ENSG00000135441.7_BLOC1S1	BLOC1S1	biogenesis of lysosomal organelles complex 1 subunit 1	HGNC:4200	12q13.2	0.66896053	5.87166119	5.18295417	4.67E-05	0.00770593	2.14071436
ENSG00000198034.11_RPS4X	RPS4X	ribosomal protein S4 X-linked	HGNC:10424	Xq13.1	0.62849474	8.57067103	5.16492309	4.87E-05	0.00791213	2.10947162
ENSG00000151779.13_NBAS	NBAS	NBAS subunit of NRZ tethering complex	HGNC:15625	2p24.3	0.66317258	4.91512617	5.16132059	4.91E-05	0.00791213	2.09624915
ENSG00000266472.5_MRPS21	MRPS21	mitochondrial ribosomal protein S21	HGNC:14046	1q21.2	0.72890976	4.47144404	5.14573034	5.08E-05	0.00811012	2.06735705
ENSG00000229117.9_RPL41	RPL41	ribosomal protein L41	HGNC:10354	12q13	0.78916596	8.77225106	5.12823372	5.29E-05	0.00811473	2.03139366
ENSG00000197345.13_MRPL21	MRPL21	mitochondrial ribosomal protein L21	HGNC:14479	11q13.3	0.72797053	4.89974495	5.12714613	5.30E-05	0.00811473	2.02285959
ENSG00000198369.10_SPRED2	SPRED2	sprouty related EVH1 domain containing 2	HGNC:17722	2p14	-1.175919	2.00495085	-5.1313282	5.25E-05	0.00811473	2.01792973
ENSG00000151693.11_ASAP2	ASAP2	ArfGAP with SH3 domain, ankyrin repeat and PH domain 2	HGNC:2721	2p25.1	-1.2617566	0.85427789	-5.2006977	4.49E-05	0.00764703	1.99012348
ENSG00000185010.15_F8	F8	coagulation factor VIII	HGNC:3546	Xq28	-0.6338659	3.51520307	-5.1010743	5.63E-05	0.00838108	1.98287804

ENSG00000095370.20_SH2D3C	SH2D3C	SH2 domain containing 3C	HGNC:16884	9q34.11	-1.0018935	2.80248356	-5.0963388	5.69E-05	0.00838108	1.97607254
ENSG00000187800.13_PEAR1	PEAR1	platelet endothelial aggregation receptor 1	HGNC:33631	1q23.1	-1.1076763	2.94265218	-5.0909561	5.76E-05	0.00838108	1.96584564
ENSG00000111639.8_MRPL51	MRPL51	mitochondrial ribosomal protein L51	HGNC:14044	12p13.31	0.62631238	6.18817237	5.08953452	5.78E-05	0.00838108	1.93815585
ENSG00000071082.11_RPL31	RPL31	ribosomal protein L31	HGNC:10334	2q11.2	0.76012906	8.35021779	5.08263812	5.87E-05	0.00838108	1.93013906
ENSG00000109475.16_RPL34	RPL34	ribosomal protein L34	HGNC:10340	4q25	0.76654026	7.68134233	5.08406208	5.85E-05	0.00838108	1.92970828
ENSG00000004399.12_PLXND1	PLXND1	plexin D1	HGNC:9107	3q22.1	-1.1516526	4.68346503	-5.0741562	5.98E-05	0.00846398	1.9101978
ENSG00000116251.11_RPL22	RPL22	ribosomal protein L22	HGNC:10315	1p36.31	0.62848185	8.16205984	5.06538101	6.10E-05	0.00855424	1.89155758
ENSG00000115268.9_RPS15	RPS15	ribosomal protein S15	HGNC:10388	19p13.3	0.62042456	8.38259691	5.05178416	6.30E-05	0.00866165	1.86344007
ENSG00000169976.6_SF3B5	SF3B5	splicing factor 3b subunit 5	HGNC:21083	6q24.2	0.6528869	5.14725934	5.05250051	6.29E-05	0.00866165	1.85907654
ENSG00000161281.11_COX7A1	COX7A1	cytochrome c oxidase subunit 7A1	HGNC:2287	19q13.12	0.94050127	9.28352478	5.02602659	6.68E-05	0.00892118	1.81366529
ENSG00000120279.6_MYCT1	MYCT1	MYC target 1	HGNC:23172	6q25.2	-0.7971138	2.2813761	-5.0206405	6.76E-05	0.00892118	1.80356816
ENSG00000170860.4_LSM3	LSM3	LSM3 homolog, U6 small nuclear RNA and mRNA degradation	HGNC:17874	3p25.1	0.67225514	5.45647065	5.02596994	6.68E-05	0.00892118	1.80020103
ENSG00000198242.14_RPL23A	RPL23A	ribosomal protein L23a	HGNC:10317	17q11.2	0.67513838	8.1532306	5.02220257	6.74E-05	0.00892118	1.79771426
ENSG00000168497.5_CAVIN2	CAVIN2	caveolae associated protein 2	HGNC:10690	2q32.3	-0.9693383	3.54725071	-5.0101411	6.92E-05	0.00902821	1.78626721
ENSG00000271811.1_Z97200.1	Z97200.1				1.8115049	1.17042364	5.13945273	5.16E-05	0.00811473	1.76844786
ENSG00000114391.13_RPL24	RPL24	ribosomal protein L24	HGNC:10325	3q12.3	0.70526451	8.17504303	5.00205694	7.05E-05	0.00911719	1.75407117
ENSG00000110700.7_RPS13	RPS13	ribosomal protein S13	HGNC:10386	11p15.1	0.71263293	8.53008475	4.99177926	7.22E-05	0.00924719	1.73426263
ENSG00000172586.8_CHCHD1	CHCHD1	coiled-coil-helix-coiled-coil-helix domain containing 1	HGNC:23518	10q22.2	0.71331772	4.45434499	4.98658965	7.31E-05	0.00924719	1.72250234
ENSG00000163682.16_RPL9	RPL9	ribosomal protein L9	HGNC:10369	4p14	0.89542073	8.29744465	4.9846882	7.34E-05	0.00924719	1.71703443
ENSG00000154721.15_JAM2	JAM2	junctional adhesion molecule 2	HGNC:14686	21q21.3	-0.9138442	2.64816977	-4.9708694	7.57E-05	0.00938628	1.71057287
ENSG00000147065.17_MSN	MSN	moesin	HGNC:7373	Xq12	-0.8817214	5.4454552	-4.9736223	7.53E-05	0.00938628	1.68619599
ENSG00000130021.13_PUDP	PUDP	pseudouridine 5'-phosphatase	HGNC:16818	Xp22.31	0.68744866	4.10308779	4.94773204	7.99E-05	0.00965136	1.64387621
ENSG00000197756.9_RPL37A	RPL37A	ribosomal protein L37a	HGNC:10348	2q35	0.77594003	9.80817079	4.94564634	8.02E-05	0.00965136	1.64330723
ENSG00000105258.9_POLR2I	POLR2I	RNA polymerase II subunit I	HGNC:9196	19q13.12	0.78365126	5.32422924	4.94574656	8.02E-05	0.00965136	1.62571938
ENSG00000285533.1_AP001362.2	AP001362.2				1.08228087	1.06101696	5.01918869	6.78E-05	0.00892118	1.61860778
ENSG00000131620.17_ANO1	ANO1	anoctamin 1	HGNC:21625	11q13.3	-1.0875432	2.41884578	-4.9286264	8.34E-05	0.0097835	1.61644121
ENSG00000156482.11_RPL30	RPL30	ribosomal protein L30	HGNC:10333	8q22.2	0.65769218	8.56310125	4.93678957	8.19E-05	0.00974491	1.61500307
ENSG00000112306.8_RPS12	RPS12	ribosomal protein S12	HGNC:10385	6q23.2	0.64270565	8.03722554	4.92818692	8.35E-05	0.0097835	1.59221873
ENSG00000163762.7_TM4SF18	TM4SF18	transmembrane 4 L six family member 18	HGNC:25181	3q25.1	-1.0016034	1.55301398	-4.9444835	8.05E-05	0.00965136	1.57891384
ENSG00000010319.6_SEMA3G	SEMA3G	semaphorin 3G	HGNC:30400	3p21.1	-1.1001993	3.81485289	-4.9089664	8.73E-05	0.00995093	1.56600948
ENSG00000090924.15_PLEKHG2	PLEKHG2	pleckstrin homology and RhoGEF domain containing G2	HGNC:29515	19q13.2	-1.1969359	2.24670343	-4.9040577	8.83E-05	0.00995093	1.55929661
ENSG00000091409.15_ITGA6	ITGA6	integrin subunit alpha 6	HGNC:6142	2q31.1	-0.7887251	4.05358481	-4.9067361	8.77E-05	0.00995093	1.5537983
ENSG00000142541.17_RPL13A	RPL13A	ribosomal protein L13a	HGNC:10304	19q13.33	0.62201375	10.174145	4.89558373	9.00E-05	0.00997869	1.53707431
ENSG00000152402.10_GUCY1A2	GUCY1A2	guanylate cyclase 1 soluble subunit alpha 2	HGNC:4684	11q22.3	-0.932075	1.53588874	-4.8990899	8.93E-05	0.00997869	1.50488686
ENSG00000154133.14_ROBO4	ROBO4	roundabout guidance receptor 4	HGNC:17985	11q24.2	-0.9932727	3.95530624	-4.8803137	9.32E-05	0.01020096	1.50095878
ENSG00000196369.11_SRGAP2B	SRGAP2B	SLIT-ROBO Rho GTPase activating protein 2B	HGNC:35237	1q21.1	-1.3710938	1.15610639	-4.9245203	8.42E-05	0.00978953	1.50068305
ENSG00000046889.19_PREX2	PREX2	phosphatidylinositol-3,4,5-trisphosphate dependent Rac exch	HGNC:22950	8q13.2	-1.0662681	1.49266653	-4.9046683	8.81E-05	0.00995093	1.49863167
ENSG00000205336.11_ADGRG1	ADGRG1	adhesion G protein-coupled receptor G1	HGNC:4512	16q21	-1.1824848	3.05193609	-4.8708373	9.52E-05	0.01024255	1.49781424
ENSG00000131831.18_RAI2	RAI2	retinoic acid induced 2	HGNC:9835	Xp22.13	-0.9769528	1.56784113	-4.8931134	9.05E-05	0.00997869	1.48781628
ENSG00000171421.13_MRPL36	MRPL36	mitochondrial ribosomal protein L36	HGNC:14490	5p15.33	0.6955854	4.33636499	4.87374855	9.46E-05	0.01024255	1.4782507
ENSG00000105722.10_ERF	ERF	ETS2 repressor factor	HGNC:3444	19q13.2	-1.0337699	2.29409242	-4.8620519	9.72E-05	0.01033575	1.47501602
ENSG00000211450.10_SELENOH	SELENOH	selenoprotein H	HGNC:18251	11q12.1	0.64579203	4.78184997	4.86909943	9.56E-05	0.01024255	1.46196172
ENSG00000151702.17_FLI1	FLI1	Fli-1 proto-oncogene, ETS transcription factor	HGNC:3749	11q24.3	-0.9274187	2.36566977	-4.8428291	0.00010157	0.01040395	1.43737082
ENSG00000134954.14_ETS1	ETS1	ETS proto-oncogene 1, transcription factor	HGNC:3488	11q24.3	-0.9313732	4.0177574	-4.8517338	9.95E-05	0.01033895	1.43435014
ENSG00000115350.11_POLE4	POLE4	DNA polymerase epsilon 4, accessory subunit	HGNC:18755	2p12	0.70206797	4.29309811	4.85168251	9.95E-05	0.01033895	1.43143053
ENSG00000165757.9_JCAD	JCAD	junctional cadherin 5 associated	HGNC:29283	10p11.23	-0.8530047	3.54287827	-4.8410384	0.00010199	0.01040395	1.42278127
ENSG00000255690.2_TRIL	TRIL	TLR4 interactor with leucine rich repeats	HGNC:22200	7p14.3	-1.4800999	1.80330752	-4.8565873	9.84E-05	0.01033895	1.41886795
ENSG00000125743.10_SNRPD2	SNRPD2	small nuclear ribonucleoprotein D2 polypeptide	HGNC:11159	19q13.2-q13.	0.72028884	5.37680337	4.84973218	1.00E-04	0.01033895	1.41532192
ENSG00000142534.7_RPS11	RPS11	ribosomal protein S11	HGNC:10384	19q13.3	0.63265879	9.2123038	4.82962508	0.0001047	0.01060818	1.387107
ENSG00000089057.15_SLC23A2	SLC23A2	solute carrier family 23 member 2	HGNC:10973	20p13	-0.868512	2.26650478	-4.8183766	0.00010744	0.01064844	1.38170966
ENSG00000130158.14_DOCK6	DOCK6	dedicator of cytokinesis 6	HGNC:19189	19p13.2	-1.1325331	3.99845727	-4.8254377	0.00010572	0.01063886	1.38030022
ENSG00000161649.13_CD300LG	CD300LG	CD300 molecule like family member g	HGNC:30455	17q21.31	-1.3314644	2.61329641	-4.8053279	0.00011072	0.01078044	1.36007133
ENSG00000107223.13_EDF1	EDF1	endothelial differentiation related factor 1	HGNC:3164	9q34.3	0.70670998	6.86868709	4.80713427	0.00011026	0.01078044	1.32230877
ENSG00000241468.7_ATP5MF	ATP5MF	ATP synthase membrane subunit f	HGNC:848	7q22.1	0.61485252	6.54358222	4.79857001	0.00011245	0.01085309	1.30286556
ENSG00000120318.16_ARAP3	ARAP3	ArfGAP with RhoGAP domain, ankyrin repeat and PH domain :	HGNC:24097	5q31.3	-0.8072574	3.13983217	-4.7791377	0.00011759	0.01085309	1.29760443
ENSG00000126217.21_MCF2L	MCF2L	MCF.2 cell line derived transforming sequence like	HGNC:14576	13q34	-1.0544425	4.03471649	-4.7842462	0.00011622	0.01085309	1.28835682
ENSG00000168894.9_RNF181	RNF181	ring finger protein 181	HGNC:28037	2p11.2	0.72768678	4.37875756	4.78436848	0.00011618	0.01085309	1.28329641
ENSG00000172809.13_RPL38	RPL38	ribosomal protein L38	HGNC:10349	17q25.1	0.76828113	8.20131803	4.78137278	0.00011699	0.01085309	1.27272713
ENSG00000267855.5_NDUFA7	NDUFA7	NADH:ubiquinone oxidoreductase subunit A7	HGNC:7691	19p13.2	0.64807947	6.21797644	4.78455494	0.00011614	0.01085309	1.27163575
ENSG00000115414.19_FN1	FN1	fibronectin 1	HGNC:3778	2q35	-1.2387414	5.6839988	-4.7818786	0.00011685	0.01085309	1.26654175
ENSG00000137726.17_FXYD6	FXYD6	FXYD domain containing ion transport regulator 6	HGNC:4030	11q23.3	-0.8298495	3.44136059	-4.7676463	0.00012074	0.01085309	1.26606647
ENSG00000139668.9_WDFY2	WDFY2	WD repeat and FYVE domain containing 2	HGNC:20482	13q14.3	-1.1049583	1.13471634	-4.8214121	0.0001067	0.01064844	1.26442139
ENSG00000240583.12_AQP1	AQP1	aquaporin 1 (Colton blood group)	HGNC:633	7p14.3	-0.9981583	6.31607183	-4.7773895	0.00011807	0.01085309	1.25691997
ENSG00000110442.12_COMMD9	COMMD9	COMM domain containing 9	HGNC:25014	11p13	0.51055486	4.70534887	4.77148505	0.00011968	0.01085309	1.248141
ENSG00000143947.13_RPS27A	RPS27A	ribosomal protein S27a	HGNC:10417	2p16.1	0.64590449	7.78907901	4.76718419	0.00012087	0.01085309	1.23847711
ENSG00000143436.11_MRPL9	MRPL9	mitochondrial ribosomal protein L9	HGNC:14277	1q21.3	0.51073139	4.90128582	4.76430797	0.00012167	0.01086015	1.23041261
ENSG00000213949.9_ITGA1	ITGA1	integrin subunit alpha 1	HGNC:6134	5q11.2	-0.7052148	3.38574048	-4.7494913	0.0001259	0.01097335	1.22788113
ENSG00000132530.17_XAF1	XAF1	XIAP associated factor 1	HGNC:30932	17p13.2	-1.408246	2.61839883	-4.7427016	0.00012788	0.01099507	1.22715946
ENSG00000113555.5_PCDH12	PCDH12	protocadherin 12	HGNC:8657	5q31.3	-1.1851055	2.03989234	-4.7471	0.00012659	0.01097335	1.22372458
ENSG00000167862.10_MRPL58	MRPL58	mitochondrial ribosomal protein L58	HGNC:5359	17q25.1	0.68579434	4.74359622	4.75814124	0.00012341	0.01095027	1.21954618
ENSG00000188186.10_LAMTOR4	LAMTOR4	late endosomal/lysosomal adaptor, MAPK and MTOR activato	HGNC:33772	7q22.1	0.53481707	4.63141266	4.75351558	0.00012474	0.01097335	1.2106117
ENSG00000165502.6_RPL36AL	RPL36AL	ribosomal protein L36a like	HGNC:10346	14q21.3	0.75614065	6.04271114	4.7470689	0.0001266	0.01097335	1.18918236
ENSG00000184014.8_DENND5A	DENND5A	DENN domain containing 5A	HGNC:19344	11p15.4	-0.5085335	4.36424513	-4.7399885	0.00012868	0.01099507	1.18323849
ENSG00000168509.20_HJV	HJV	hemojuvelin BMP co-receptor	HGNC:4887	1q21.1	0.67315139	8.41473194	4.73413651	0.00013043	0.01099507	1.17090728
ENSG00000087303.18_NID2	NID2	nidogen 2	HGNC:13389	14q22.1	-1.5170898	1.51798388	-4.7380381	0.00012926	0.01099507	1.16690066
ENSG00000117592.9_PRDX6	PRDX6	peroxiredoxin 6	HGNC:16753	1q25.1	0.52255933	7.95882232	4.73155915	0.00013121	0.01099507	1.16152038
ENSG00000102024.18_PLS3	PLS3	plastin 3	HGNC:9091	Xq23	-0.6762343	4.46249674	-4.7305822	0.0001315	0.01099507	1.16071886
ENSG00000182774.12_RPS17	RPS17	ribosomal protein S17	HGNC:10397	15q25.2	0.71234178	7.64716777	4.72899414	0.00013199	0.01099507	1.15391046
ENSG00000145425.10_RPS3A	RPS3A	ribosomal protein S3A	HGNC:10421	4q31.3	0.59024917	9.04627487	4.72296498	0.00013383	0.01099871	1.1526406
ENSG00000114541.15_FRMD4B	FRMD4B	FERM domain containing 4B	HGNC:24886	3p14.1	-1.4350692	0.63930002	-4.8163728	0.00010794	0.01064844	1.14755165
ENSG00000166441.13_RPL27A	RPL27A	ribosomal protein L27a	HGNC:10329	11p15.4	0.60098104	8.6473608	4.72199742	0.00013413	0.01099871	1.14648242
ENSG00000109089.7_CDR2L	CDR2L	cerebellar degeneration related protein 2 like	HGNC:29999	17q25.1	-1.2186487	1.00393301	-4.7695096	0.00012023	0.01085309	1.13693225
ENSG00000153885.14_KCTD15	KCTD15	potassium channel tetramerization domain containing 15	HGNC:23297	19q13.11	-1.4292691	1.52197861	-4.7216784	0.00013423	0.01099871	1.13616617
ENSG00000157570.11_TSPAN18	TSPAN18	tetraspanin 18	HGNC:20660	11p11.2	-1.1486913	3.03213361	-4.6799262	0.0001478	0.01166427	1.08725928
ENSG00000141552.17_ANAPC11	ANAPC11	anaphase promoting complex subunit 11	HGNC:14452	17q25.3	0.54654325	6.28233449	4.69839545	0.00014163	0.01154221	1.08224848
ENSG00000185650.9_ZFP36L1	ZFP36L1	ZFP36 ring finger protein like 1	HGNC:1107	14q24.1	-0.9702819	4.59170373	-4.6896072	0.00014453	0.01166427	1.07301218
ENSG00000229847.8_EMX2OS	EMX2OS	EMX2 opposite strand/antisense RNA	HGNC:18511	10q26.11	-1.0747013	1.47170442	-4.6873758	0.00014528	0.01166427	1.06749553
ENSG00000115970.18_THADA	THADA	THADA armadillo repeat containing	HGNC:19217	2p21	0.60098812	2.81993404	4.66787658	0.00015196	0.01183486	1.06641044
ENSG00000159640.16_ACE	ACE	angiotensin I converting enzyme	HGNC:2707	17q23.3	-1.7509982	3.06975466	-4.6646082	0.00015312	0.01183486	1.0578527
ENSG00000204262.13_COL5A2	COL5A2	collagen type V alpha 2 chain	HGNC:2210	2q32.2	-1.229168	3.29602036	-4.6683333	0.00015184	0.01183486	1.0571986
ENSG00000146122.17_DAAM2	DAAM2	dishevelled associated activator of morphogenesis 2	HGNC:18143	6p21.2	-1.2193002	4.82166678	-4.6843589	0.00014629	0.01166427	1.05662524
ENSG00000177954.13_RPS27	RPS27	ribosomal protein S27	HGNC:10416	1q21.3	0.74647413	8.81204789	4.66491403	0.00015301	0.01183486	1.02314944
ENSG00000125846.15_ZNF133	ZNF133	zinc finger protein 133	HGNC:12917	20p11.23	0.45864147	3.46896926	4.65405649	0.00015689	0.01199725	1.02146928
ENSG00000134247.10_PTGFRN	PTGFRN	prostaglandin F2 receptor inhibitor	HGNC:9601	1p13.1	-1.1817245	2.25345994	-4.6434452	0.00016078	0.01199725	1.01421038
ENSG00000114450.10_GNB4	GNB4	G protein subunit beta 4	HGNC:20731	3q26.33	-0.8919704	1.3642761	-4.6537988	0.00015698	0.01199725	0.98791281
ENSG00000118181.11_RPS25	RPS25	ribosomal protein S25	HGNC:10413	11q23.3	0.64732061	8.94390767	4.6441362	0.00016053	0.01199725	0.97915303
ENSG00000089009.15_RPL6	RPL6	ribosomal protein L6	HGNC:10362	12q24.13	0.53479342	8.71115237	4.64335439	0.00016082	0.01199725	0.97489838
ENSG00000177791.11_MYOZ1	MYOZ1	myozenin 1	HGNC:13752	10q22.2	0.56884325	11.4984108	4.62895745	0.00016626	0.01216095	0.96633567
ENSG00000140853.15_NLRC5	NLRC5	NLR family CARD domain containing 5	HGNC:29933	16q13	-1.1773097	2.68026319	-4.6182159	0.00017043	0.01226294	0.96331869
ENSG00000125977.7_EIF2S2	EIF2S2	eukaryotic translation initiation factor 2 subunit beta	HGNC:3266	20q11.22	0.5215784	6.30690553	4.63934298	0.00016231	0.01204898	0.95231173
ENSG00000151239.13_TWF1	TWF1	twinfilin actin binding protein 1	HGNC:9620	12q12	-0.7953314	1.94742692	-4.6162139	0.00017122	0.01226294	0.94945739

ENSG00000163157.15_TMOD4	TMOD4	tropomodulin 4	HGNC:11874	1q21.3	0.60658463	8.71407158	4.631551	0.00016526	0.01216095	0.94892834
ENSG00000116586.11_LAMTOR2	LAMTOR2	late endosomal/lysosomal adaptor, MAPK and MTOR activato	HGNC:29796	1q22	0.54847889	4.57380646	4.62989321	0.0001659	0.01216095	0.94014214
ENSG00000100410.8_PHF5A	PHF5A	PHD finger protein 5A	HGNC:18000	22q13.2	0.63303656	3.14146115	4.60492785	0.00017575	0.01236366	0.92466686
ENSG00000162244.11_RPL29	RPL29	ribosomal protein L29	HGNC:10331	3p21.2	0.73314222	9.18052407	4.61720117	0.00017083	0.01226294	0.92213912
ENSG00000008988.9_RPS20	RPS20	ribosomal protein S20	HGNC:10405	8q12.1	0.64092681	8.56538317	4.61249839	0.0001727	0.01226294	0.9054262
ENSG00000151500.15_THYN1	THYN1	thymocyte nuclear protein 1	HGNC:29560	11q25	0.51008872	3.32827672	4.59829934	0.00017846	0.01244677	0.9053082
ENSG00000103647.12_CORO2B	CORO2B	coronin 2B	HGNC:2256	15q23	-1.2685662	0.91157344	-4.6481974	0.00015903	0.01199725	0.90012423
ENSG00000198830.11_HMGN2	HMGN2	high mobility group nucleosomal binding domain 2	HGNC:4986	1p36.11	0.68533379	6.71751383	4.61364001	0.00017225	0.01226294	0.89630933
ENSG00000169994.18_MYO7B	MYO7B	myosin VIIB	HGNC:7607	2q14.3	-1.3087258	1.17851861	-4.6176848	0.00017064	0.01226294	0.88652827
ENSG00000105971.15_CAV2	CAV2	caveolin 2	HGNC:1528	7q31.2	-0.8591487	3.38583512	-4.5914862	0.0001813	0.01249389	0.88312669
ENSG00000142676.14_RPL11	RPL11	ribosomal protein L11	HGNC:10301	1p36.11	0.60235701	7.98594028	4.60321392	0.00017645	0.01236366	0.87943147
ENSG00000007237.18_GAS7	GAS7	growth arrest specific 7	HGNC:4169	17p13.1	-1.2765342	3.64756774	-4.5860363	0.0001836	0.01251377	0.86806009
ENSG00000210140.1_MT-TC	MT-TC	mitochondrially encoded tRNA-Cys (UGU/C)	HGNC:7477	mitochondria	1.79269065	-0.615243	5.11876897	5.41E-05	0.00818726	0.86676555
ENSG00000137033.11_IL33	IL33	interleukin 33	HGNC:16028	9p24.1	-1.114256	1.67860731	-4.5800523	0.00018616	0.01263075	0.86533906
ENSG00000172590.18_MRPL52	MRPL52	mitochondrial ribosomal protein L52	HGNC:16655	14q11.2	0.73280666	4.44103251	4.59067255	0.00018164	0.01249389	0.85716144
ENSG00000173376.14_NDNF	NDNF	neuron derived neurotrophic factor	HGNC:26256	4q27	-1.4079338	0.29890225	-4.6853994	0.00014594	0.01166427	0.85583492
ENSG00000185608.8_MRPL40	MRPL40	mitochondrial ribosomal protein L40	HGNC:14491	22q11.21	0.50647103	5.26539316	4.59449825	0.00018004	0.01249389	0.85431172
ENSG00000055813.6_CCDC85A	CCDC85A	coiled-coil domain containing 85A	HGNC:29400	2p16.1	-1.8139617	0.49236114	-4.6811598	0.00014738	0.01166427	0.85259427
ENSG00000205730.6_ITPRIPL2	ITPRIPL2	ITPRIP like 2	HGNC:27257	16p12.3	-0.6627647	3.45695451	-4.5735879	0.00018896	0.01270606	0.84345624
ENSG00000150456.10_EEF1AKMT1	EEF1AKMT1	EEF1A lysine methyltransferase 1	HGNC:27351	13q12.11	0.85144751	1.85386946	4.5687304	0.0001911	0.01274056	0.8413584
ENSG00000131697.18_NPHP4	NPHP4	nephrocystin 4	HGNC:19104	1p36.31	-1.260784	0.97483785	-4.6108143	0.00017337	0.01226294	0.82292087
ENSG00000113558.18_SKP1	SKP1	S-phase kinase associated protein 1	HGNC:10899	5q31.1	0.54170347	7.74353869	4.57679185	0.00018756	0.01266905	0.81969324
ENSG00000003400.14_CASP10	CASP10	caspase 10	HGNC:1500	2q33.1	-0.7433148	2.18669203	-4.5479011	0.00020053	0.01285034	0.81261376
ENSG00000101695.9_RNF125	RNF125	ring finger protein 125	HGNC:21150	18q12.1	-1.3641113	1.71863601	-4.5519494	0.00019866	0.01278513	0.80362236
ENSG00000100626.17_GALNT16	GALNT16	polypeptide N-acetylgalactosaminyltransferase 16	HGNC:23233	14q24.1	-1.2941675	2.79245281	-4.5435863	0.00020255	0.0128624	0.80192601
ENSG00000105193.9_RPS16	RPS16	ribosomal protein S16	HGNC:10396	19q13.2	0.76966004	8.91402013	4.5609408	0.00019457	0.01274056	0.79582911
ENSG00000106400.12_ZNHIT1	ZNHIT1	zinc finger HIT-type containing 1	HGNC:21688	7q22.1	0.57736405	5.30409174	4.56626761	0.00019219	0.01274056	0.79190665
ENSG00000182899.16_RPL35A	RPL35A	ribosomal protein L35a	HGNC:10345	3q29	0.66271262	7.70925684	4.56263199	0.00019381	0.01274056	0.78814041
ENSG00000243927.6_MRPS6	MRPS6	mitochondrial ribosomal protein S6	HGNC:14051	21q22.11	0.68767231	4.90344228	4.56207961	0.00019406	0.01274056	0.78543912
ENSG00000173141.5_MRPL57	MRPL57	mitochondrial ribosomal protein L57	HGNC:14514	13q12.11	0.55721149	5.2838812	4.56302846	0.00019363	0.01274056	0.78494201
ENSG00000152952.12_PLOD2	PLOD2	procollagen-lysine,2-oxoglutarate 5-dioxygenase 2	HGNC:9082	3q24	-1.111389	1.21343984	-4.5569031	0.0001964	0.01278513	0.77300794
ENSG00000173113.6_TRMT112	TRMT112	tRNA methyltransferase subunit 11-2	HGNC:26940	11q13.1	0.65248886	5.51179015	4.55566884	0.00019696	0.01278513	0.76780008
ENSG00000134369.15_NAV1	NAV1	neuron navigator 1	HGNC:15989	1q32.1	-0.8916485	3.10562324	-4.5329751	0.00020758	0.0128624	0.76718228
ENSG00000205323.9_SARNP	SARNP	SAP domain containing ribonucleoprotein	HGNC:24432	12q13.2	0.59653632	4.91397985	4.55245009	0.00019843	0.01278513	0.76483218
ENSG00000117543.21_DPH5	DPH5	diphthamide biosynthesis 5	HGNC:24270	1p21.2	0.67867556	3.10446502	4.5305735	0.00020874	0.01287525	0.76478554
ENSG00000149639.15_SOGA1	SOGA1	suppressor of glucose, autophagy associated 1	HGNC:16111	20q11.23	-1.0232746	3.17910054	-4.5230236	0.00021242	0.01287525	0.74626841
ENSG00000131469.14_RPL27	RPL27	ribosomal protein L27	HGNC:10328	17q21.31	0.6720696	8.58269577	4.5385641	0.00020491	0.0128624	0.74297256
ENSG00000198755.11_RPL10A	RPL10A	ribosomal protein L10a	HGNC:10299	6p21.31	0.62670279	8.12718612	4.5404396	0.00020403	0.0128624	0.74241461
ENSG00000146007.10_ZMAT2	ZMAT2	zinc finger matrin-type 2	HGNC:26433	5q31.3	0.4649928	5.45212964	4.53734752	0.00020549	0.0128624	0.72726626
ENSG00000149806.11_FAU	FAU	FAU ubiquitin like and ribosomal protein S30 fusion	HGNC:3597	11q13.1	0.59347906	7.61212925	4.53351677	0.00020732	0.0128624	0.72326131
ENSG00000108298.11_RPL19	RPL19	ribosomal protein L19	HGNC:10312	17q12	0.59426019	8.93554711	4.52680961	0.00021057	0.01287525	0.7213667
ENSG00000162384.14_CZIB	CZIB	CXXC motif containing zinc binding protein	HGNC:26059	1p32.3	0.56300495	5.45801211	4.5340752	0.00020705	0.0128624	0.72018766
ENSG00000221818.9_EBF2	EBF2	EBF transcription factor 2	HGNC:19090	8p21.2	-1.0535398	2.07904222	-4.5020037	0.00022302	0.01327059	0.71567206
ENSG00000134419.15_RPS15A	RPS15A	ribosomal protein S15a	HGNC:10389	16p12.3	0.65082783	8.53862843	4.52256235	0.00021265	0.01287525	0.70727902
ENSG00000154529.14_CNTNAP3B	CNTNAP3B	contactin associated protein family member 3B	HGNC:32035	9p11.2	-1.8920731	0.42965886	-4.647301	0.00015936	0.01199725	0.69990955
ENSG00000185760.15_KCNQ5	KCNQ5	potassium voltage-gated channel subfamily Q member 5	HGNC:6299	6q13	1.23240437	5.11357136	4.52196762	0.00021294	0.01287525	0.6985182
ENSG00000084623.11_EIF3I	EIF3I	eukaryotic translation initiation factor 3 subunit I	HGNC:3272	1p35.2	0.57287682	6.3370822	4.52325698	0.00021231	0.01287525	0.69613215
ENSG00000273749.5_CYFIP1	CYFIP1	cytoplasmic FMR1 interacting protein 1	HGNC:13759	15q11.2	-0.5603634	4.12574231	-4.5089937	0.00021944	0.01310945	0.68155667
ENSG00000131495.8_NDUFA2	NDUFA2	NADH:ubiquinone oxidoreductase subunit A2	HGNC:7685	5q31.3	0.71801803	6.3626251	4.51535212	0.00021623	0.01302154	0.67869019
ENSG00000154783.11_FGD5	FGD5	FYVE, RhoGEF and PH domain containing 5	HGNC:19117	3p25.1	-1.1111535	2.77436942	-4.4868758	0.00023098	0.01342462	0.67764194
ENSG00000118263.15_KLF7	KLF7	Kruppel like factor 7	HGNC:6350	2q33.3	-0.8256815	2.11175169	-4.4835368	0.00023277	0.01342462	0.67697218
ENSG00000133112.16_TPT1	TPT1	tumor protein, translationally-controlled 1	HGNC:12022	13q14.13	0.61189236	12.5000009	4.49134329	0.0002286	0.01342462	0.67414629
ENSG00000186010.19_NDUFA13	NDUFA13	NADH:ubiquinone oxidoreductase subunit A13	HGNC:17194	19p13.11	0.69299824	7.65627585	4.51087554	0.00021848	0.01310463	0.67358432
ENSG00000106605.11_BLVRA	BLVRA	biliverdin reductase A	HGNC:1062	7p13	0.55859456	4.33507235	4.49047608	0.00022906	0.01342462	0.63720472
ENSG00000186468.13_RPS23	RPS23	ribosomal protein S23	HGNC:10410	5q14.2	0.6434127	8.81253437	4.48515914	0.0002319	0.01342462	0.62847899
ENSG00000124172.10_ATP5F1E	ATP5F1E	ATP synthase F1 subunit epsilon	HGNC:838	20q13.32	0.74583274	6.98534474	4.48970153	0.00022947	0.01342462	0.62361802
ENSG00000147677.11_EIF3H	EIF3H	eukaryotic translation initiation factor 3 subunit H	HGNC:3273	8q23.3-q24.1	0.60375437	6.84574103	4.48973824	0.00022945	0.01342462	0.62332927
ENSG00000161970.14_RPL26	RPL26	ribosomal protein L26	HGNC:10327	17p13.1	0.62723723	8.08959698	4.4840949	0.00023247	0.01342462	0.61818029
ENSG00000126785.13_RHOJ	RHOJ	ras homolog family member J	HGNC:688	14q23.2	-0.7867093	1.94020778	-4.452772	0.00024998	0.01409187	0.61150584
ENSG00000104147.9_OIP5	OIP5	Opa interacting protein 5	HGNC:20300	15q15.1	1.07320192	1.8253737	4.46098209	0.00024527	0.01393088	0.6108181
ENSG00000130584.11_ZBTB46	ZBTB46	zinc finger and BTB domain containing 46	HGNC:16094	20q13.33	-0.797933	2.43564169	-4.4475961	0.000253	0.01417053	0.60141928
ENSG00000120278.16_PLEKHG1	PLEKHG1	pleckstrin homology and RhoGEF domain containing G1	HGNC:20884	6q25.1	-1.2123817	1.01510922	-4.4798247	0.00023478	0.01348874	0.60109745
ENSG00000170889.14_RPS9	RPS9	ribosomal protein S9	HGNC:10442	19q13.42	0.60029323	8.60634746	4.46861201	0.00024097	0.01373866	0.58932992
ENSG00000134824.14_FADS2	FADS2	fatty acid desaturase 2	HGNC:3575	11q12.2	-1.2366501	2.52671604	-4.4410482	0.00025687	0.01421312	0.58606771
ENSG00000198873.11_GRK5	GRK5	G protein-coupled receptor kinase 5	HGNC:4544	10q26.11	-0.838556	3.03559203	-4.4471419	0.00025326	0.01417053	0.58282806
ENSG00000235316.1_DUSP8P5	DUSP8P5	dual specificity phosphatase 8 pseudogene 5	HGNC:45003	10q22.2	-1.2068819	0.28935383	-4.58763	0.00018292	0.01251377	0.57730374
ENSG00000171056.8_SOX7	SOX7	SRY-box transcription factor 7	HGNC:18196	8p23.1	-0.8966358	2.65969757	-4.4320947	0.00026226	0.01428077	0.56438298
ENSG00000145592.14_RPL37	RPL37	ribosomal protein L37	HGNC:10347	5p13.1	0.68923406	8.16659485	4.45902211	0.00024638	0.01394152	0.56343675
ENSG00000107798.18_LIPA	LIPA	lipase A, lysosomal acid type	HGNC:6617	10q23.31	-0.6580675	3.1831711	-4.4335271	0.00026139	0.01428077	0.54871717
ENSG00000163909.8_HEYL	HEYL	hes related family bHLH transcription factor with YRPW motif	HGNC:4882	1p34.2	-0.9201453	1.82870715	-4.4233651	0.00026763	0.0144354	0.54658241
ENSG00000142937.12_RPS8	RPS8	ribosomal protein S8	HGNC:10441	1p34.1	0.54148908	8.75395855	4.4432828	0.00025554	0.01419201	0.5356052
ENSG00000083845.9_RPS5	RPS5	ribosomal protein S5	HGNC:10426	19q13.43	0.65206799	8.28038808	4.44438229	0.00025489	0.01419201	0.53214046
ENSG00000147526.20_TACC1	TACC1	transforming acidic coiled-coil containing protein 1	HGNC:11522	8p11.22	-0.7134226	4.35249636	-4.4373183	0.0002591	0.01423159	0.51729073
ENSG00000128918.15_ALDH1A2	ALDH1A2	aldehyde dehydrogenase 1 family member A2	HGNC:15472	15q21.3	-1.3568785	2.38411965	-4.4002232	0.00028239	0.01501574	0.50131623
ENSG00000047249.18_ATP6V1H	ATP6V1H	ATPase H+ transporting V1 subunit H	HGNC:18303	8q11.23	0.43039338	4.45438946	4.43032606	0.00026334	0.01428077	0.50092149
ENSG00000088367.22_EPB41L1	EPB41L1	erythrocyte membrane protein band 4.1 like 1	HGNC:3378	20q11.23	-0.9953595	2.80485228	-4.4004078	0.00028227	0.01501574	0.49044198
ENSG00000169599.12_NFU1	NFU1	NFU1 iron-sulfur cluster scaffold	HGNC:16287	2p13.3	0.58579692	5.25974295	4.42956046	0.00026381	0.01428077	0.48991949
ENSG00000171680.21_PLEKHG5	PLEKHG5	pleckstrin homology and RhoGEF domain containing G5	HGNC:29105	1p36.31	-1.4362872	2.63092693	-4.386666	0.00029142	0.01533267	0.46883892
ENSG00000156535.15_CD109	CD109	CD109 molecule	HGNC:21685	6q13	-1.015661	0.68193284	-4.4374291	0.00025904	0.01423159	0.46224153
ENSG00000112139.16_MDGA1	MDGA1	MAM domain containing glycosylphosphatidylinositol anchor	HGNC:19267	6p21.2	-1.5661999	0.5155895	-4.4699546	0.00024022	0.01373866	0.45174544
ENSG00000010318.21_PHF7	PHF7	PHD finger protein 7	HGNC:18458	3p21.1	-0.8021465	0.98222218	-4.4138495	0.0002736	0.01470477	0.44687878
ENSG00000103034.14_NDRG4	NDRG4	NDRG family member 4	HGNC:14466	16q21	-1.4146991	1.53491232	-4.3792131	0.0002965	0.0153758	0.44545668
ENSG00000005075.15_POLR2J	POLR2J	RNA polymerase II subunit J	HGNC:9197	7q22.1	0.60656929	5.67295611	4.4066728	0.0002782	0.01489831	0.43806246
ENSG00000111885.7_MAN1A1	MAN1A1	mannosidase alpha class 1A member 1	HGNC:6821	6q22.31	-1.2629254	2.54927557	-4.3701775	0.00030279	0.01560253	0.43260286
ENSG00000276445.1_AC005393.1	AC005393.1				1.05342986	2.20904032	4.3674048	0.00030475	0.01564953	0.43231312
ENSG00000102755.12_FLT1	FLT1	fms related receptor tyrosine kinase 1	HGNC:3763	13q12.3	-0.6840573	3.63571426	-4.3893143	0.00028963	0.01529235	0.43055894
ENSG00000241343.9_RPL36A	RPL36A	ribosomal protein L36a	HGNC:10359	Xq22.1	0.6973115	7.32194478	4.3982295	0.0002837	0.01503208	0.42287194
ENSG00000182957.16_SPATA13	SPATA13	spermatogenesis associated 13	HGNC:23222	13q12.12	-0.885762	1.54327607	-4.3611882	0.00030918	0.01576764	0.40836055
ENSG00000112175.8_BMP5	BMP5	bone morphogenetic protein 5	HGNC:1072	6p12.1	-0.8794515	1.29003575	-4.3652093	0.0003063	0.01567583	0.39939545
ENSG00000168653.11_NDUFS5	NDUFS5	NADH:ubiquinone oxidoreductase subunit S5	HGNC:7712	1p34.3	0.77791299	7.24785128	4.38462606	0.0002928	0.01535158	0.39240959
ENSG00000177600.9_RPLP2	RPLP2	ribosomal protein lateral stalk subunit P2	HGNC:10377	11p15.5	0.70078371	8.17093557	4.38063064	0.00029553	0.0153758	0.39052415
ENSG00000263165.1_AC087292.1	AC087292.1				1.0174703	1.03386529	4.38090702	0.00029534	0.0153758	0.3852668
ENSG00000105393.16_BABAM1	BABAM1	BRISC and BRCA1 A complex member 1	HGNC:25008	19p13.11	0.53724727	5.90265821	4.37796465	0.00029737	0.0153758	0.37438756
ENSG00000092421.16_SEMA6A	SEMA6A	semaphorin 6A	HGNC:10738	5q23.1	-0.8856456	2.3409493	-4.3338311	0.00032946	0.0161808	0.36001112
ENSG00000086062.12_B4GALT1	B4GALT1	beta-1,4-galactosyltransferase 1	HGNC:924	9p21.1	-0.7158098	4.10493608	-4.3597651	0.0003102	0.01576764	0.35631145
ENSG00000159433.12_STARD9	STARD9	StAR related lipid transfer domain containing 9	HGNC:19162	15q15.2	-0.9710089	3.19009428	-4.343855	0.00032188	0.01614231	0.35593941
ENSG00000173894.11_CBX2	CBX2	chromobox 2	HGNC:1552	17q25.3	-1.5193943	-0.1993246	-4.5398463	0.00020431	0.0128624	0.35281033
ENSG00000174444.15_RPL4	RPL4	ribosomal protein L4	HGNC:10353	15q22.31	0.48766723	9.70454364	4.35315903	0.000315	0.01590359	0.3490107
ENSG00000176095.12_IP6K1	IP6K1	inositol hexakisphosphate kinase 1	HGNC:18360	3p21.31	-0.5468435	3.64891803	-4.3485689	0.00031837	0.01602011	0.34704214
ENSG00000187678.9_SPRY4	SPRY4	sprouty RTK signaling antagonist 4	HGNC:15533	5q31.3	-0.9827823	1.79116677	-4.3258602	0.00033562	0.01633958	0.3441643
ENSG00000153071.15_DAB2	DAB2	DAB adaptor protein 2	HGNC:2662	5p13.1	-1.1424446	3.19515688	-4.3345463	0.00032891	0.0161808	0.3346115

ENSG00000198435.4_NRARP	NRARP	NOTCH regulated ankyrin repeat protein	HGNC:33843	9q34.3	-1.0380492	1.31107554	-4.3308062	0.00033178	0.01620552	0.33175909
ENSG00000147573.17_TRIM55	TRIM55	tripartite motif containing 55	HGNC:14215	8q13.1	0.64518224	4.76082868	4.35526279	0.00031346	0.01587955	0.33121963
ENSG00000244187.8_TMEM141	TMEM141	transmembrane protein 141	HGNC:28211	9q34.3	0.70611918	3.56758494	4.33650099	0.00032742	0.0161808	0.32877237
ENSG00000151474.23_FRMD4A	FRMD4A	FERM domain containing 4A	HGNC:25491	10p13	-1.0746789	2.26353221	-4.3122934	0.00034637	0.01652688	0.31594876
ENSG00000120656.11_TAF12	TAF12	TATA-box binding protein associated factor 12	HGNC:11545	1p35.3	0.65557711	3.06702174	4.3208802	0.00033952	0.01644922	0.3135353
ENSG00000147408.14_CSGALNACT1	CSGALNACT1	chondroitin sulfate N-acetylgalactosaminyltransferase 1	HGNC:24290	8p21.3	-1.0719281	1.27321453	-4.3201961	0.00034006	0.01644922	0.30629682
ENSG00000128052.9_KDR	KDR	kinase insert domain receptor	HGNC:6307	4q12	-0.9818847	2.49562932	-4.3066059	0.00035097	0.01652688	0.29998831
ENSG00000148303.17_RPL7A	RPL7A	ribosomal protein L7a	HGNC:10364	9q34.2	0.48331387	8.96943186	4.3342774	0.00032912	0.0161808	0.29831681
ENSG00000101445.10_PPP1R16B	PPP1R16B	protein phosphatase 1 regulatory subunit 16B	HGNC:15850	20q11.23	-1.30791	2.09313944	-4.3017761	0.00035494	0.01652688	0.29522887
ENSG00000111452.13_ADGRD1	ADGRD1	adhesion G protein-coupled receptor D1	HGNC:19893	12q24.33	-1.2598737	2.40901538	-4.2997152	0.00035664	0.01652688	0.28924187
ENSG00000139679.15_LPAR6	LPAR6	lysophosphatidic acid receptor 6	HGNC:15520	13q14.2	-0.7548395	1.93213284	-4.2991608	0.0003571	0.01652688	0.28873027
ENSG00000104689.9_TNFRSF10A	TNFRSF10A	TNF receptor superfamily member 10a	HGNC:11904	8p21.3	-2.0020627	-0.9537624	-4.790264	0.00011462	0.01085309	0.28735707
ENSG00000153904.20_DDAH1	DDAH1	dimethylarginine dimethylaminohydrolase 1	HGNC:2715	1p22.3	0.57076853	5.22875213	4.33449303	0.00032895	0.0161808	0.27994837
ENSG00000109084.14_TMEM97	TMEM97	transmembrane protein 97	HGNC:28106	17q11.2	0.79018496	2.45121863	4.29527119	0.00036034	0.01660329	0.27906402
ENSG00000125971.16_DYNLRB1	DYNLRB1	dynein light chain roadblock-type 1	HGNC:15468	20q11.22	0.49383125	5.46498513	4.33286782	0.0003302	0.0161808	0.27501579
ENSG00000131634.14_TMEM204	TMEM204	transmembrane protein 204	HGNC:14158	16p13.3	-0.6800601	3.02933642	-4.3009726	0.0003556	0.01652688	0.26432756
ENSG00000227671.4_AL390728.4	AL390728.4				-1.100099	0.6232127	-4.3357721	0.00032798	0.0161808	0.26299237
ENSG00000108175.17_ZMIZ1	ZMIZ1	zinc finger MIZ-type containing 1	HGNC:16493	10q22.3	-0.8185506	4.36072785	-4.3180699	0.00034175	0.01647753	0.25567661
ENSG00000172716.16_SLFN11	SLFN11	schlafen family member 11	HGNC:26633	17q12	-0.7436305	2.21789301	-4.278263	0.00037487	0.01701414	0.24401701
ENSG00000152583.12_SPARCL1	SPARCL1	SPARC like 1	HGNC:11220	4q22.1	-0.607121	6.83576346	-4.3145154	0.00034458	0.01652688	0.23672203
ENSG00000066735.14_KIF26A	KIF26A	kinesin family member 26A	HGNC:20226	14q32.33	-1.349662	2.14846644	-4.2734871	0.00037906	0.01706027	0.23559125
ENSG00000160799.11_CCDC12	CCDC12	coiled-coil domain containing 12	HGNC:28332	3p21.31	0.65398246	4.42838641	4.30760691	0.00035016	0.01652688	0.23315177
ENSG00000160111.13_CPAMD8	CPAMD8	C3 and PZP like alpha-2-macroglobulin domain containing 8	HGNC:23228	19p13.11	-1.2869074	1.81274257	-4.2709425	0.00038131	0.01706027	0.23044703
ENSG00000111481.10_COPZ1	COPZ1	COPI coat complex subunit zeta 1	HGNC:2243	12q13.13	0.45519979	5.72134755	4.31266791	0.00034607	0.01652688	0.22984164
ENSG00000132963.8_POMP	POMP	proteasome maturation protein	HGNC:20330	13q12.3	0.58240466	6.0427084	4.31028453	0.00034799	0.01652688	0.22467306
ENSG00000236552.2_RPL13AP5	RPL13AP5	ribosomal protein L13a pseudogene 5	HGNC:23736	10q24.1	0.69483631	2.1601344	4.26716117	0.00038467	0.01706027	0.22250603
ENSG00000204822.7_MRPL53	MRPL53	mitochondrial ribosomal protein L53	HGNC:16684	2p13.1	0.6126103	5.11731829	4.30524096	0.00035209	0.01652688	0.2163769
ENSG00000134817.10_APLNR	APLNR	apelin receptor	HGNC:339	11q12.1	-1.0881279	3.72118849	-4.2869608	0.00036737	0.0167613	0.21186581
ENSG00000204291.11_COL15A1	COL15A1	collagen type XV alpha 1 chain	HGNC:2192	9q22.33	-1.3650702	5.17222701	-4.3014596	0.0003552	0.01652688	0.20948228
ENSG00000188846.13_RPL14	RPL14	ribosomal protein L14	HGNC:10305	3p22.1	0.58333283	8.48036282	4.29452815	0.00036097	0.01660329	0.20393582
ENSG00000139514.13_SLC7A1	SLC7A1	solute carrier family 7 member 1	HGNC:11057	13q12.3	-1.389991	0.84363348	-4.3052673	0.00035207	0.01652688	0.20178201
ENSG00000020181.17_ADGRA2	ADGRA2	adhesion G protein-coupled receptor A2	HGNC:17849	8p11.23	-1.5349038	3.10669918	-4.2666867	0.0003851	0.01706027	0.19400229
ENSG00000275832.5_ARHGAP23	ARHGAP23	Rho GTPase activating protein 23	HGNC:29293	17q12	-1.072273	3.52563627	-4.2667928	0.000385	0.01706027	0.17659573
ENSG00000070614.15_NDST1	NDST1	N-deacetylase and N-sulfotransferase 1	HGNC:7680	5q33.1	-0.6695501	5.11939551	-4.286512	0.00036775	0.0167613	0.17489037
ENSG00000042062.12_RIPOR3	RIPOR3	RIPOR family member 3	HGNC:16168	20q13.13	-0.88846	2.46875947	-4.2478051	0.00040238	0.01738819	0.17341649
ENSG00000109436.8_TBC1D9	TBC1D9	TBC1 domain family member 9	HGNC:21710	4q31.21	-1.050656	1.12970764	-4.2547823	0.00039591	0.01730789	0.15991911
ENSG00000105372.7_RPS19	RPS19	ribosomal protein S19	HGNC:10402	19q13.2	0.62027208	7.89203525	4.27746342	0.00037557	0.01701414	0.15976775
ENSG00000170310.15_STX8	STX8	syntaxin 8	HGNC:11443	17p13.1	0.62150983	3.78316251	4.26457846	0.00038699	0.01706027	0.15935904
ENSG00000151690.15_MFSD6	MFSD6	major facilitator superfamily domain containing 6	HGNC:24711	2q32.2	-1.1002797	1.39578556	-4.2435504	0.00040638	0.01751061	0.1592643
ENSG00000180447.7_GAS1	GAS1	growth arrest specific 1	HGNC:4165	9q21.33	-1.1514423	3.04012333	-4.248632	0.00040161	0.01738819	0.15684628
ENSG00000105640.13_RPL18A	RPL18A	ribosomal protein L18a	HGNC:10311	19p13.11	0.5641445	9.01148117	4.26763085	0.00038425	0.01706027	0.15157202
ENSG00000127954.12_STEAP4	STEAP4	STEAP4 metalloreductase	HGNC:21923	7q21.12	-0.6322691	3.74371563	-4.2634955	0.00038797	0.01706027	0.15089143
ENSG00000198838.13_RYR3	RYR3	ryanodine receptor 3	HGNC:10485	15q13.3-q14	1.46376521	5.44241695	4.27369496	0.00037888	0.01706027	0.14736784
ENSG00000065308.5_TRAM2	TRAM2	translocation associated membrane protein 2	HGNC:16855	6p12.2	-0.7882316	2.63893432	-4.2364642	0.00041313	0.01760869	0.14377827
ENSG00000135390.19_ATP5MC2	ATP5MC2	ATP synthase membrane subunit c locus 2	HGNC:842	12q13.13	0.54752033	8.0205801	4.26586733	0.00038583	0.01706027	0.13545261
ENSG00000253276.3_CCDC71L	CCDC71L	coiled-coil domain containing 71 like	HGNC:26685	7q22.3	-0.8923726	1.4055809	-4.224472	0.00042482	0.01784352	0.11830809
ENSG00000164032.12_H2AFZ	H2AFZ	H2A.Z variant histone 1	HGNC:4741	4q23	0.57378539	5.10085104	4.25774863	0.00039319	0.01723919	0.11103975
ENSG00000128917.8_DLL4	DLL4	delta like canonical Notch ligand 4	HGNC:2910	15q15.1	-1.0606729	1.7706217	-4.2147201	0.00043456	0.01794675	0.10990848
ENSG00000061918.13_GUCY1B1	GUCY1B1	guanylate cyclase 1 soluble subunit beta 1	HGNC:4687	4q32.1	-0.8796797	1.52313609	-4.2098407	0.00043952	0.01794675	0.09682198
ENSG00000130508.11_PXDN	PXDN	peroxidasin	HGNC:14966	2p25.3	-1.2228701	3.20880171	-4.2255005	0.0004238	0.01784352	0.09534636
ENSG00000139567.12_ACVRL1	ACVRL1	activin A receptor like type 1	HGNC:175	12q13.13	-0.7016726	3.86871725	-4.2368427	0.00041277	0.01760869	0.09236769
ENSG00000150316.12_CWC15	CWC15	CWC15 spliceosome associated protein homolog	HGNC:26939	11q21	0.58408902	5.63313081	4.24818451	0.00040203	0.01738819	0.08728158
ENSG00000104728.16_ARHGEF10	ARHGEF10	Rho guanine nucleotide exchange factor 10	HGNC:14103	8p23.3	-0.8713322	2.34376989	-4.2056941	0.00044378	0.01794675	0.08553554
ENSG00000260442.5_ATP2A1-AS1	ATP2A1-AS1	ATP2A1 antisense RNA 1	HGNC:51370	16p11.2	0.9271868	1.85871498	4.20261628	0.00044697	0.01794675	0.08415974
ENSG00000136810.13_TXN	TXN	thioredoxin	HGNC:12435	9q31.3	0.71886309	3.9781328	4.2322964	0.00041716	0.01768995	0.08215093
ENSG00000105185.12_PDCD5	PDCD5	programmed cell death 5	HGNC:8764	19q13.11	0.61749946	4.91180722	4.24211448	0.00040774	0.01751883	0.07896978
ENSG00000166068.13_SPRED1	SPRED1	sprouty related EVH1 domain containing 1	HGNC:20249	15q14	-0.7516029	1.5638804	-4.1969325	0.00045292	0.01806256	0.07081501
ENSG00000136295.15_TTYH3	TTYH3	tweety family member 3	HGNC:22222	7p22.3	-1.6670023	1.87263193	-4.1921224	0.00045802	0.01810076	0.0629256
ENSG00000171863.14_RPS7	RPS7	ribosomal protein S7	HGNC:10440	2p25.3	0.55298606	7.47981792	4.23152091	0.00041791	0.01768995	0.05512635
ENSG00000147403.16_RPL10	RPL10	ribosomal protein L10	HGNC:10298	Xq28	0.51093185	9.93367236	4.21665011	0.00043262	0.01794675	0.05174183
ENSG00000111961.18_SASH1	SASH1	SAM and SH3 domain containing 1	HGNC:19182	6q24.3-q25.1	-0.760862	3.56118512	-4.2119517	0.00043737	0.01794675	0.05066363
ENSG00000124562.10_SNRPC	SNRPC	small nuclear ribonucleoprotein polypeptide C	HGNC:11157	6p21.31	0.47366271	5.1800765	4.2306043	0.0004188	0.01768995	0.05024551
ENSG00000131473.17_ACLY	ACLY	ATP citrate lyase	HGNC:115	17q21.2	-0.6188841	3.56162212	-4.2064858	0.00044297	0.01794675	0.03736519
ENSG00000172456.17_FGGY	FGGY	FGGY carbohydrate kinase domain containing	HGNC:25610	1p32.1	0.94927828	4.68252951	4.22023898	0.00042902	0.01791966	0.03734203
ENSG00000205544.4_TMEM256	TMEM256	transmembrane protein 256	HGNC:28618	17p13.1	0.74944835	3.05012535	4.19147504	0.00045871	0.01810076	0.03709716
ENSG00000185361.9_TNFAIP8L1	TNFAIP8L1	TNF alpha induced protein 8 like 1	HGNC:28279	19p13.3	-0.9072875	2.33142885	-4.1804916	0.00047058	0.01828056	0.03613785
ENSG00000129559.13_NEDD8	NEDD8	NEDD8 ubiquitin like modifier	HGNC:7732	14q12	0.59329487	6.04584142	4.22054402	0.00042872	0.01791966	0.02597256
ENSG00000155849.15_ELMO1	ELMO1	engulfment and cell motility 1	HGNC:16286	7p14.2-p14.1	-1.1001877	1.0543172	-4.1893912	0.00046093	0.01811669	0.02317067
ENSG00000171490.13_RSL1D1	RSL1D1	ribosomal L1 domain containing 1	HGNC:24534	16p13.13	0.56848874	6.68295224	4.21675684	0.00043251	0.01794675	0.01882447
ENSG00000167178.16_ISLR2	ISLR2	immunoglobulin superfamily containing leucine rich repeat 2	HGNC:29286	15q24.1	-1.6010982	0.69371167	-4.2155503	0.00043372	0.01794675	0.01697554
ENSG00000158270.12_COLEC12	COLEC12	collectin subfamily member 12	HGNC:16016	18p11.32	-1.2411941	2.30029897	-4.1706403	0.00048149	0.01858945	0.01454811
ENSG00000109686.18_SH3D19	SH3D19	SH3 domain containing 19	HGNC:30418	4q31.3	-0.6584393	3.96683585	-4.2044516	0.00044507	0.01794675	0.01436718
ENSG00000181924.7_COA4	COA4	cytochrome c oxidase assembly factor 4 homolog	HGNC:24604	11q13.4	0.53856797	4.24653804	4.20641291	0.00044304	0.01794675	0.01413232
ENSG00000179958.8_DCTPP1	DCTPP1	dCTP pyrophosphatase 1	HGNC:28777	16p11.2	0.49057304	4.60434641	4.20900017	0.00044038	0.01794675	0.01024296
ENSG00000257303.1_AC073896.2	AC073896.2				1.07102686	1.2018481	4.18518384	0.00046547	0.01812907	0.00835371
ENSG00000104967.7_NOVA2	NOVA2	NOVA alternative splicing regulator 2	HGNC:7887	19q13.32	-1.3077749	1.30861627	-4.1699614	0.00048225	0.01858945	-0.0018625
ENSG00000105583.11_WDR83OS	WDR83OS	WD repeat domain 83 opposite strand	HGNC:30203	19p13.13	0.47880034	5.2600411	4.20401381	0.00044552	0.01794675	-0.009231
ENSG00000101361.17_NOP56	NOP56	NOP56 ribonucleoprotein	HGNC:15911	20p13	0.53934851	5.16358926	4.20199053	0.00044762	0.01794675	-0.0129979
ENSG00000117305.15_HMGCL	HMGCL	3-hydroxy-3-methylglutaryl-CoA lyase	HGNC:5005	1p36.11	0.42971326	5.07557604	4.19970527	0.00045001	0.01799429	-0.0172755
ENSG00000198844.12_ARHGEF15	ARHGEF15	Rho guanine nucleotide exchange factor 15	HGNC:15590	17p13.1	-0.8319803	3.27849911	-4.174718	0.00047694	0.01847988	-0.0172859
ENSG00000090020.11_SLC9A1	SLC9A1	solute carrier family 9 member A1	HGNC:11071	1p36.11	-0.9213105	2.62121418	-4.153873	0.00050064	0.01900531	-0.0245737
ENSG00000114388.12_NPRL2	NPRL2	NPR2 like, GATOR1 complex subunit	HGNC:24969	3p21.31	0.49824646	4.16399985	4.18608719	0.00046449	0.01812907	-0.0276589
ENSG00000071246.11_VASH1	VASH1	vasohibin 1	HGNC:19964	14q24.3	-0.9811999	3.36604883	-4.1675647	0.00048494	0.01859777	-0.032887
ENSG00000125995.16_ROMO1	ROMO1	reactive oxygen species modulator 1	HGNC:16185	20q11.22	0.66862564	5.54437777	4.19270727	0.00045739	0.01810076	-0.035153
ENSG00000113721.14_PDGFRB	PDGFRB	platelet derived growth factor receptor beta	HGNC:8804	5q32	-0.9127923	5.08852014	-4.1884497	0.00046195	0.01811669	-0.0414062
ENSG00000140479.17_PCSK6	PCSK6	proprotein convertase subtilisin/kexin type 6	HGNC:8569	15q26.3	-1.473045	2.07126678	-4.1412785	0.00051553	0.0192297	-0.0430594
ENSG00000146083.12_RNF44	RNF44	ring finger protein 44	HGNC:19180	5q35.2	-0.596224	3.62902603	-4.1630789	0.00049003	0.01869724	-0.0581427
ENSG00000112357.13_PEX7	PEX7	peroxisomal biogenesis factor 7	HGNC:8860	6q23.3	0.61144228	2.42501903	4.12991723	0.00052934	0.01940689	-0.0711045
ENSG00000166503.9_HDGFL3	HDGFL3	HDGF like 3	HGNC:24937	15q25.2	-0.6747191	2.47115944	-4.1316381	0.00052722	0.01939047	-0.0732484
ENSG00000162910.19_MRPL55	MRPL55	mitochondrial ribosomal protein L55	HGNC:16686	1q42.13	0.52064837	4.11280802	4.16407198	0.0004889	0.01869724	-0.0741141
ENSG00000171522.6_PTGER4	PTGER4	prostaglandin E receptor 4	HGNC:9596	5p13.1	-1.0929202	1.3465899	-4.1322108	0.00052652	0.01939047	-0.0744696
ENSG00000130032.16_PRRG3	PRRG3	proline rich and Gla domain 3	HGNC:30798	Xq28	-1.3516172	0.33124219	-4.2027989	0.00044678	0.01794675	-0.0837999
ENSG00000153815.16_CMIP	CMIP	c-Maf inducing protein	HGNC:24319	16q23.2-q23.	-1.4298618	1.41380443	-4.1259751	0.00053422	0.01953796	-0.0852923
ENSG00000103111.14_MON1B	MON1B	MON1 homolog B, secretory trafficking associated	HGNC:25020	16q23.1	-0.5622037	2.84999603	-4.1316069	0.00052726	0.01939047	-0.0895125
ENSG00000166352.16_C11orf74	C11orf74	intraflagellar transport associated protein	HGNC:25142	11p12	0.71754147	3.29226188	4.14008549	0.00051696	0.0192353	-0.0899927
ENSG00000167861.16_HID1	HID1	HID1 domain containing	HGNC:15736	17q25.1	-0.935282	2.18556322	-4.1127635	0.00055089	0.01985734	-0.1047646
ENSG00000128641.19_MYO1B	MYO1B	myosin IB	HGNC:7596	2q32.3	-0.5775906	2.74211619	-4.123492	0.00053731	0.01955578	-0.1084419
ENSG00000136546.15_SCN7A	SCN7A	sodium voltage-gated channel alpha subunit 7	HGNC:10594	2q24.3	-1.4373456	1.77703052	-4.1056368	0.00056011	0.02004895	-0.1155555
ENSG00000174748.20_RPL15	RPL15	ribosomal protein L15	HGNC:10306	3p24.2	0.47395304	8.64127589	4.1483036	0.00050717	0.01912776	-0.1157576
ENSG00000125691.13_RPL23	RPL23	ribosomal protein L23	HGNC:10316	17q12	0.61611871	8.40542789	4.14991345	0.00050527	0.01912776	-0.1159972
ENSG00000153214.11_TMEM87B	TMEM87B	transmembrane protein 87B	HGNC:25913	2q13	-0.7530323	1.0545069	-4.1142514	0.00054899	0.01983639	-0.1239971

ENSG00000172428.11_COPS9	COPS9	COP9 signalosome subunit 9	HGNC:21314	2q37.3	0.71127276	5.50144549	4.14690356	0.00050882	0.01912776	-0.1364457
ENSG00000100387.8_RBX1	RBX1	ring-box 1	HGNC:9928	22q13.2	0.57667202	5.51429925	4.14678213	0.00050897	0.01912776	-0.136925
ENSG00000181104.7_F2R	F2R	coagulation factor II thrombin receptor	HGNC:3537	5q13.3	-1.1769317	1.13021806	-4.1023391	0.00056442	0.02010329	-0.1406222
ENSG00000074201.8_CLNS1A	CLNS1A	chloride nucleotide-sensitive channel 1A	HGNC:2080	11q14.1	0.46774956	5.0728839	4.14247783	0.00051409	0.01922393	-0.1439162
ENSG00000157554.19_ERG	ERG	ETS transcription factor ERG	HGNC:3446	21q22.2	-0.8758433	2.40418892	-4.0950813	0.00057403	0.02034903	-0.1498974
ENSG00000164405.11_UQCRQ	UQCRQ	ubiquinol-cytochrome c reductase complex III subunit VII	HGNC:29594	5q31.1	0.68236164	7.94013199	4.13623556	0.00052161	0.01936034	-0.1518037
ENSG00000277258.5_PCGF2	PCGF2	polycomb group ring finger 2	HGNC:12929	17q12	-1.0381289	1.53558955	-4.0874586	0.00058431	0.02051241	-0.1577203
ENSG00000184005.11_ST6GALNAC3	ST6GALNAC3	ST6 N-acetylgalactosaminide alpha-2,6-sialyltransferase 3	HGNC:19343	1p31.1	-1.1263484	-0.1780556	-4.1928509	0.00045724	0.01810076	-0.1788083
ENSG00000136930.13_PSMB7	PSMB7	proteasome 20S subunit beta 7	HGNC:9544	9q33.3	0.59889243	6.0956988	4.12443104	0.00053614	0.01955578	-0.1867826
ENSG00000130332.15_LSM7	LSM7	LSM7 homolog, U6 small nuclear RNA and mRNA degradation	HGNC:20470	19p13.3	0.59547523	3.85263984	4.1055487	0.00056022	0.02004895	-0.1919864
ENSG00000152784.15_PRDM8	PRDM8	PR/SET domain 8	HGNC:13993	4q21.21	-1.4568035	0.14779763	-4.1446021	0.00051156	0.01917697	-0.1945386
ENSG00000099795.7_NDUFB7	NDUFB7	NADH:ubiquinone oxidoreductase subunit B7	HGNC:7702	19p13.12	0.54766004	7.05271932	4.11976959	0.00054199	0.01967814	-0.1945932
ENSG00000145358.6_DDIT4L	DDIT4L	DNA damage inducible transcript 4 like	HGNC:30555	4q24	1.05661411	7.31286059	4.11853683	0.00054354	0.019687	-0.1961959
ENSG00000132669.13_RIN2	RIN2	Ras and Rab interactor 2	HGNC:18750	20p11.23	-0.5943164	3.09285351	-4.086173	0.00058606	0.02051241	-0.2005135
ENSG00000138594.14_TMOD3	TMOD3	tropomodulin 3	HGNC:11873	15q21.2	-0.7424194	2.63260092	-4.0739268	0.000603	0.02073517	-0.2028698
ENSG00000134780.10_DAGLA	DAGLA	diacylglycerol lipase alpha	HGNC:1165	11q12.2	-1.0449516	1.44357778	-4.0652213	0.00061534	0.02092107	-0.2105153
ENSG00000173269.14_MMRN2	MMRN2	multimerin 2	HGNC:19888	10q23.2	-0.8320575	4.18402423	-4.1026411	0.00056402	0.02010329	-0.2150234
ENSG00000132676.16_DAP3	DAP3	death associated protein 3	HGNC:2673	1q22	0.538129	5.53926334	4.11154358	0.00055246	0.01986603	-0.2151698
ENSG00000120708.17_TGFBI	TGFBI	transforming growth factor beta induced	HGNC:11771	5q31.1	-1.1747404	3.83365905	-4.0961596	0.0005726	0.02034614	-0.2155149
ENSG00000272674.3_PCDHB16	PCDHB16	protocadherin beta 16	HGNC:14546	5q31.3	-1.4489794	-0.505682	-4.2362335	0.00041336	0.01760869	-0.2359565
ENSG00000100575.14_TIMM9	TIMM9	translocase of inner mitochondrial membrane 9	HGNC:11819	14q23.1	0.5669893	3.57540633	4.07873274	0.00059629	0.02068361	-0.2375171
ENSG00000180357.9_ZNF609	ZNF609	zinc finger protein 609	HGNC:29003	15q22.1	-0.486073	3.61047055	-4.0819455	0.00059185	0.02063911	-0.2378523
ENSG00000137478.15_FCHSD2	FCHSD2	FCH and double SH3 domains 2	HGNC:29114	11q13.4	-0.6186894	2.60924965	-4.0567735	0.00062756	0.02109913	-0.2422666
ENSG00000133997.11_MED6	MED6	mediator complex subunit 6	HGNC:19970	14q24.2	0.52468866	3.40372691	4.07314684	0.0006041	0.02073517	-0.2430619
ENSG00000176731.12_C8orf59	C8orf59	ribosomal biogenesis factor	HGNC:32235	8q21.2	0.58929594	3.78316433	4.07936395	0.00059542	0.02068361	-0.2467099
ENSG00000124496.12_TRERF1	TRERF1	transcriptional regulating factor 1	HGNC:18273	6p21.1	-1.4546688	0.27316858	-4.131333	0.0005276	0.01939047	-0.2498416
ENSG00000130589.16_HELZ2	HELZ2	helicase with zinc finger 2	HGNC:30021	20q13.33	-1.5420487	2.26761398	-4.0428745	0.00064819	0.02136165	-0.250073
ENSG00000101470.10_TNNC2	TNNC2	troponin C2, fast skeletal type	HGNC:11944	20q13.12	0.92886015	12.7816461	4.06428683	0.00061668	0.02092107	-0.2527358
ENSG00000179583.19_CIITA	CIITA	class II major histocompatibility complex transactivator	HGNC:7067	16p13.13	-0.8228285	3.1917026	-4.05953	0.00062354	0.02105866	-0.2561641
ENSG00000177700.6_POLR2L	POLR2L	RNA polymerase II subunit L	HGNC:9199	11p15.5	0.59617503	6.00031228	4.09306127	0.00057674	0.02039669	-0.2562981
ENSG00000100804.18_PSMB5	PSMB5	proteasome 20S subunit beta 5	HGNC:9542	14q11.2	0.4891738	6.76621451	4.09146634	0.00057888	0.02042436	-0.25819
ENSG00000170290.4_SLN	SLN	sarcolipin	HGNC:11089	11q22.3	0.77985856	11.0800749	4.06779805	0.00061166	0.02087054	-0.2622473
ENSG00000126603.8_GLIS2	GLIS2	GLIS family zinc finger 2	HGNC:29450	16p13.3	-1.2752063	1.57270932	-4.0330892	0.00066312	0.02161611	-0.2701323
ENSG00000089289.16_IGBP1	IGBP1	immunoglobulin binding protein 1	HGNC:5461	Xq13.1	0.62513563	5.65015986	4.08559226	0.00058685	0.02051241	-0.2726748
ENSG00000134825.15_TMEM258	TMEM258	transmembrane protein 258	HGNC:1164	11q12.2	0.58578732	4.78375096	4.07803238	0.00059727	0.02068361	-0.2816811
ENSG00000125445.11_MRPS7	MRPS7	mitochondrial ribosomal protein S7	HGNC:14499	17q25.1	0.49382264	6.34782254	4.07668194	0.00059915	0.02070091	-0.2920113
ENSG00000127540.12_UQCR11	UQCR11	ubiquinol-cytochrome c reductase, complex III subunit XI	HGNC:30862	19p13.3	0.65059793	8.0112147	4.0711729	0.00060688	0.02077651	-0.2947264
ENSG00000167863.12_ATP5PD	ATP5PD	ATP synthase peripheral stalk subunit d	HGNC:845	17q25.1	0.62479606	7.40643363	4.073011	0.00060429	0.02073517	-0.2958302
ENSG00000102870.6_ZNF629	ZNF629	zinc finger protein 629	HGNC:29008	16p11.2	-0.6506467	3.80597576	-4.0527661	0.00063344	0.02115452	-0.3035945
ENSG00000265681.7_RPL17	RPL17	ribosomal protein L17	HGNC:10307	18q21.1	0.55246598	7.53744378	4.0672749	0.00061241	0.02087054	-0.3076833
ENSG00000219410.6_AC125494.1	AC125494.1				0.83610184	1.98670474	4.01190894	0.00069662	0.02235191	-0.3110755
ENSG00000058668.14_ATP2B4	ATP2B4	ATPase plasma membrane Ca2+ transporting 4	HGNC:817	1q32.1	-0.7169621	4.48670908	-4.0630752	0.00061842	0.02093278	-0.3114393
ENSG00000162618.14_ADGRL4	ADGRL4	adhesion G protein-coupled receptor L4	HGNC:20822	1p31.1	-0.7865445	2.71841074	-4.020113	0.00068344	0.02208672	-0.3238127
ENSG00000107736.20_CDH23	CDH23	cadherin related 23	HGNC:13733	10q22.1	-1.1189847	2.46376217	-4.0121591	0.00069621	0.02235191	-0.3288465
ENSG00000124614.15_RPS10	RPS10	ribosomal protein S10	HGNC:10383	6p21.31	0.56072878	7.87929205	4.05514948	0.00062993	0.02113164	-0.3316654
ENSG00000175061.17_SNHG29	SNHG29	small nucleolar RNA host gene 29	HGNC:28619	17p11.2	0.44314174	5.56902367	4.05789596	0.00062592	0.02109138	-0.3339472
ENSG00000254093.9_PINX1	PINX1	PIN2 (TERF1) interacting telomerase inhibitor 1	HGNC:30046	8p23.1	0.63900958	1.85758612	3.99763002	0.00072015	0.0228785	-0.3401531
ENSG00000082014.16_SMARCD3	SMARCD3	SWI/SNF related, matrix associated, actin dependent regulato	HGNC:11108	7q36.1	0.44287466	6.87054379	4.05139724	0.00063546	0.02117485	-0.3466756
ENSG00000082497.12_SERTAD4	SERTAD4	SERTA domain containing 4	HGNC:25236	1q32.2	-1.140684	-0.5244348	-4.1609669	0.00049245	0.01874165	-0.3471559
ENSG00000126878.13_AIF1L	AIF1L	allograft inflammatory factor 1 like	HGNC:28904	9q34.12-q34.	-0.671172	3.71801947	-4.0310349	0.00066629	0.02162567	-0.3555531
ENSG00000165916.8_PSMC3	PSMC3	proteasome 26S subunit, ATPase 3	HGNC:9549	11p11.2	0.59948166	7.04897901	4.04579688	0.00064379	0.02135769	-0.3581385
ENSG00000115159.16_GPD2	GPD2	glycerol-3-phosphate dehydrogenase 2	HGNC:4456	2q24.1	0.79977078	5.07085504	4.04457433	0.00064563	0.02136165	-0.3594045
ENSG00000198918.8_RPL39	RPL39	ribosomal protein L39	HGNC:10350	Xq24	0.64841671	7.74279187	4.04308613	0.00064787	0.02136165	-0.3595031
ENSG00000152147.11_GEMIN6	GEMIN6	gem nuclear organelle associated protein 6	HGNC:20044	2p22.1	0.6330269	1.93426146	3.98148967	0.00074771	0.02332442	-0.3744166
ENSG00000062582.14_MRPS24	MRPS24	mitochondrial ribosomal protein S24	HGNC:14510	7p13	0.49580821	6.56411316	4.03680829	0.0006574	0.02161611	-0.3797354
ENSG00000184254.17_ALDH1A3	ALDH1A3	aldehyde dehydrogenase 1 family member A3	HGNC:409	15q26.3	-1.2792504	1.91451493	-3.9782404	0.00075338	0.02338702	-0.3820264
ENSG00000167548.15_KMT2D	KMT2D	lysine methyltransferase 2D	HGNC:7133	12q13.12	-0.7303856	6.35808331	-4.0343826	0.00066112	0.02161611	-0.3850606
ENSG00000198682.13_PAPSS2	PAPSS2	3'-phosphoadenosine 5'-phosphosulfate synthase 2	HGNC:8604	10q23.2-q23.	-1.1637936	1.12777464	-3.9803231	0.00074974	0.02332442	-0.3857259
ENSG00000134198.10_TSPAN2	TSPAN2	tetraspanin 2	HGNC:20659	1p13.2	-1.0540121	0.16974915	-4.0319323	0.0006649	0.02162567	-0.3862272
ENSG00000008018.9_PSMB1	PSMB1	proteasome 20S subunit beta 1	HGNC:9537	6q27	0.60537461	6.45981498	4.03351439	0.00066246	0.02161611	-0.3872813
ENSG00000242485.6_MRPL20	MRPL20	mitochondrial ribosomal protein L20	HGNC:14478	1p36.33	0.57720149	6.00799804	4.03328575	0.00066281	0.02161611	-0.3885267
ENSG00000129007.14_CALML4	CALML4	calmodulin like 4	HGNC:18445	15q23	1.80813007	1.25057338	3.99599838	0.00072289	0.02291697	-0.3896912
ENSG00000131323.14_TRAF3	TRAF3	TNF receptor associated factor 3	HGNC:12033	14q32.32	-0.5967044	2.61940415	-3.9803771	0.00074965	0.02332442	-0.4071824
ENSG00000260465.1_AC018557.1	AC018557.1				0.74834516	3.13970063	3.98879726	0.0007351	0.02310876	-0.4089496
ENSG00000232388.4_SMIM26	SMIM26	small integral membrane protein 26	HGNC:43430	20p11.23	0.61868753	5.51608957	4.02135186	0.00068148	0.02207071	-0.4144899
ENSG00000167515.10_TRAPPC2L	TRAPPC2L	trafficking protein particle complex 2 like	HGNC:30887	16q24.3	0.53107801	4.53730094	4.01129495	0.00069761	0.02235191	-0.4231881
ENSG00000158769.18_F11R	F11R	F11 receptor	HGNC:14685	1q23.3	-0.8998599	2.48838143	-3.9650246	0.00077691	0.02382356	-0.4266826
ENSG00000135111.16_TBX3	TBX3	T-box transcription factor 3	HGNC:11602	12q24.21	-0.8081531	2.98990987	-3.9750882	0.00075893	0.02346419	-0.4365503
ENSG00000104979.9_C19orf53	C19orf53	chromosome 19 open reading frame 53	HGNC:24991	19p13.13	0.56635367	5.66467479	4.01144645	0.00069737	0.02235191	-0.4367312
ENSG00000160948.14_VPS28	VPS28	VPS28 subunit of ESCRT-I	HGNC:18178	8q24.3	0.48031373	5.82162155	4.01013987	0.00069949	0.02236428	-0.4398113
ENSG00000279598.1_AC009948.4	AC009948.4				1.87348472	-0.9180189	4.29227017	0.00036287	0.01663961	-0.4423187
ENSG00000214176.9_PLEKHM1P1	PLEKHM1P1	pleckstrin homology and RUN domain containing M1 pseudog	HGNC:35411	17q24.1	-0.6652108	2.57325447	-3.9586732	0.00078847	0.02402951	-0.4464109
ENSG00000082068.8_WDR70	WDR70	WD repeat domain 70	HGNC:25495	5p13.2	0.5316267	3.55888967	3.97739123	0.00075487	0.02338702	-0.462503
ENSG00000126883.17_NUP214	NUP214	nucleoporin 214	HGNC:8064	9q34.13	-0.450084	4.18752535	-3.9893834	0.0007341	0.02310876	-0.4632372
ENSG00000132432.14_SEC61G	SEC61G	SEC61 translocon subunit gamma	HGNC:18277	7p11.2	0.5921633	4.55073228	3.99219878	0.00072931	0.02303961	-0.466569
ENSG00000124593.16_AL365205.1	AL365205.1				-0.7782701	2.10183379	-3.9380689	0.00082718	0.02470841	-0.4698659
ENSG00000118257.16_NRP2	NRP2	neuropilin 2	HGNC:8005	2q33.3	-1.0128275	1.50526803	-3.9341175	0.00083482	0.02488698	-0.4720617
ENSG00000119541.10_VPS4B	VPS4B	vacuolar protein sorting 4 homolog B	HGNC:10895	18q21.33	-0.6089349	2.71313986	-3.9495601	0.00080537	0.0243477	-0.4742895
ENSG00000177425.11_PAWR	PAWR	pro-apoptotic WT1 regulator	HGNC:8614	12q21.2	-0.9171973	0.59864377	-3.9589026	0.00078805	0.02402951	-0.4786001
ENSG00000182117.5_NOP10	NOP10	NOP10 ribonucleoprotein	HGNC:14378	15q14	0.62262299	4.59789487	3.98667664	0.00073874	0.02317448	-0.479898
ENSG00000067167.8_TRAM1	TRAM1	translocation associated membrane protein 1	HGNC:20568	8q13.3	-0.5047975	4.17047727	-3.9804542	0.00074951	0.02332442	-0.4849065
ENSG00000173548.8_SNX33	SNX33	sorting nexin 33	HGNC:28468	15q24.2	-0.9015473	2.94807617	-3.9414076	0.00082079	0.02457222	-0.5078614
ENSG00000104067.16_TJP1	TJP1	tight junction protein 1	HGNC:11827	15q13.1	-0.5287535	4.42332715	-3.9689082	0.00076992	0.02365767	-0.5178549
ENSG00000127774.7_EMC6	EMC6	ER membrane protein complex subunit 6	HGNC:28430	17p13.2	0.49793002	5.47008287	3.96990937	0.00076813	0.02365109	-0.5281797
ENSG00000181610.13_MRPS23	MRPS23	mitochondrial ribosomal protein S23	HGNC:14509	17q22	0.4931466	4.20096099	3.9578228	0.00079003	0.02402951	-0.5310755
ENSG00000130052.13_STARD8	STARD8	StAR related lipid transfer domain containing 8	HGNC:19161	Xq13.1	-0.6494307	2.78201646	-3.9222105	0.00085827	0.02547415	-0.5333522
ENSG00000108953.17_YWHAE	YWHAE	tyrosine 3-monooxygenase/tryptophan 5-monooxygenase ac	HGNC:12851	17p13.3	0.40919995	8.38521811	3.9589887	0.00078789	0.02402951	-0.5369157
ENSG00000139154.15_AEBP2	AEBP2	AE binding protein 2	HGNC:24051	12p12.3	-0.5795439	2.7643863	-3.921535	0.00085962	0.02547415	-0.5387159
ENSG00000105339.10_DENND3	DENND3	DENN domain containing 3	HGNC:29134	8q24.3	-0.747909	2.94176678	-3.9256445	0.00085144	0.02533195	-0.539872
ENSG00000146535.14_GNA12	GNA12	G protein subunit alpha 12	HGNC:4380	7p22.3-p22.2	-0.5566712	3.52532116	-3.9421174	0.00081943	0.02457222	-0.5412342
ENSG00000075618.18_FSCN1	FSCN1	fascin actin-bundling protein 1	HGNC:11148	7p22.1	-1.3921653	2.21230015	-3.9035479	0.00089634	0.02605964	-0.5433086
ENSG00000084072.16_PPIE	PPIE	peptidylprolyl isomerase E	HGNC:9258	1p34.2	0.41528494	4.52930684	3.95428346	0.00079657	0.02417914	-0.5503105
ENSG00000164736.6_SOX17	SOX17	SRY-box transcription factor 17	HGNC:18122	8q11.23	-1.0113295	1.03738834	-3.8941605	0.00091612	0.02630824	-0.5705532
ENSG00000179271.3_GADD45GIP1	GADD45GIP1	GADD45G interacting protein 1	HGNC:29996	19p13.13	0.6449485	6.17530255	3.95074478	0.00080315	0.02432975	-0.5708018
ENSG00000167874.6_TMEM88	TMEM88	transmembrane protein 88	HGNC:32371	17p13.1	-0.7420496	2.09521306	-3.8886616	0.00092791	0.02645237	-0.574107
ENSG00000155363.18_MOV10	MOV10	Mov10 RISC complex RNA helicase	HGNC:7200	1p13.2	-0.7359657	2.90386856	-3.9051645	0.00089297	0.02605964	-0.5779333
ENSG00000117450.14_PRDX1	PRDX1	peroxiredoxin 1	HGNC:9352	1p34.1	0.56569902	6.97948289	3.94300069	0.00081775	0.02457222	-0.5853745
ENSG00000210151.2_MT-TS1	MT-TS1	mitochondrially encoded tRNA-Ser (UCN) 1	HGNC:7497	mitochondria	1.60186276	0.04308635	4.00273892	0.00071164	0.02270434	-0.5887653
ENSG00000153827.13_TRIP12	TRIP12	thyroid hormone receptor interactor 12	HGNC:12306	2q36.3	0.49440232	6.3914	3.94144977	0.0008207	0.02457222	-0.5907914
ENSG00000129657.16_SEC14L1	SEC14L1	SEC14 like lipid binding 1	HGNC:10698	17q25.2-q25.	-0.4371059	5.76091195	-3.9413023	0.00082099	0.02457222	-0.5916411
ENSG00000179899.8_PHC1P1	PHC1P1	polyhomeotic homolog 1 pseudogene 1	HGNC:34502	12q13.2	-1.80011	-0.0631614	-3.9981389	0.0007193	0.0228785	-0.5972119

ENSG00000204248.10_COL11A2	COL11A2	collagen type XI alpha 2 chain	HGNC:2187	6p21.32	-1.1183805	1.91221353	-3.8724136	0.00096363	0.02697073	-0.601482
ENSG00000219626.9_FAM228B	FAM228B	family with sequence similarity 228 member B	HGNC:24736	2p23.3	0.76305378	1.51804436	3.87141231	0.00096587	0.02697073	-0.6023979
ENSG00000081818.3_PCDHB4	PCDHB4	protocadherin beta 4	HGNC:8689	5q31.3	-1.4405109	-0.0983067	-3.9918912	0.00072983	0.02303961	-0.6049014
ENSG00000267645.5_AC105052.3	AC105052.3				3.95572223	-1.0816791	4.25152423	0.00039892	0.01738819	-0.6108323
ENSG00000229833.9_PET100	PET100	PET100 cytochrome c oxidase chaperone	HGNC:40038	19p13.2	0.70658525	4.05339974	3.91503978	0.0008727	0.02576007	-0.6174817
ENSG00000269893.7_SNHG8	SNHG8	small nucleolar RNA host gene 8	HGNC:33098	4q26	0.60771417	3.47411729	3.90298672	0.00089751	0.02605964	-0.6193675
ENSG00000135439.11_AGAP2	AGAP2	ArfGAP with GTPase domain, ankyrin repeat and PH domain 2	HGNC:16921	12q14.1	-0.9445402	3.55978079	-3.9037166	0.00089598	0.02605964	-0.6224679
ENSG00000120594.17_PLXDC2	PLXDC2	plexin domain containing 2	HGNC:21013	10p12.31	-0.7164692	3.27485927	-3.897124	0.00090983	0.02620087	-0.6240299
ENSG00000187474.5_FPR3	FPR3	formyl peptide receptor 3	HGNC:3828	19q13.41	-2.1523751	-0.9824269	-4.1682487	0.00048417	0.01859777	-0.6326353
ENSG00000183580.10_FBXL7	FBXL7	F-box and leucine rich repeat protein 7	HGNC:13604	5p15.1	-0.8459617	1.23505157	-3.8560384	0.00100102	0.02754171	-0.6360333
ENSG00000198258.10_UBL5	UBL5	ubiquitin like 5	HGNC:13736	19p13.2	0.63334678	6.31102873	3.92004176	0.00086261	0.02551235	-0.6383005
ENSG00000149564.12_ESAM	ESAM	endothelial cell adhesion molecule	HGNC:17474	11q24.2	-0.7803918	3.94389879	-3.9025165	0.00089849	0.02605964	-0.6455718
ENSG00000169189.17_NSMCE1	NSMCE1	NSE1 homolog, SMC5-SMC6 complex component	HGNC:29897	16p12.1	0.5069384	4.10503552	3.90488048	0.00089356	0.02605964	-0.6463964
ENSG00000106571.14_GLI3	GLI3	GLI family zinc finger 3	HGNC:4319	7p14.1	-0.8900324	0.63455468	-3.8648378	0.00098075	0.02713346	-0.6486174
ENSG00000053918.16_KCNQ1	KCNQ1	potassium voltage-gated channel subfamily Q member 1	HGNC:6294	11p15.5-p15.	-1.622771	-0.3446998	-3.9700521	0.00076787	0.02365109	-0.6550741
ENSG00000155034.19_FBXL18	FBXL18	F-box and leucine rich repeat protein 18	HGNC:21874	7p22.1	-0.7732415	2.87216326	-3.8666192	0.0009767	0.02707128	-0.6575105
ENSG00000144619.15_CNTN4	CNTN4	contactin 4	HGNC:2174	3p26.3-p26.2	-2.0994314	-1.3890987	-4.1877164	0.00046273	0.01811669	-0.6588635
ENSG00000152518.8_ZFP36L2	ZFP36L2	ZFP36 ring finger protein like 2	HGNC:1108	2p21	-0.7322447	4.88743294	-3.9048658	0.00089359	0.02605964	-0.6659856
ENSG00000103222.19_ABCC1	ABCC1	ATP binding cassette subfamily C member 1	HGNC:51	16p13.11	-0.6096864	4.59166274	-3.898271	0.0009074	0.02620087	-0.6748528
ENSG00000113312.11_TTC1	TTC1	tetratricopeptide repeat domain 1	HGNC:12391	5q33.3	0.50077651	5.55176448	3.90273181	0.00089804	0.02605964	-0.6767354
ENSG00000162522.11_KIAA1522	KIAA1522	KIAA1522	HGNC:29301	1p35.1	-1.1486877	1.71657842	-3.8337775	0.00105417	0.02868824	-0.6803028
ENSG00000084754.12_HADHA	HADHA	hydroxyacyl-CoA dehydrogenase trifunctional multienzyme cc	HGNC:4801	2p23.3	0.4225713	8.32935783	3.89292219	0.00091876	0.02630824	-0.682655
ENSG00000163453.11_IGFBP7	IGFBP7	insulin like growth factor binding protein 7	HGNC:5476	4q12	-0.6825846	6.08877158	-3.8979888	0.000908	0.02620087	-0.6866339
ENSG00000136861.18_CDK5RAP2	CDK5RAP2	CDK5 regulatory subunit associated protein 2	HGNC:18672	9q33.2	0.41638754	5.87480016	3.89687772	0.00091035	0.02620087	-0.6899708
ENSG00000198668.11_CALM1	CALM1	calmodulin 1	HGNC:1442	14q32.11	0.55080459	8.7741071	3.88442628	0.00093709	0.02661296	-0.6956191
ENSG00000065427.14_KARS	KARS	lysyl-tRNA synthetase 1	HGNC:6215	16q23.1	0.47220862	7.02661665	3.89264981	0.00091934	0.02630824	-0.6962411
ENSG00000061337.15_LZTS1	LZTS1	leucine zipper tumor suppressor 1	HGNC:13861	8p21.3	-1.2775263	0.34774674	-3.8590792	0.00099397	0.02739801	-0.6989124
ENSG00000126756.12_UXT	UXT	ubiquitously expressed prefoldin like chaperone	HGNC:12641	Xp11.23	0.5960211	4.01578114	3.87782227	0.00095159	0.02697073	-0.6996454
ENSG00000170906.15_NDUFA3	NDUFA3	NADH:ubiquinone oxidoreductase subunit A3	HGNC:7686	19q13.42	0.75460169	6.54656048	3.89175267	0.00092126	0.02631296	-0.7000101
ENSG00000147100.11_SLC16A2	SLC16A2	solute carrier family 16 member 2	HGNC:10923	Xq13.2	-0.9753665	1.41781339	-3.8229644	0.00108099	0.02900515	-0.7003654
ENSG00000115468.12_EFHD1	EFHD1	EF-hand domain family member D1	HGNC:29556	2q37.1	-0.6591079	3.45107576	-3.8681641	0.0009732	0.02702418	-0.7005169
ENSG00000249437.7_NAIP	NAIP	NLR family apoptosis inhibitory protein	HGNC:7634	5q13.2	-1.3897651	1.6200146	-3.8228477	0.00108128	0.02900515	-0.7007564
ENSG00000220842.6_RPL21P16	RPL21P16	ribosomal protein L21 pseudogene 16	HGNC:31396	10q26.12	0.92495257	2.11210828	3.82388643	0.00107867	0.02900515	-0.7051327
ENSG00000204628.11_RACK1	RACK1	receptor for activated C kinase 1	HGNC:4399	5q35.3	0.52071517	8.72827867	3.87703413	0.00095333	0.02697073	-0.7121694
ENSG00000197183.14_NOL4L	NOL4L	nucleolar protein 4 like	HGNC:16106	20q11.21	-1.153285	0.88811012	-3.822638	0.00108181	0.02900515	-0.7246551
ENSG00000240801.1_AC132217.1	AC132217.1				1.1076211	1.63835543	3.8102203	0.00111347	0.02954816	-0.7268258
ENSG00000100129.18_EIF3L	EIF3L	eukaryotic translation initiation factor 3 subunit L	HGNC:18138	22q13.1	0.4453972	7.87407412	3.87545072	0.00095685	0.02697073	-0.7276419
ENSG00000157103.12_SLC6A1	SLC6A1	solute carrier family 6 member 1	HGNC:11042	3p25.3	-1.2090769	0.89338203	-3.8128695	0.00110664	0.02947432	-0.729973
ENSG00000138696.10_BMPR1B	BMPR1B	bone morphogenetic protein receptor type 1B	HGNC:1077	4q22.3	0.87873846	1.2795475	3.80860496	0.00111766	0.02955776	-0.7334324
ENSG00000019549.12_SNAI2	SNAI2	snail family transcriptional repressor 2	HGNC:11094	8q11.21	-0.8990546	0.88136165	-3.8131831	0.00110583	0.02947432	-0.7350312
ENSG00000126267.10_COX6B1	COX6B1	cytochrome c oxidase subunit 6B1	HGNC:2280	19q13.12	0.62034281	7.75936298	3.87207469	0.00096439	0.02697073	-0.7363385
ENSG00000119705.9_SLIRP	SLIRP	SRA stem-loop interacting RNA binding protein	HGNC:20495	14q24.3	0.62917287	5.28992618	3.87480623	0.00095828	0.02697073	-0.7366518
ENSG00000196683.10_TOMM7	TOMM7	translocase of outer mitochondrial membrane 7	HGNC:21648	7p15.3	0.55579375	7.49711835	3.87244107	0.00096357	0.02697073	-0.737666
ENSG00000186184.17_POLR1D	POLR1D	RNA polymerase I and III subunit D	HGNC:20422	13q12.2	0.47256926	5.27672372	3.87389653	0.00096031	0.02697073	-0.7391671
ENSG00000196730.13_DAPK1	DAPK1	death associated protein kinase 1	HGNC:2674	9q21.33	-0.9696873	1.4140355	-3.8036162	0.00113068	0.02970623	-0.7401448
ENSG00000175334.8_BANF1	BANF1	BAF nuclear assembly factor 1	HGNC:17397	11q13.1	0.40389302	7.07245214	3.87059411	0.00096771	0.02697185	-0.7447356
ENSG00000163933.10_RFT1	RFT1	RFT1 homolog	HGNC:30220	3p21.1	-0.6236134	1.71837851	-3.7907198	0.00116506	0.02988719	-0.767521
ENSG00000091640.8_SPAG7	SPAG7	sperm associated antigen 7	HGNC:11216	17p13.2	0.6376235	6.58577017	3.85942178	0.00099317	0.02739801	-0.7713078
ENSG00000085511.20_MAP3K4	MAP3K4	mitogen-activated protein kinase kinase kinase 4	HGNC:6856	6q26	0.49491275	4.05276833	3.84662252	0.00102316	0.02799697	-0.7716262
ENSG00000174944.9_P2RY14	P2RY14	purinergic receptor P2Y14	HGNC:16442	3q25.1	-1.5329462	0.15623174	-3.8297076	0.00106418	0.028804	-0.7824068
ENSG00000227051.6_C14orf132	C14orf132	chromosome 14 open reading frame 132	HGNC:20346	14q32.2	-1.2427167	1.6928556	-3.7837801	0.00118399	0.03011981	-0.7851469
ENSG00000155962.13_CLIC2	CLIC2	chloride intracellular channel 2	HGNC:2063	Xq28	-0.8310104	1.26572515	-3.7795702	0.00119562	0.0301316	-0.7893986
ENSG00000139874.6_SSTR1	SSTR1	somatostatin receptor 1	HGNC:11330	14q13	-1.792891	-1.0852811	-4.0529497	0.00063317	0.02115452	-0.7943189
ENSG00000196704.12_AMZ2	AMZ2	archaelysin family metallopeptidase 2	HGNC:28041	17q24.2	0.42149247	5.50621366	3.84897475	0.00101759	0.02789524	-0.7952047
ENSG00000162836.12_ACP6	ACP6	acid phosphatase 6, lysophosphatidic	HGNC:29609	1q21.2	0.79621351	2.62420583	3.79210383	0.00116132	0.02988719	-0.7952865
ENSG00000196411.10_EPHB4	EPHB4	EPH receptor B4	HGNC:3395	7q22.1	-1.0915892	2.93030467	-3.8043046	0.00112888	0.02970623	-0.7998527
ENSG00000109472.14_CPE	CPE	carboxypeptidase E	HGNC:2303	4q32.3	-0.7703055	4.40879326	-3.8384672	0.00104274	0.02842889	-0.8038667
ENSG00000079308.19_TNS1	TNS1	tensin 1	HGNC:11973	2q35	-0.712647	7.93293826	-3.839383	0.00104052	0.02842013	-0.8038723
ENSG00000178904.19_DPY19L3	DPY19L3	dpy-19 like C-mannosyltransferase 3	HGNC:27120	19q13.11	-0.7179597	1.22762011	-3.7697472	0.0012232	0.03053395	-0.8114153
ENSG00000148444.16_COMMD3	COMMD3	COMM domain containing 3	HGNC:23332	10p12.2	0.51109034	4.87491294	3.83236446	0.00105763	0.02873045	-0.8257977
ENSG00000130816.16_DNMT1	DNMT1	DNA methyltransferase 1	HGNC:2976	19p13.2	-0.5724171	3.11437427	-3.7969497	0.00114833	0.02984254	-0.8285803
ENSG00000106560.11_GIMAP2	GIMAP2	GTPase, IMAP family member 2	HGNC:21789	7q36.1	-0.8836459	0.45189733	-3.7915484	0.00116282	0.02988719	-0.8304574
ENSG00000130595.19_TNNT3	TNNT3	troponin T3, fast skeletal type	HGNC:11950	11p15.5	1.00953921	12.6792326	3.79878685	0.00114344	0.02976707	-0.8305181
ENSG00000179085.7_DPM3	DPM3	dolichyl-phosphate mannosyltransferase subunit 3, regulatory	HGNC:3007	1q22	0.55896365	4.17222514	3.82068085	0.00108674	0.02904746	-0.8307668
ENSG00000174059.16_CD34	CD34	CD34 molecule	HGNC:1662	1q32.2	-0.6929163	5.50945309	-3.8298656	0.00106379	0.028804	-0.836515
ENSG00000079819.19_EPB41L2	EPB41L2	erythrocyte membrane protein band 4.1 like 2	HGNC:3379	6q23.1-q23.2	-0.8380853	4.26695389	-3.8220763	0.00108322	0.02900515	-0.8375823
ENSG00000205215.8_KRT17P7	KRT17P7	keratin 17 pseudogene 7	HGNC:50725	17p11.2	2.71100743	-0.0761078	3.91038889	0.00088219	0.02598905	-0.8390784
ENSG00000149084.13_HSD17B12	HSD17B12	hydroxysteroid 17-beta dehydrogenase 12	HGNC:18646	11p11.2	-0.5699128	3.40142873	-3.8006581	0.00113848	0.02976004	-0.8430126
ENSG00000165731.19_RET	RET	ret proto-oncogene	HGNC:9967	10q11.21	-1.5826658	1.5705106	-3.7524557	0.00127329	0.03118699	-0.8448584
ENSG00000109787.13_KLF3	KLF3	Kruppel like factor 3	HGNC:16516	4p14	-0.6016255	3.10010975	-3.7903769	0.00116599	0.02988719	-0.8452048
ENSG00000019144.19_PHLDB1	PHLDB1	pleckstrin homology like domain family B member 1	HGNC:23697	11q23.3	-0.7056089	5.92546496	-3.8241824	0.00107793	0.02900515	-0.8495919
ENSG00000171488.15_LRRC8C	LRRC8C	leucine rich repeat containing 8 VRAC subunit C	HGNC:25075	1p22.2	-0.6662397	1.70564487	-3.752407	0.00127344	0.03118699	-0.8513397
ENSG00000137491.14_SLCO2B1	SLCO2B1	solute carrier organic anion transporter family member 2B1	HGNC:10962	11q13.4	-1.2836653	2.56691001	-3.7618333	0.00124588	0.03077377	-0.8645768
ENSG00000112394.17_SLC16A10	SLC16A10	solute carrier family 16 member 10	HGNC:17027	6q21	0.72337944	4.7887064	3.8095046	0.00111532	0.02954816	-0.8733445
ENSG00000100345.21_MYH9	MYH9	myosin heavy chain 9	HGNC:7579	22q12.3	-1.0403566	7.16885331	-3.8105471	0.00111263	0.02954816	-0.8738918
ENSG00000085276.18_MECOM	MECOM	MDS1 and EVI1 complex locus	HGNC:3498	3q26.2	-0.8808853	1.26420187	-3.7383075	0.00131579	0.03177176	-0.8743043
ENSG00000118096.7_IFT46	IFT46	intraflagellar transport 46	HGNC:26146	11q23.3	0.52258077	3.37004498	3.78129073	0.00119085	0.03011981	-0.8758021
ENSG00000130309.11_COLGALT1	COLGALT1	collagen beta(1-O)galactosyltransferase 1	HGNC:26182	19p13.11	-0.6145614	4.17874821	-3.8003547	0.00113928	0.02976004	-0.8767033
ENSG00000100319.12_ZMAT5	ZMAT5	zinc finger matrin-type 5	HGNC:28046	22q12.2	0.45699727	3.43062903	3.78123113	0.00119102	0.03011981	-0.876997
ENSG00000090776.6_EFNB1	EFNB1	ephrin B1	HGNC:3226	Xq13.1	-0.6903653	2.89231509	-3.767421	0.00122982	0.0305824	-0.8785291
ENSG00000204138.13_PHACTR4	PHACTR4	phosphatase and actin regulator 4	HGNC:25793	1p35.3	-0.5300505	3.65065908	-3.7879185	0.00117266	0.030007	-0.8801131
ENSG00000197283.16_SYNGAP1	SYNGAP1	synaptic Ras GTPase activating protein 1	HGNC:11497	6p21.32	-0.8957342	1.60851058	-3.7352609	0.00132512	0.03184331	-0.8817132
ENSG00000107566.14_ERLIN1	ERLIN1	ER lipid raft associated 1	HGNC:16947	10q24.31	-0.6022481	2.33136202	-3.7479604	0.00128665	0.03118699	-0.8857188
ENSG00000100403.12_ZC3H7B	ZC3H7B	zinc finger CCCH-type containing 7B	HGNC:30869	22q13.2	-0.5486225	5.32094738	-3.8064455	0.00112328	0.02965409	-0.8874764
ENSG00000163359.15_COL6A3	COL6A3	collagen type VI alpha 3 chain	HGNC:2213	2q37.3	-0.9770874	6.04702337	-3.8034214	0.00113119	0.02970623	-0.894858
ENSG00000132792.19_CTNNBL1	CTNNBL1	catenin beta like 1	HGNC:15879	20q11.23	0.52405197	3.95463846	3.78691926	0.00117539	0.03002548	-0.8969285
ENSG00000141504.11_SAT2	SAT2	spermidine/spermine N1-acetyltransferase family member 2	HGNC:23160	17p13.1	0.43038142	5.17167327	3.79963659	0.00114118	0.02976004	-0.9018476
ENSG00000197859.10_ADAMTSL2	ADAMTSL2	ADAMTS like 2	HGNC:14631	9q34.2	-1.1239728	2.49948538	-3.7374027	0.00131856	0.03178734	-0.9067757
ENSG00000176435.6_CLEC14A	CLEC14A	C-type lectin domain containing 14A	HGNC:19832	14q21.1	-0.6937824	3.94760201	-3.7831135	0.00118582	0.03011981	-0.9075593
ENSG00000178982.9_EIF3K	EIF3K	eukaryotic translation initiation factor 3 subunit K	HGNC:24656	19q13.2	0.54824437	7.44205868	3.79388925	0.00115652	0.02988719	-0.9111803
ENSG00000161921.15_CXCL16	CXCL16	C-X-C motif chemokine ligand 16	HGNC:16642	17p13.2	-1.2651167	0.30836656	-3.7487409	0.00128432	0.03118699	-0.9159115
ENSG00000160050.14_CCDC28B	CCDC28B	coiled-coil domain containing 28B	HGNC:28163	1p35.2	0.7404391	5.7861758	3.7928691	0.00115926	0.02988719	-0.9188374
ENSG00000052850.7_ALX4	ALX4	ALX homeobox 4	HGNC:450	11p11.2	-2.2487723	-1.5200613	-4.086259	0.00058594	0.02051241	-0.9239198
ENSG00000167985.6_SDHAF2	SDHAF2	succinate dehydrogenase complex assembly factor 2	HGNC:26034	11q12.2	0.55661516	3.77780787	3.76953513	0.0012238	0.03053395	-0.9249938
ENSG00000106153.13_CHCHD2	CHCHD2	coiled-coil-helix-coiled-coil-helix domain containing 2	HGNC:21645	7p11.2	0.47010836	7.90811346	3.78377047	0.00118401	0.03011981	-0.9290166
ENSG00000100142.15_POLR2F	POLR2F	RNA polymerase II subunit F	HGNC:9193	22q13.1	0.48871796	4.59000936	3.78118881	0.00119113	0.03011981	-0.9324383
ENSG00000133574.10_GIMAP4	GIMAP4	GTPase, IMAP family member 4	HGNC:21872	7q36.1	-0.6111388	3.13455805	-3.7470079	0.00128949	0.03118699	-0.9371346
ENSG00000114554.11_PLXNA1	PLXNA1	plexin A1	HGNC:9099	3q21.3	-0.6680089	4.71449084	-3.7800938	0.00119417	0.0301316	-0.9381005
ENSG00000136960.12_ENPP2	ENPP2	ectonucleotide pyrophosphatase/phosphodiesterase 2	HGNC:3357	8q24.12	-1.226088	1.71649068	-3.7053282	0.00142039	0.03290341	-0.9443614
ENSG00000091317.8_CMTM6	CMTM6	CKLF like MARVEL transmembrane domain containing 6	HGNC:19177	3p22.3	-0.5944472	2.13071328	-3.7137599	0.00139289	0.03258401	-0.946787
ENSG00000138615.6_CILP	CILP	cartilage intermediate layer protein	HGNC:1980	15q22.31	-1.3686505	5.15956412	-3.7762973	0.00120474	0.03025973	-0.9491885

ENSG00000103363.14_ELOB	ELOB	elongin B	HGNC:11619	16p13.3	0.7681005	6.79437925	3.77804474	0.00119986	0.03018777	-0.949598
ENSG00000130770.18_ATP5IF1	ATP5IF1	ATP synthase inhibitory factor subunit 1	HGNC:871	1p35.3	0.64461605	7.13678669	3.77449315	0.0012098	0.03033593	-0.9560231
ENSG00000167085.11_PHB	PHB	prohibitin	HGNC:8912	17q21.33	0.44665498	6.45918265	3.77221618	0.00121621	0.0304458	-0.9635171
ENSG00000161920.10_MED11	MED11	mediator complex subunit 11	HGNC:32687	17p13.2	0.50147419	3.096247	3.73156001	0.00133655	0.0318635	-0.9651599
ENSG00000175220.12_ARHGAP1	ARHGAP1	Rho GTPase activating protein 1	HGNC:673	11p11.2	-0.7764755	3.89832703	-3.7544399	0.00126744	0.03115622	-0.9668157
ENSG00000261087.1_AP003469.4	AP003469.4				0.68126471	3.20000569	3.73177874	0.00133587	0.0318635	-0.9670749
ENSG00000103335.22_PIEZO1	PIEZO1	piezo type mechanosensitive ion channel component 1	HGNC:28993	16q24.3	-1.1468034	3.65919546	-3.7471421	0.00128909	0.03118699	-0.968514
ENSG00000254999.4_BRK1	BRK1	BRICK1 subunit of SCAR/WAVE actin nucleating complex	HGNC:23057	3p25.3	0.45939641	6.14756621	3.76834938	0.00122718	0.03056725	-0.9727633
ENSG00000129244.9_ATP1B2	ATP1B2	ATPase Na+/K+ transporting subunit beta 2	HGNC:805	17p13.1	-1.1978975	0.06240838	-3.7365178	0.00132127	0.03180155	-0.9761618
ENSG00000073282.13_TP63	TP63	tumor protein p63	HGNC:15979	3q28	0.95766358	4.80930098	3.76180448	0.00124596	0.03077377	-0.9764587
ENSG00000143575.14_HAX1	HAX1	HCLS1 associated protein X-1	HGNC:16915	1q21.3	0.44446074	6.5859701	3.76574166	0.00123463	0.03065102	-0.9774459
ENSG00000185437.13_SH3BGR	SH3BGR	SH3 domain binding glutamate rich protein	HGNC:10822	21q22.2	0.55873872	7.98036282	3.76127854	0.00124748	0.03077377	-0.9775617
ENSG00000145741.15_BTF3	BTF3	basic transcription factor 3	HGNC:1125	5q13.2	0.55662599	7.69354243	3.76113283	0.00124791	0.03077377	-0.9808262
ENSG00000179104.9_TMTC2	TMTC2	transmembrane O-mannosyltransferase targeting cadherins 2	HGNC:25440	12q21.31	-1.3098352	-0.2410937	-3.7604683	0.00124983	0.03077377	-0.9830323
ENSG00000163590.14_PPM1L	PPM1L	protein phosphatase, Mg2+/Mn2+ dependent 1L	HGNC:16381	3q25.33-q26.	0.61707173	4.13392351	3.75097627	0.00127767	0.03118699	-0.9830738
ENSG00000158813.18_EDA	EDA	ectodysplasin A	HGNC:3157	Xq13.1	-0.990205	0.48295954	-3.703422	0.00142668	0.03291245	-0.9855251
ENSG00000280153.1_AC133065.3	AC133065.3				-2.5696122	-1.9848526	-4.0489903	0.00063903	0.02124657	-0.9899037
ENSG00000136802.11_LRRC8A	LRRC8A	leucine rich repeat containing 8 VRAC subunit A	HGNC:19027	9q34.11	-0.6015152	3.35014028	-3.7306599	0.00133934	0.0318635	-0.9907248
ENSG00000182240.16_BACE2	BACE2	beta-secretase 2	HGNC:934	21q22.2-q22.	-0.5527742	2.22919476	-3.694901	0.00145515	0.03326207	-0.9929654
ENSG00000269113.4_TRABD2B	TRABD2B	TraB domain containing 2B	HGNC:44200	1p33	-1.406886	-0.5311833	-3.8004118	0.00113913	0.02976004	-1.0088825
ENSG00000213366.13_GSTM2	GSTM2	glutathione S-transferase mu 2	HGNC:4634	1p13.3	0.57854873	6.8763441	3.75036839	0.00127948	0.03118699	-1.0102687
ENSG00000129467.13_ADCY4	ADCY4	adenylate cyclase 4	HGNC:235	14q12	-0.8507954	3.48939555	-3.7233793	0.00136216	0.03215636	-1.0112259
ENSG00000140319.10_SRP14	SRP14	signal recognition particle 14	HGNC:11299	15q22	0.44199567	7.06729628	3.74898525	0.00128359	0.03118699	-1.0120759
ENSG00000131100.13_ATP6V1E1	ATP6V1E1	ATPase H+ transporting V1 subunit E1	HGNC:857	22q11.21	0.44382292	6.17412921	3.74951146	0.00128202	0.03118699	-1.0140887
ENSG00000147687.19_TATDN1	TATDN1	TatD DNase domain containing 1	HGNC:24220	8q24.13	0.54996808	3.78569633	3.72881503	0.00134509	0.0318635	-1.0151965
ENSG00000114790.13_ARHGEF26	ARHGEF26	Rho guanine nucleotide exchange factor 26	HGNC:24490	3q25.2	-1.3017258	-1.2476631	-3.8850552	0.00093572	0.02661296	-1.0158807
ENSG00000105137.13_SYDE1	SYDE1	synapse defective Rho GTPase homolog 1	HGNC:25824	19p13.12	-0.8629963	1.87191903	-3.6711862	0.00153736	0.0344585	-1.0220996
ENSG00000143772.9_ITPKB	ITPKB	inositol-trisphosphate 3-kinase B	HGNC:6179	1q42.12	-0.7184662	3.76396592	-3.7249835	0.0013571	0.03209726	-1.0269631
ENSG00000129460.16_NGDN	NGDN	neuroguidin	HGNC:20271	14q11.2	0.52050271	3.77860486	3.71758587	0.00138058	0.03244803	-1.0386702
ENSG00000119801.13_YPEL5	YPEL5	yippee like 5	HGNC:18329	2p23.1	0.38786211	4.70633168	3.73262269	0.00133326	0.0318635	-1.0425202
ENSG00000140577.16_CRTC3	CRTC3	CREB regulated transcription coactivator 3	HGNC:26148	15q26.1	-0.5029952	3.70017631	-3.7139996	0.00139211	0.03258401	-1.0481479
ENSG00000181982.18_CCDC149	CCDC149	coiled-coil domain containing 149	HGNC:25405	4p15.2	-0.5284132	2.22047173	-3.6656478	0.00155722	0.03464457	-1.0524223
ENSG00000241370.5_RPP21	RPP21	ribonuclease P/MRP subunit p21	HGNC:21300	6p22.1	0.5881849	3.2398346	3.69411748	0.00145779	0.03327182	-1.0530798
ENSG00000168899.5_VAMP5	VAMP5	vesicle associated membrane protein 5	HGNC:12646	2p11.2	0.54944092	5.91404457	3.73173948	0.00133599	0.0318635	-1.0533825
ENSG00000126088.14_UROD	UROD	uroporphyrinogen decarboxylase	HGNC:12591	1p34.1	0.41385766	5.4882489	3.73022354	0.0013407	0.0318635	-1.0561693
ENSG00000177156.11_TALDO1	TALDO1	transaldolase 1	HGNC:11559	11p15.5	0.51143477	5.54789432	3.72948256	0.00134301	0.0318635	-1.0579038
ENSG00000137818.12_RPLP1	RPLP1	ribosomal protein lateral stalk subunit P1	HGNC:10372	15q23	0.58415571	10.389532	3.70599384	0.0014182	0.03290341	-1.0600566
ENSG00000266028.7_SRGAP2	SRGAP2	SLIT-ROBO Rho GTPase activating protein 2	HGNC:19751	1q32.1	-0.7086082	2.64111419	-3.6747053	0.00152488	0.03441982	-1.0620657
ENSG00000136048.14_DRAM1	DRAM1	DNA damage regulated autophagy modulator 1	HGNC:25645	12q23.2	-0.8995237	1.13947478	-3.6437992	0.00163804	0.03538318	-1.064514
ENSG00000149480.7_MTA2	MTA2	metastasis associated 1 family member 2	HGNC:7411	11q12.3	-0.5215445	3.56194787	-3.699685	0.0014391	0.03309702	-1.0669695
ENSG00000143515.17_ATP8B2	ATP8B2	ATPase phospholipid transporting 8B2	HGNC:13534	1q21.3	-0.9261875	2.33774749	-3.6618834	0.00157085	0.03474183	-1.0684186
ENSG00000120699.13_EXOSC8	EXOSC8	exosome component 8	HGNC:17035	13q13.3	0.47906416	3.76867193	3.70122207	0.00143398	0.03302994	-1.0739905
ENSG00000197312.12_DDI2	DDI2	DNA damage inducible 1 homolog 2	HGNC:24578	1p36.21	0.68970649	5.61516236	3.72069093	0.00137068	0.03226576	-1.0772151
ENSG00000103351.13_CLUAP1	CLUAP1	clusterin associated protein 1	HGNC:19009	16p13.3	0.57373916	2.87093385	3.66914431	0.00154465	0.03451871	-1.0796855
ENSG00000155660.11_PDIA4	PDIA4	protein disulfide isomerase family A member 4	HGNC:30167	7q36.1	-0.5705871	3.0001994	-3.6766837	0.0015179	0.03433023	-1.0802892
ENSG00000169604.19_ANTXR1	ANTXR1	ANTXR cell adhesion molecule 1	HGNC:21014	2p13.3	-0.8190603	3.432353	-3.6918122	0.0014656	0.03334907	-1.0817933
ENSG00000083444.17_PLOD1	PLOD1	procollagen-lysine,2-oxoglutarate 5-dioxygenase 1	HGNC:9081	1p36.22	-0.469849	4.75128444	-3.7148083	0.00138951	0.03258401	-1.0826921
ENSG00000132514.13_CLEC10A	CLEC10A	C-type lectin domain containing 10A	HGNC:16916	17p13.1	-1.7606527	-0.1710706	-3.7039873	0.00142481	0.03291245	-1.0838875
ENSG00000147119.3_CHST7	CHST7	carbohydrate sulfotransferase 7	HGNC:13817	Xp11.3	-1.0219252	0.87038491	-3.6352012	0.00167098	0.03568143	-1.090764
ENSG00000285336.1_AC108734.4	AC108734.4				1.04589232	1.53501657	3.62926672	0.00169409	0.03593052	-1.093659
ENSG00000108557.19_RAI1	RAI1	retinoic acid induced 1	HGNC:9834	17p11.2	-0.6623367	3.40491238	-3.6792278	0.00150898	0.03417998	-1.0962252
ENSG00000105825.13_TFPI2	TFPI2	tissue factor pathway inhibitor 2	HGNC:11761	7q21.3	-1.6384159	-0.9068573	-3.8497013	0.00101587	0.02789524	-1.1003373
ENSG00000137100.16_DCTN3	DCTN3	dynactin subunit 3	HGNC:2713	9p13.3	0.4177137	5.549703	3.71017434	0.00140452	0.0328049	-1.1004163
ENSG00000074317.11_SNCB	SNCB	synuclein beta	HGNC:11140	5q35.2	1.68762084	-0.91817	3.86860066	0.00097221	0.02702418	-1.101322
ENSG00000161940.10_BCL6B	BCL6B	BCL6B transcription repressor	HGNC:1002	17p13.1	-1.1268986	2.08037399	-3.6341091	0.00167521	0.03568143	-1.1040121
ENSG00000167325.15_RRM1	RRM1	ribonucleotide reductase catalytic subunit M1	HGNC:10451	11p15.4	0.43062514	4.76767686	3.70498349	0.00142153	0.03290341	-1.1046244
ENSG00000053371.12_AKR7A2	AKR7A2	aldo-keto reductase family 7 member A2	HGNC:389	1p36.13	0.61502041	4.9180595	3.70486961	0.0014219	0.03290341	-1.1053277
ENSG00000156011.17_PSD3	PSD3	pleckstrin and Sec7 domain containing 3	HGNC:19093	8p22	-0.9554814	0.0912575	-3.6644696	0.00156147	0.0346878	-1.106102
ENSG00000135972.9_MRPS9	MRPS9	mitochondrial ribosomal protein S9	HGNC:14501	2q12.1	0.56533487	5.47206957	3.707349	0.00141375	0.03290341	-1.1062925
ENSG00000146425.11_DYNLT1	DYNLT1	dynein light chain Tctex-type 1	HGNC:11697	6q25.3	0.74613338	4.51437626	3.69822617	0.00144397	0.03315829	-1.1113707
ENSG00000125352.5_RNF113A	RNF113A	ring finger protein 113A	HGNC:12974	Xq24	0.49148613	2.501936	3.64205111	0.00164469	0.03538318	-1.1133373
ENSG00000196419.12_XRCC6	XRCC6	X-ray repair cross complementing 6	HGNC:4055	22q13.2	0.38348199	7.36285893	3.69727506	0.00144716	0.03318067	-1.1236937
ENSG00000228049.7_POLR2J2	POLR2J2	RNA polymerase II subunit J2	HGNC:23208	7q22.1	1.59470501	1.85405722	3.6142979	0.0017538	0.0366271	-1.1246377
ENSG00000169371.13_SNUPN	SNUPN	snurportin 1	HGNC:14245	15q24.2	0.34739124	3.97208022	3.6837109	0.00149338	0.03392897	-1.1257841
ENSG00000076356.7_PLXNA2	PLXNA2	plexin A2	HGNC:9100	1q32.2	-0.5538002	3.96141906	-3.6830608	0.00149564	0.03392897	-1.1281468
ENSG00000090006.17_LTBP4	LTBP4	latent transforming growth factor beta binding protein 4	HGNC:6717	19q13.2	-1.0688779	6.21357558	-3.6964624	0.00144989	0.03319249	-1.1291338
ENSG00000114021.12_NIT2	NIT2	nitrilase family member 2	HGNC:29878	3q12.2	0.43029245	4.81798669	3.69180583	0.00146562	0.03334907	-1.1334231
ENSG00000021762.20_OSBPL5	OSBPL5	oxysterol binding protein like 5	HGNC:16392	11p15.4	-0.7167308	2.6788565	-3.6360505	0.0016677	0.03567344	-1.1408651
ENSG00000138028.16_CGREF1	CGREF1	cell growth regulator with EF-hand domain 1	HGNC:16962	2p23.3	1.16270123	0.74326704	3.6187915	0.00173566	0.03642773	-1.1461353
ENSG00000205312.8_KRT17P4	KRT17P4	keratin 17 pseudogene 4	HGNC:50722	17p11.2	2.6829519	0.07783796	3.72283252	0.00136388	0.03215636	-1.1461755
ENSG00000183671.12_GPR1	GPR1	G protein-coupled receptor 1	HGNC:4463	2q33.3	-1.9579613	-0.9735618	-3.7933186	0.00115805	0.02988719	-1.1473732
ENSG00000049541.11_RFC2	RFC2	replication factor C subunit 2	HGNC:9970	7q11.23	0.424562	3.21124866	3.64888097	0.00161888	0.03518132	-1.1490087
ENSG00000285796.1_AL162458.1	AL162458.1				-1.0372951	1.3424495	-3.5989545	0.00181717	0.03742914	-1.1554397
ENSG00000182004.13_SNRPE	SNRPE	small nuclear ribonucleoprotein polypeptide E	HGNC:11161	1q32.1	0.55295146	3.96803222	3.66767945	0.0015499	0.03458436	-1.1564941
ENSG00000164402.14_SEPT8	SEPT8	septin-8			-0.6029271	2.94623925	-3.6391536	0.00165576	0.0355542	-1.1628646
ENSG00000137154.12_RPS6	RPS6	ribosomal protein S6	HGNC:10429	9p22.1	0.47690711	8.60997679	3.67181483	0.00153512	0.0344585	-1.1633043
ENSG00000120690.16_ELF1	ELF1	E74 like ETS transcription factor 1	HGNC:3316	13q14.11	-0.581388	3.05797785	-3.6425607	0.00164275	0.03538318	-1.1652771
ENSG00000169744.13_LDB2	LDB2	LIM domain binding 2	HGNC:6533	4p15.32	-0.510723	2.51243851	-3.622667	0.00172017	0.0362783	-1.1675142
ENSG00000024422.12_EHD2	EHD2	EH domain containing 2	HGNC:3243	19q13.33	-1.1338117	4.71845469	-3.6732006	0.0015302	0.03445253	-1.17003
ENSG00000178996.14_SNX18	SNX18	sorting nexin 18	HGNC:19245	5q11.2	-0.4113708	3.09970018	-3.6387309	0.00165738	0.0355542	-1.1700918
ENSG00000109586.12_GALNT7	GALNT7	polypeptide N-acetylgalactosaminyltransferase 7	HGNC:4129	4q34.1	-1.3077699	-0.3184838	-3.6670396	0.0015522	0.03458436	-1.171842
ENSG00000237440.9_AC008554.1	AC008554.1				-0.9667562	0.11395349	-3.6324111	0.0016818	0.03577115	-1.176936
ENSG00000142507.10_PSMB6	PSMB6	proteasome 20S subunit beta 6	HGNC:9543	17p13.2	0.46933771	6.74431951	3.67199373	0.00153449	0.0344585	-1.1825794
ENSG00000183172.8_SMDT1	SMDT1	single-pass membrane protein with aspartate rich tail 1	HGNC:25055	22q13.2	0.47159103	5.97931003	3.67000884	0.00154156	0.03450106	-1.1887276
ENSG00000136068.14_FLNB	FLNB	filamin B	HGNC:3755	3p14.3	-0.8811448	4.59203978	-3.6625841	0.00156831	0.03473672	-1.1922612
ENSG00000203685.10_STUM	STUM	stum, mechanosensory transduction mediator homolog	HGNC:30491	1q42.12	-0.9099734	1.57114455	-3.5840135	0.00188104	0.03839958	-1.1932875
ENSG00000153208.16_MERTK	MERTK	MER proto-oncogene, tyrosine kinase	HGNC:7027	2q13	-1.3770624	-0.1006323	-3.6237035	0.00171604	0.03624236	-1.194355
ENSG00000008256.16_CYTH3	CYTH3	cytohesin 3	HGNC:9504	7p22.1	-0.5847063	4.13052272	-3.654538	0.00159781	0.03503191	-1.1976538
ENSG00000126226.21_PCID2	PCID2	PCI domain containing 2	HGNC:25653	13q34	0.47323509	4.27296555	3.6545164	0.00159789	0.03503191	-1.199554
ENSG00000156411.9_ATP5MPL	ATP5MPL	ATP synthase membrane subunit 6.8PL	HGNC:1188	14q32.33	0.66021382	6.68110727	3.66348746	0.00156503	0.03471538	-1.2013367
ENSG00000131171.13_SH3BGRL	SH3BGRL	SH3 domain binding glutamate rich protein like	HGNC:10823	Xq21.1	-0.7611125	2.84852233	-3.616203	0.00174609	0.03651687	-1.2022095
ENSG00000088827.12_SIGLEC1	SIGLEC1	sialic acid binding Ig like lectin 1	HGNC:11127	20p13	-1.5103987	1.94230342	-3.5780223	0.00190727	0.03870037	-1.2100802
ENSG00000168028.14_RPSA	RPSA	ribosomal protein SA	HGNC:6502	3p22.1	0.40259243	8.16620223	3.65114471	0.00161042	0.0351502	-1.2161864
ENSG00000106537.8_TSPAN13	TSPAN13	tetraspanin 13	HGNC:21643	7p21.1	-0.6435852	1.86173216	-3.5732159	0.00192858	0.03871358	-1.2186513
ENSG00000174640.13_SLCO2A1	SLCO2A1	solute carrier organic anion transporter family member 2A1	HGNC:10955	3q22.1-q22.2	-1.2488826	-0.434716	-3.6195771	0.00173251	0.03642773	-1.2206459
ENSG00000213762.12_ZNF134	ZNF134	zinc finger protein 134	HGNC:12918	19q13.43	0.48089583	3.24846734	3.61786236	0.0017394	0.03642773	-1.2209932
ENSG00000060982.15_BCAT1	BCAT1	branched chain amino acid transaminase 1	HGNC:976	12p12.1	-1.3890314	-0.0721104	-3.6346008	0.0016733	0.03568143	-1.2210813
ENSG00000168032.9_ENTPD3	ENTPD3	ectonucleoside triphosphate diphosphohydrolase 3	HGNC:3365	3p22.1	-1.6381896	-1.3264267	-3.7956547	0.00115179	0.02988064	-1.2220657
ENSG00000041357.16_PSMA4	PSMA4	proteasome 20S subunit alpha 4	HGNC:9533	15q25.1	0.47537813	6.17127818	3.65448983	0.00159799	0.03503191	-1.2224331
ENSG00000114942.14_EEF1B2	EEF1B2	eukaryotic translation elongation factor 1 beta 2	HGNC:3208	2q33.3	0.45730318	7.99247864	3.64917534	0.00161778	0.03518132	-1.2229605
ENSG00000213178.3_RPL22P1	RPL22P1	ribosomal protein L22 pseudogene 1	HGNC:17998	3q26.2	1.01813682	-0.2217215	3.65554018	0.00159411	0.03503191	-1.2262906

ENSG00000127922.9_SEM1	SEM1	SEM1 26S proteasome complex subunit	HGNC:10845	7q21.3	0.54537583	5.33551729	3.65157309	0.00160882	0.0351502	-1.2277263
ENSG00000106588.11_PSMA2	PSMA2	proteasome 20S subunit alpha 2	HGNC:9531	7p14.1	0.49583619	5.85545577	3.65189783	0.00160761	0.0351502	-1.2284325
ENSG00000038382.20_TRIO	TRIO	trio Rho guanine nucleotide exchange factor	HGNC:12303	5p15.2	-0.5905899	5.01859016	-3.6495042	0.00161655	0.03518132	-1.2294972
ENSG00000256646.7_AC010132.3	AC010132.3				2.5991209	1.37281368	3.56958927	0.00194481	0.03885449	-1.2308256
ENSG00000102898.12_NUTF2	NUTF2	nuclear transport factor 2	HGNC:13722	16q22.1	0.42741618	5.24204284	3.64694261	0.00162616	0.0352399	-1.2373687
ENSG00000124570.19_SERPINB6	SERPINB6	serpin family B member 6	HGNC:8950	6p25.2	0.43491763	6.05500984	3.64691264	0.00162628	0.0352399	-1.2391451
ENSG00000124126.14_PREX1	PREX1	phosphatidylinositol-3,4,5-trisphosphate dependent Rac exch	HGNC:32594	20q13.13	-0.7087867	3.50992325	-3.6180943	0.00173846	0.03642773	-1.2396047
ENSG00000161896.12_IP6K3	IP6K3	inositol hexakisphosphate kinase 3	HGNC:17269	6p21.31	0.51632226	5.80120684	3.6455246	0.00163151	0.03530234	-1.2423098
ENSG00000253636.1_AC022893.1	AC022893.1				0.79745672	0.50089824	3.574492	0.0019229	0.03871358	-1.2423234
ENSG00000159352.15_PSMD4	PSMD4	proteasome 26S subunit, non-ATPase 4	HGNC:9561	1q21.3	0.53857623	7.07735619	3.64240012	0.00164336	0.03538318	-1.2457292
ENSG00000119878.5_CRIPT	CRIPT	CXXC repeat containing interactor of PDZ3 domain	HGNC:14312	2p21	0.56256902	3.30866483	3.6076176	0.00178112	0.03694032	-1.2469061
ENSG00000145982.12_FARS2	FARS2	phenylalanyl-tRNA synthetase 2, mitochondrial	HGNC:21062	6p25.1	0.47540868	3.4122741	3.61066207	0.00176862	0.03673187	-1.2474072
ENSG00000168952.15_STXBP6	STXBP6	syntaxin binding protein 6	HGNC:19666	14q11.2	-0.8334809	0.77089705	-3.5525678	0.00202281	0.03973999	-1.24991
ENSG00000173660.12_UQCRH	UQCRH	ubiquinol-cytochrome c reductase hinge protein	HGNC:12590	1p33	0.64287307	7.15382196	3.63696932	0.00166415	0.0356485	-1.2570222
ENSG00000162654.9_GBP4	GBP4	guanylate binding protein 4	HGNC:20480	1p22.2	-0.9928725	2.93611647	-3.5829959	0.00188547	0.03841394	-1.263182
ENSG00000102302.8_FGD1	FGD1	FYVE, RhoGEF and PH domain containing 1	HGNC:3663	Xp11.22	-1.4470776	-0.6184257	-3.6742591	0.00152645	0.03441982	-1.265356
ENSG00000136114.17_THSD1	THSD1	thrombospondin type 1 domain containing 1	HGNC:17754	13q14.3	-0.9103713	1.63320252	-3.5466681	0.00205056	0.03997699	-1.2656961
ENSG00000116898.12_MRPS15	MRPS15	mitochondrial ribosomal protein S15	HGNC:14504	1p34.3	0.50012001	6.3700784	3.63159354	0.00168499	0.03578814	-1.2720781
ENSG00000094963.14_FMO2	FMO2	flavin containing dimethylaniline monoxygenase 2	HGNC:3770	1q24.3	-0.9601473	3.11352201	-3.5949727	0.00183398	0.03772181	-1.2745338
ENSG00000198890.8_PRMT6	PRMT6	protein arginine methyltransferase 6	HGNC:18241	1p13.3	-0.6483038	0.94727666	-3.5390557	0.00208692	0.04001704	-1.2787517
ENSG00000169020.10_ATP5ME	ATP5ME	ATP synthase membrane subunit e	HGNC:846	4p16.3	0.78419438	6.5098115	3.62832677	0.00169778	0.03595791	-1.2788339
ENSG00000170340.11_B3GNT2	B3GNT2	UDP-GlcNAc:betaGal beta-1,3-N-acetylglucosaminyltransferas	HGNC:15629	2p15	-0.9531465	0.55999191	-3.5450638	0.00205817	0.03997699	-1.2808631
ENSG00000076108.11_BAZ2A	BAZ2A	bromodomain adjacent to zinc finger domain 2A	HGNC:962	12q13.3	-0.4753425	5.1175468	-3.6256957	0.00170815	0.03612652	-1.282659
ENSG00000133110.15_POSTN	POSTN	periostin	HGNC:16953	13q13.3	-1.7769362	0.97818778	-3.5331071	0.00211577	0.04026143	-1.2868681
ENSG00000170004.16_CHD3	CHD3	chromodomain helicase DNA binding protein 3	HGNC:1918	17p13.1	-0.8851593	3.76724468	-3.6023062	0.00180314	0.0371912	-1.2900801
ENSG00000181007.8_ZFP82	ZFP82	ZFP82 zinc finger protein	HGNC:28682	19q13.12	0.88296839	0.01397242	3.58006704	0.00189828	0.03857009	-1.2956973
ENSG00000272414.6_FAM47E-STBD1	FAM47E-STBD1	FAM47E-STBD1 readthrough	HGNC:44667	4q21.1	0.95895833	4.57604423	3.61244092	0.00176135	0.03668262	-1.2974373
ENSG00000143570.18_SLC39A1	SLC39A1	solute carrier family 39 member 1	HGNC:12876	1q21.3	-0.3946442	4.36237402	-3.6112287	0.0017663	0.03673187	-1.2989215
ENSG00000245910.8_SNHG6	SNHG6	small nucleolar RNA host gene 6	HGNC:32965	8q13.1	0.5421526	6.42032093	3.61846009	0.00173699	0.03642773	-1.3007286
ENSG00000134013.15_LOXL2	LOXL2	lysyl oxidase like 2	HGNC:6666	8p21.3	-1.2732017	1.66505405	-3.5287452	0.00213718	0.04056584	-1.3037047
ENSG00000182963.10_GJC1	GJC1	gap junction protein gamma 1	HGNC:4280	17q21.31	-0.8307326	2.0876495	-3.5377308	0.00209331	0.04003776	-1.3090434
ENSG00000167526.13_RPL13	RPL13	gap junction protein delta 3	HGNC:19147	17q21.2	0.54748989	9.54258348	3.59440021	0.00183641	0.03772181	-1.3159082
ENSG00000126749.16_EMG1	EMG1	ribosomal protein L13	HGNC:10303	16q24.3	0.45079029	4.52570043	3.60459523	0.00179361	0.03708041	-1.3162851
ENSG00000260260.1_SNHG19	SNHG19	EMG1 N1-specific pseudouridine methyltransferase	HGNC:16912	12p13.31	0.86862379	2.61940697	3.54850124	0.0020419	0.03991387	-1.3163166
ENSG00000254539.1_AC239804.1	AC239804.1	small nucleolar RNA host gene 19	HGNC:49574	16p13.3	0.84534344	3.20528351	3.56802245	0.00195186	0.03892048	-1.3191788
ENSG00000108387.14_SEPT4	SEPT4	septin-4			-0.5554594	2.15346901	-3.5364303	0.0020996	0.04005835	-1.3197854
ENSG00000077713.19_SLC25A43	SLC25A43	solute carrier family 25 member 43	HGNC:30557	Xq24	-1.0987835	0.10976398	-3.5523298	0.00202392	0.03973999	-1.3199732
ENSG00000157240.3_FZD1	FZD1	frizzled class receptor 1	HGNC:4038	7q21.13	-0.67504	2.3892884	-3.5436538	0.00206488	0.04001704	-1.3211334
ENSG00000154124.4_OTULIN	OTULIN	OTU deubiquitinase with linear linkage specificity	HGNC:25118	5p15.2	0.4952086	2.77867472	3.55308454	0.0020204	0.03973999	-1.3218723
ENSG00000183853.18_KIRREL1	KIRREL1	kirre like nephrin family adhesion molecule 1	HGNC:15734	1q23.1	-1.0346957	2.54447206	-3.5459885	0.00205378	0.03997699	-1.3260624
ENSG00000034713.8_GABARAPL2	GABARAPL2	GABA type A receptor associated protein like 2	HGNC:13291	16q23.1	0.39477644	5.99326456	3.60622453	0.00178687	0.03700836	-1.3282166
ENSG00000091136.14_LAMB1	LAMB1	laminin subunit beta 1	HGNC:6486	7q31.1	-0.8203223	3.98233452	-3.5880306	0.00186365	0.03817689	-1.3359772
ENSG00000006118.14_TMEM132A	TMEM132A	transmembrane protein 132A	HGNC:31092	11q12.2	-1.0914835	1.14101365	-3.5072293	0.00224594	0.04183588	-1.3378356
ENSG00000230561.4_CCDC192	CCDC192	coiled-coil domain containing 192	HGNC:49566	5q23.2-q23.3	1.46603698	-0.6922611	3.66033149	0.00157651	0.0348156	-1.3427567
ENSG00000188739.15_RBM34	RBM34	RNA binding motif protein 34	HGNC:28965	1q42.3	0.44890126	3.75097753	3.57402323	0.00192498	0.03871358	-1.3528853
ENSG00000128524.5_ATP6V1F	ATP6V1F	ATPase H+ transporting V1 subunit F	HGNC:16832	7q32.1	0.49265592	5.1479467	3.59196974	0.00184676	0.03788251	-1.3565733
ENSG00000104529.17_EEF1D	EEF1D	eukaryotic translation elongation factor 1 delta	HGNC:3211	8q24.3	0.45961393	8.27782362	3.58374578	0.00188221	0.03839958	-1.3614852
ENSG00000140259.7_MFAP1	MFAP1	microfibril associated protein 1	HGNC:7032	15q15.3	0.50377607	4.08607004	3.57353752	0.00192715	0.03871358	-1.3689603
ENSG00000120333.4_MRPS14	MRPS14	mitochondrial ribosomal protein S14	HGNC:14049	1q25.1	0.48235223	3.53084206	3.55787456	0.00199817	0.03964419	-1.371463
ENSG00000152082.14_MZT2B	MZT2B	mitotic spindle organizing protein 2B	HGNC:25886	2q21.1	0.53928753	5.91112033	3.58603687	0.00187226	0.03830095	-1.3723031
ENSG00000230910.3_AL391807.1	AL391807.1				-1.7788711	-0.8762278	-3.6595998	0.00157919	0.03482337	-1.3748821
ENSG00000172336.5_POP7	POP7	POP7 homolog, ribonuclease P/MRP subunit	HGNC:19949	7q22.1	0.44005989	4.30697389	3.57408414	0.00192471	0.03871358	-1.375814
ENSG00000150712.11_MTMR12	MTMR12	myotubularin related protein 12	HGNC:18191	5p13.3	-0.5781516	2.66081714	-3.5259948	0.00215079	0.04061847	-1.3762461
ENSG00000224470.8_ATXN1L	ATXN1L	ataxin 1 like	HGNC:33279	16q22.2	-0.4890117	4.38589029	-3.5768111	0.00191262	0.03870411	-1.3765243
ENSG00000185973.11_TMLHE	TMLHE	trimethyllysine hydroxylase, epsilon	HGNC:18308	Xq28	0.59568822	4.57011572	3.57687718	0.00191233	0.03870411	-1.376526
ENSG00000116288.13_PARK7	PARK7	Parkinsonism associated deglycase	HGNC:16369	1p36.23	0.51866811	7.65645955	3.58027689	0.00189736	0.03857009	-1.3770625
ENSG00000130702.15_LAMA5	LAMA5	laminin subunit alpha 5	HGNC:6485	20q13.33	-0.9932167	4.55181454	-3.5734528	0.00192752	0.03871358	-1.3846271
ENSG00000160460.16_SPTBN4	SPTBN4	spectrin beta, non-erythrocytic 4	HGNC:14896	19q13.2	-0.6289731	2.91852397	-3.527336	0.00214414	0.04058444	-1.3891535
ENSG00000184924.5_PTRHD1	PTRHD1	peptidyl-tRNA hydrolase domain containing 1	HGNC:33782	2p23.3	0.54703327	3.65668389	3.55390395	0.00201658	0.03973999	-1.3894873
ENSG00000134871.18_COL4A2	COL4A2	collagen type IV alpha 2 chain	HGNC:2203	13q34	-1.1592723	5.685938	-3.572596	0.00193134	0.03871725	-1.4000095
ENSG00000115685.15_PPP1R7	PPP1R7	protein phosphatase 1 regulatory subunit 7	HGNC:9295	2q37.3	0.45622673	5.13222199	3.57150458	0.00193622	0.03876321	-1.4012165
ENSG00000115306.16_SPTBN1	SPTBN1	spectrin beta, non-erythrocytic 1	HGNC:11275	2p16.2	-0.5464711	6.79722656	-3.5693327	0.00194596	0.03885449	-1.4052066
ENSG00000164182.11_NDUFAF2	NDUFAF2	NADH:ubiquinone oxidoreductase complex assembly factor 2	HGNC:28086	5q12.1	0.53650737	3.57958197	3.54083946	0.00207834	0.04001704	-1.4086727
ENSG00000136305.11_CIDEB	CIDEB	cell death inducing DFFA like effector b	HGNC:1977	14q12	-1.5287006	-0.4424468	-3.5413662	0.00207582	0.04001704	-1.4110315
ENSG00000100139.13_MICALL1	MICALL1	MICAL like 1	HGNC:29804	22q13.1	-0.7942622	3.08029797	-3.5230643	0.00216538	0.04082574	-1.4114914
ENSG00000283632.2_EXOC3L2	EXOC3L2	exocyst complex component 3 like 2	HGNC:30162	19q13.32	-2.150248	-1.3720949	-3.7080152	0.00141157	0.03290341	-1.412929
ENSG00000144857.14_BOC	BOC	BOC cell adhesion associated, oncogene regulated	HGNC:17173	3q13.2	-0.7656062	2.61409446	-3.5062297	0.00225113	0.04188042	-1.4152304
ENSG00000010327.10_STAB1	STAB1	stabilin 1	HGNC:18628	3p21.1	-1.2017895	4.03412184	-3.5487071	0.00204093	0.03991387	-1.4162008
ENSG00000180155.20_LYNX1	LYNX1	Ly6/neurotoxin 1	HGNC:29604	8q24.3	-0.9404222	2.37648719	-3.4943119	0.00231384	0.04253546	-1.4162568
ENSG00000204681.11_GABBR1	GABBR1	gamma-aminobutyric acid type B receptor subunit 1	HGNC:4070	6p22.1	-0.6429992	3.36961436	-3.5338971	0.00211192	0.04023914	-1.4174945
ENSG00000136237.18_RAPGEF5	RAPGEF5	Rap guanine nucleotide exchange factor 5	HGNC:16862	7p15.3	-0.8061684	1.29408999	-3.4681228	0.00245776	0.04394212	-1.4196258
ENSG00000238142.1_BX284668.5	BX284668.5				1.49985883	0.44759448	3.49205605	0.0023259	0.04253546	-1.4216386
ENSG00000108107.14_RPL28	RPL28	ribosomal protein L28	HGNC:10330	19q13.42	0.53200916	7.64743387	3.55861345	0.00199476	0.03964419	-1.4243266
ENSG00000279833.1_AL031846.2	AL031846.2				-1.2618287	0.32519887	-3.4807949	0.00238705	0.04320296	-1.4257919
ENSG00000187210.14_GCNT1	GCNT1	glucosaminyl (N-acetyl) transferase 1	HGNC:4203	9q21.13	-1.4274485	-0.896929	-3.6131039	0.00175865	0.03667734	-1.428676
ENSG00000165792.17_METTL17	METTL17	methyltransferase like 17	HGNC:19280	14q11.2	0.32519223	5.03442135	3.55775167	0.00199873	0.03964419	-1.4298532
ENSG00000106610.15_STAG3L4	STAG3L4	stromal antigen 3-like 4 (pseudogene)	HGNC:33887	7q11.21	0.93156417	3.87460162	3.53639871	0.00209976	0.04005835	-1.4304994
ENSG00000143222.12_UFC1	UFC1	ubiquitin-fold modifier conjugating enzyme 1	HGNC:26941	1q23.3	0.39974496	4.62079936	3.55214933	0.00202477	0.03973999	-1.4336997
ENSG00000180209.11_MYLPF	MYLPF	myosin light chain, phosphorylatable, fast skeletal muscle	HGNC:29824	16p11.2	0.8641094	12.0565891	3.52269882	0.00216721	0.04082574	-1.4345278
ENSG00000070785.16_EIF2B3	EIF2B3	eukaryotic translation initiation factor 2B subunit gamma	HGNC:3259	1p34.1	0.48435761	5.33370735	3.55676328	0.0020033	0.03967625	-1.4349121
ENSG00000146066.3_HIGD2A	HIGD2A	HIG1 hypoxia inducible domain family member 2A	HGNC:28311	5q35.2	0.47143999	6.79577097	3.55625791	0.00200564	0.03967625	-1.4351606
ENSG00000174705.13_SH3PXD2B	SH3PXD2B	SH3 and PX domains 2B	HGNC:29242	5q35.1	-1.3847211	1.63633344	-3.4642639	0.0024797	0.04396425	-1.4359777
ENSG00000160360.13_GPSM1	GPSM1	G protein signaling modulator 1	HGNC:17858	9q34.3	-1.0533184	0.81223815	-3.4599489	0.00250445	0.0441296	-1.4366408
ENSG00000134759.14_ELP2	ELP2	elongator acetyltransferase complex subunit 2	HGNC:18248	18q12.2	0.4803224	5.3009378	3.55402831	0.002016	0.03973999	-1.440693
ENSG00000179348.11_GATA2	GATA2	GATA binding protein 2	HGNC:4171	3q21.3	-0.8727997	1.7952969	-3.4665811	0.0024665	0.04395542	-1.4448122
ENSG00000110200.8_ANAPC15	ANAPC15	anaphase promoting complex subunit 15	HGNC:24531	11q13.4	0.56798394	4.40200883	3.54291451	0.00206841	0.04001704	-1.4452893
ENSG00000204469.12_PRRC2A	PRRC2A	proline rich coiled-coil 2A	HGNC:13918	6p21.33	-0.5313642	6.76496538	-3.5512097	0.00202916	0.03977427	-1.4455456
ENSG00000271895.2_AL109811.2	AL109811.2				0.63906326	2.60803074	3.48399379	0.00236952	0.04301572	-1.4476482
ENSG00000078668.14_VDAC3	VDAC3	voltage dependent anion channel 3	HGNC:12674	8p11.21	0.47216864	8.3944382	3.54269497	0.00206946	0.04001704	-1.4483087
ENSG00000107731.12_UNC5B	UNC5B	unc-5 netrin receptor B	HGNC:12568	10q22.1	-1.0864769	1.25393691	-3.4525921	0.00254723	0.04462432	-1.4485176
ENSG00000157985.19_AGAP1	AGAP1	ArfGAP with GTPase domain, ankyrin repeat and PH domain 1	HGNC:16922	2q37.2	-0.4080277	4.18010052	-3.5394542	0.002085	0.04001704	-1.4493082
ENSG00000151617.17_EDNRA	EDNRA	endothelin receptor type A	HGNC:3179	4q31.22-q31.	-0.9066237	1.24778445	-3.451547	0.00255336	0.04462432	-1.4512524
ENSG00000141378.14_PTRH2	PTRH2	peptidyl-tRNA hydrolase 2	HGNC:24265	17q23.1	0.55934217	2.73618523	3.49006247	0.00233661	0.04262472	-1.4531774
ENSG00000168090.10_COPS6	COPS6	COP9 signalosome subunit 6	HGNC:21749	7q22.1	0.39807196	6.21998025	3.54799773	0.00204427	0.03991387	-1.4548347
ENSG00000126698.11_DNAJC8	DNAJC8	DnaJ heat shock protein family (Hsp40) member C8	HGNC:15470	1p35.3	0.43388366	5.12815857	3.5451921	0.00205756	0.03997699	-1.4584316
ENSG00000168067.12_MAP4K2	MAP4K2	mitogen-activated protein kinase kinase kinase kinase 2	HGNC:6864	11q13.1	-0.7335926	2.07410649	-3.466857	0.00246493	0.04395542	-1.4600107
ENSG00000140694.17_PARN	PARN	poly(A)-specific ribonuclease	HGNC:8609	16p13.12	0.4295731	3.36278438	3.51005593	0.00223135	0.04166756	-1.4603375
ENSG00000182372.9_CLN8	CLN8	CLN8 transmembrane ER and ERGIC protein	HGNC:2079	8p23.3	-0.5297774	3.62916034	-3.5201114	0.00218019	0.04091598	-1.4620739
ENSG00000083814.13_ZNF671	ZNF671	zinc finger protein 671	HGNC:26279	19q13.43	0.57151636	3.19187035	3.50170487	0.00227474	0.04221492	-1.4633274
ENSG00000087884.14_AAMDC	AAMDC	adipogenesis associated Mth938 domain containing	HGNC:30205	11q14.1	0.49511615	6.57999282	3.5401252	0.00208177	0.04001704	-1.4710566
ENSG00000277791.5_PSMB3	PSMB3	proteasome 20S subunit beta 3	HGNC:9540	17q12	0.5135996	5.42274131	3.53989001	0.0020829	0.04001704	-1.4718299

ENSG00000128791.12_TWSG1	TWSG1	twisted gastrulation BMP signaling modulator 1	HGNC:12429	18p11.22	-0.4596603	2.19770727	-3.4657686	0.00247112	0.04395542	-1.472208
ENSG00000178980.15_SELENOW	SELENOW	selenoprotein W	HGNC:10752	19q13.33	0.50595067	8.86241287	3.52764437	0.00214262	0.04058444	-1.4727115
ENSG00000126768.12_TIMM17B	TIMM17B	translocase of inner mitochondrial membrane 17B	HGNC:17310	Xp11.23	0.44582249	5.32222803	3.53917131	0.00208636	0.04001704	-1.4727614
ENSG00000173402.11_DAG1	DAG1	dystroglycan 1	HGNC:2666	3p21.31	-0.4222179	7.07488713	-3.537725	0.00209334	0.04003776	-1.4734017
ENSG00000072864.15_NDE1	NDE1	nudE neurodevelopment protein 1	HGNC:17619	16p13.11	-0.684627	1.78880413	-3.445352	0.00259003	0.04485036	-1.4818099
ENSG00000184584.13_TMEM173	TMEM173	stimulator of interferon response cGAMP interactor 1	HGNC:27962	5q31.2	-0.77035	2.99577674	-3.4888247	0.00234329	0.04269456	-1.4843604
ENSG00000178726.6_THBD	THBD	thrombomodulin	HGNC:11784	20p11.21	-0.7792397	3.24268887	-3.4957671	0.00230609	0.04253546	-1.4850775
ENSG00000115705.21_TPO	TPO	thyroid peroxidase	HGNC:12015	2p25.3	-1.1237871	1.42498309	-3.4344797	0.00265562	0.04556185	-1.4857821
ENSG00000004866.20_ST7	ST7	suppression of tumorigenicity 7	HGNC:11351	7q31.2	0.46613068	3.18976397	3.49262036	0.00232288	0.04253546	-1.48842
ENSG00000214050.8_FBXO16	FBXO16	F-box protein 16	HGNC:13618	8p21.1	2.08637818	-1.3090069	3.72927546	0.00134365	0.0318635	-1.4902388
ENSG00000156467.9_UQCRB	UQCRB	ubiquinol-cytochrome c reductase binding protein	HGNC:12582	8q22.1	0.59319072	8.62639791	3.52107131	0.00217536	0.04090773	-1.4909335
ENSG00000013288.8_MAN2B2	MAN2B2	mannosidase alpha class 2B member 2	HGNC:29623	4p16.1	-0.9019641	2.47851142	-3.4643969	0.00247894	0.04396425	-1.4946481
ENSG00000183621.15_ZNF438	ZNF438	zinc finger protein 438	HGNC:21029	10p11.23	0.40457624	3.3858143	3.4936568	0.00231734	0.04253546	-1.5003432
ENSG00000184635.15_ZNF93	ZNF93	zinc finger protein 93	HGNC:13169	19p12	1.34737797	0.02698673	3.47331577	0.00242854	0.04366416	-1.5135428
ENSG00000090013.10_BLVRB	BLVRB	biliverdin reductase B	HGNC:1063	19q13.2	0.40156661	5.93148847	3.5207417	0.00217702	0.04090773	-1.5145593
ENSG00000119681.12_LTBP2	LTBP2	latent transforming growth factor beta binding protein 2	HGNC:6715	14q24.3	-0.9298737	4.62805611	-3.51493	0.0022064	0.04130461	-1.5157035
ENSG00000136819.15_C9orf78	C9orf78	chromosome 9 open reading frame 78	HGNC:24932	9q34.11	0.36922457	5.00737865	3.51762423	0.00219273	0.0411	-1.517181
ENSG00000281490.1_CICP14	CICP14	capicua transcriptional repressor pseudogene 14	HGNC:38542	7q32.1	-0.6674747	3.21855535	-3.4747916	0.0024203	0.04356822	-1.5172934
ENSG00000100644.17_HIF1A	HIF1A	hypoxia inducible factor 1 subunit alpha	HGNC:4910	14q23.2	-0.5564098	2.64485315	-3.4590346	0.00250973	0.04417068	-1.518977
ENSG00000229644.6_NAMPTP1	NAMPTP1	nicotinamide phosphoribosyltransferase pseudogene 1	HGNC:17633	10p11.21	2.05583513	-0.450774	3.52690413	0.00214628	0.04058444	-1.5191534
ENSG00000241990.5_PRR34-AS1	PRR34-AS1	PRR34 antisense RNA 1	HGNC:50499	22q13.31	0.66586683	3.8163152	3.49480767	0.0023112	0.04253546	-1.5212582
ENSG00000183943.5_PRKX	PRKX	protein kinase X-linked	HGNC:9441	Xp22.33	-0.7147969	0.85832261	-3.4141348	0.00278279	0.04685775	-1.522122
ENSG00000140406.3_TLNRD1	TLNRD1	talin rod domain containing 1	HGNC:13519	15q25.1	-1.1522864	1.64820865	-3.4200711	0.00274508	0.04665318	-1.5246593
ENSG00000171530.14_TBCA	TBCA	tubulin folding cofactor A	HGNC:11579	5q14.1	0.59857331	4.56460728	3.50914862	0.00223602	0.04170292	-1.524806
ENSG00000278974.1_AC093909.6	AC093909.6				0.74592889	1.47770229	3.41464098	0.00277955	0.04685775	-1.5262295
ENSG00000163634.12_THOC7	THOC7	THO complex 7	HGNC:29874	3p14.1	0.52134209	4.7970419	3.5109573	0.00222671	0.04163288	-1.5274993
ENSG00000100060.17_MFNG	MFNG	MFNG O-fucosylpeptide 3-beta-N-acetylglucosaminyltransfera	HGNC:7038	22q13.1	-0.6695767	1.85089134	-3.4254808	0.00271115	0.04619737	-1.5307766
ENSG00000268713.1_AC005261.3	AC005261.3				1.16527869	-0.3021762	3.47926488	0.00239548	0.04322532	-1.5309886
ENSG00000054598.8_FOXC1	FOXC1	forkhead box C1	HGNC:3800	6p25.3	-0.933152	0.75323734	-3.4098793	0.00281013	0.04685775	-1.5330025
ENSG00000233297.4_RASA4DP	RASA4DP	RAS p21 protein activator 4CD, pseudogene	HGNC:44226	7q22.1	3.65375689	1.94290564	3.41335632	0.00278777	0.04685775	-1.5346006
ENSG00000078018.19_MAP2	MAP2	microtubule associated protein 2	HGNC:6839	2q34	-0.8765788	0.27929105	-3.4157661	0.00277238	0.04685775	-1.537047
ENSG00000081803.16_CADPS2	CADPS2	calcium dependent secretion activator 2	HGNC:16018	7q31.32	-0.9347787	0.42331661	-3.4112311	0.00280142	0.04685775	-1.5444692
ENSG00000168546.11_GFRA2	GFRA2	GDNF family receptor alpha 2	HGNC:4244	8p21.3	-2.1209706	-0.8482229	-3.5289738	0.00213605	0.04056584	-1.5461122
ENSG00000139926.15_FRMD6	FRMD6	FERM domain containing 6	HGNC:19839	14q22.1	-1.1085881	2.48000949	-3.4363726	0.00264409	0.04546796	-1.5496637
ENSG00000128578.10_STRIP2	STRIP2	striatin interacting protein 2	HGNC:22209	7q32.1	0.5831686	5.34662681	3.50260134	0.00227004	0.04217996	-1.5526818
ENSG00000136010.14_ALDH1L2	ALDH1L2	aldehyde dehydrogenase 1 family member L2	HGNC:26777	12q23.3	-0.7375819	0.39325365	-3.4026643	0.0028571	0.04692616	-1.5553848
ENSG00000135940.6_COX5B	COX5B	cytochrome c oxidase subunit 5B	HGNC:2269	2q11.2	0.57186688	8.61673123	3.49049598	0.00233428	0.04262472	-1.5575637
ENSG00000198046.12_ZNF667	ZNF667	zinc finger protein 667	HGNC:28854	19q13.43	0.78933548	0.58581963	3.40273452	0.00285664	0.04692616	-1.5586784
ENSG00000166171.13_DPCD	DPCD	deleted in primary ciliary dyskinesia homolog (mouse)	HGNC:24542	10q24.32	0.58233101	1.89937757	3.40939917	0.00281324	0.04685775	-1.5596053
ENSG00000137970.7_RPL7P9	RPL7P9	ribosomal protein L7 pseudogene 9	HGNC:37028	1p21.3	0.77880509	0.75385315	3.39742318	0.0028917	0.04714227	-1.5598586
ENSG00000094916.16_CBX5	CBX5	chromobox 5	HGNC:1555	12q13.13	0.40581366	4.62567356	3.49396545	0.00231569	0.04253546	-1.5601301
ENSG00000100764.14_PSMC1	PSMC1	proteasome 26S subunit, ATPase 1	HGNC:9547	14q32.11	0.49649124	7.01730287	3.49777566	0.00229544	0.04249391	-1.561127
ENSG00000074071.14_MRPS34	MRPS34	mitochondrial ribosomal protein S34	HGNC:16618	16p13.3	0.49298313	5.59349605	3.49872662	0.00229041	0.04245326	-1.562047
ENSG00000151789.12_ZNF385D	ZNF385D	zinc finger protein 385D	HGNC:26191	3p24.3	-0.9760216	0.19777233	-3.4115894	0.00279911	0.04685775	-1.5624014
ENSG00000155304.6_HSPA13	HSPA13	heat shock protein family A (Hsp70) member 13	HGNC:11375	21q11.2	-0.6447556	1.30760529	-3.396379	0.00289864	0.04714227	-1.5627988
ENSG00000127863.15_TNFRSF19	TNFRSF19	TNF receptor superfamily member 19	HGNC:11915	13q12.12	-1.4126836	0.63978384	-3.3966097	0.00289711	0.04714227	-1.5636467
ENSG00000167088.11_SNRPD1	SNRPD1	small nuclear ribonucleoprotein D1 polypeptide	HGNC:11158	18q11.2	0.43735471	4.06002992	3.4833483	0.00237305	0.04302766	-1.5636495
ENSG00000144366.16_GULP1	GULP1	GULP PTB domain containing engulfment adaptor 1	HGNC:18649	2q32.1-q32.2	-0.8025148	1.41626403	-3.3987013	0.00288323	0.04714227	-1.5665285
ENSG00000196639.6_HRH1	HRH1	histamine receptor H1	HGNC:5182	3p25.3	-1.0995685	0.07685932	-3.4031039	0.00285422	0.04692616	-1.5669736
ENSG00000042753.11_AP2S1	AP2S1	adaptor related protein complex 2 subunit sigma 1	HGNC:565	19q13.32	0.44618319	4.88235032	3.49137574	0.00231991	0.04253546	-1.566989
ENSG00000137509.11_PRCP	PRCP	prolylcarboxypeptidase	HGNC:9344	11q14	-0.7356431	3.22226871	-3.4577017	0.00251744	0.04425448	-1.5674863
ENSG00000122707.12_RECK	RECK	reversion inducing cysteine rich protein with kazal motifs	HGNC:11345	9p13.3	-0.9634089	1.77053317	-3.4042221	0.0028469	0.04692616	-1.5711163
ENSG00000135624.16_CCT7	CCT7	chaperonin containing TCP1 subunit 7	HGNC:1622	2p13.2	0.41593295	7.324844	3.49202887	0.00232605	0.04253546	-1.5714701
ENSG00000164258.12_NDUFS4	NDUFS4	NADH:ubiquinone oxidoreductase subunit S4	HGNC:7711	5q11.2	0.57054916	6.19292209	3.49294465	0.00232114	0.04253546	-1.5745358
ENSG00000164930.12_FZD6	FZD6	frizzled class receptor 6	HGNC:4044	8q22.3	-0.8794611	0.67069104	-3.3851614	0.00297427	0.04775065	-1.5817585
ENSG00000127184.13_COX7C	COX7C	cytochrome c oxidase subunit 7C	HGNC:2292	5q14.3	0.63577325	8.71409977	3.4782756	0.00240095	0.04327188	-1.5821516
ENSG00000102034.16_ELF4	ELF4	E74 like ETS transcription factor 4	HGNC:3319	Xq26.1	-1.2212524	0.91261012	-3.3830164	0.00298895	0.04788379	-1.5833383
ENSG00000065518.8_NDUFB4	NDUFB4	NADH:ubiquinone oxidoreductase subunit B4	HGNC:7699	3q13.33	0.47684005	7.4457413	3.48448538	0.00236684	0.04301572	-1.5867396
ENSG00000162430.17_SELENON	SELENON	selenoprotein N	HGNC:15999	1p36.11	-0.7028536	4.68094261	-3.4805165	0.00238858	0.04320296	-1.5903625
ENSG00000130598.16_TNNI2	TNNI2	troponin I2, fast skeletal type	HGNC:11946	11p15.5	0.88596496	11.6986805	3.45182895	0.00255171	0.04462432	-1.590937
ENSG00000215769.8_ARHGAP27P1-BF	ARHGAP27P1-BPTFP1-KPNA2	ARHGAP27P1-BPTFP1-KPNA2P3 readthrough, transcribed pse	HGNC:52873	17q24.1	-0.6074297	1.79511897	-3.3909281	0.00293515	0.04739373	-1.5922897
ENSG00000198373.12_WWP2	WWP2	WW domain containing E3 ubiquitin protein ligase 2	HGNC:16804	16q22.1	-0.4933536	3.88396166	-3.4641387	0.00248041	0.04396425	-1.5938701
ENSG00000158966.15_CACHD1	CACHD1	cache domain containing 1	HGNC:29314	1p31.3	-0.6006169	1.37694891	-3.3812216	0.00300128	0.04797891	-1.5953195
ENSG00000127947.16_PTPN12	PTPN12	protein tyrosine phosphatase non-receptor type 12	HGNC:9645	7q11.23	-0.513049	3.78017921	-3.4607051	0.0025001	0.04410466	-1.600277
ENSG00000054179.12_ENTPD2	ENTPD2	ectonucleoside triphosphate diphosphohydrolase 2	HGNC:3364	9q34.3	-1.4481154	-0.6716554	-3.484319	0.00236775	0.04301572	-1.600992
ENSG00000126746.17_ZNF384	ZNF384	zinc finger protein 384	HGNC:11955	12p13.31	-0.5726003	3.4901916	-3.4500469	0.00256219	0.04468334	-1.601653
ENSG00000184752.13_NDUFA12	NDUFA12	NADH:ubiquinone oxidoreductase subunit A12	HGNC:23987	12q22	0.48475568	6.3981886	3.48001211	0.00239136	0.04320296	-1.6020955
ENSG00000231500.7_RPS18	RPS18	ribosomal protein S18	HGNC:10401	6p21.32	0.56263759	8.91580979	3.46603528	0.0024696	0.04395542	-1.6042965
ENSG00000153933.10_DGKE	DGKE	diacylglycerol kinase epsilon	HGNC:2852	17q22	-0.8255502	0.7497022	-3.37289	0.00305921	0.04854268	-1.6052025
ENSG00000144891.17_AGTR1	AGTR1	angiotensin II receptor type 1	HGNC:336	3q24	-0.8107336	2.10453213	-3.3980042	0.00288785	0.04714227	-1.606197
ENSG00000204922.5_UQCC3	UQCC3	ubiquinol-cytochrome c reductase complex assembly factor 3	HGNC:34399	11q12.3	0.58780827	3.87647767	3.45630236	0.00252557	0.04434528	-1.6074177
ENSG00000286191.1_AL158195.1	AL158195.1				1.87887795	-0.2524254	3.46847153	0.00245578	0.04394212	-1.6086489
ENSG00000002834.18_LASP1	LASP1	LIM and SH3 protein 1	HGNC:6513	17q12	-0.8046778	4.36673506	-3.4686227	0.00245493	0.04394212	-1.6087492
ENSG00000177363.5_LRRN4CL	LRRN4CL	LRRN4 C-terminal like	HGNC:33724	11q12.3	-1.1865924	1.96313447	-3.3893638	0.00294571	0.04739373	-1.6088984
ENSG00000114200.10_BCHE	BCHE	butyrylcholinesterase	HGNC:983	3q26.1	-1.0433205	0.29006334	-3.3816473	0.00299835	0.04797891	-1.6109128
ENSG00000090539.15_CHRD	CHRD	chordin	HGNC:1949	3q27.1	-0.6581293	2.34873643	-3.3995957	0.00287731	0.04714227	-1.6146454
ENSG00000103047.8_TANGO6	TANGO6	transport and golgi organization 6 homolog	HGNC:25749	16q22.1	0.45721794	3.01140386	3.42668199	0.00270367	0.04612238	-1.6150975
ENSG00000178202.13_KDELC2	KDELC2	protein O-glucosyltransferase 3	HGNC:28496	11q22.3	-0.599863	2.74899678	-3.4172506	0.00276293	0.0467609	-1.6165592
ENSG00000151176.8_PLBD2	PLBD2	phospholipase B domain containing 2	HGNC:27283	12q24.13	-0.5052049	4.34723523	-3.4634258	0.00248449	0.04398417	-1.6178472
ENSG00000170296.10_GABARAP	GABARAP	GABA type A receptor-associated protein	HGNC:4067	17p13.1	0.44191444	7.38143188	3.46964875	0.00244914	0.04394212	-1.6197152
ENSG00000164741.15_DLC1	DLC1	DLC1 Rho GTPase activating protein	HGNC:2897	8p22	-0.5745956	2.73082943	-3.4181914	0.00275696	0.04673783	-1.6223032
ENSG00000162961.14_DPY30	DPY30	dpy-30 histone methyltransferase complex regulatory subunit	HGNC:24590	2p22.3	0.51236646	3.48506803	3.43966252	0.00262415	0.04517697	-1.6231736
ENSG00000174547.13_MRPL11	MRPL11	mitochondrial ribosomal protein L11	HGNC:14042	11q13.2	0.46527409	5.1063075	3.46796537	0.00245865	0.04394212	-1.6254725
ENSG00000118495.19_PLAGL1	PLAGL1	PLAG1 like zinc finger 1	HGNC:9046	6q24.2	-1.0802622	2.24011442	-3.3915533	0.00293094	0.04739373	-1.6256239
ENSG00000164309.15_CMYA5	CMYA5	cardiomyopathy associated 5	HGNC:14305	5q14.1	0.60103712	11.6524389	3.43057517	0.00267958	0.04586016	-1.6357212
ENSG00000132563.16_REEP2	REEP2	receptor accessory protein 2	HGNC:17975	5q31.2	-0.7428494	2.46297225	-3.3930458	0.00292092	0.04738259	-1.6399299
ENSG00000130638.17_ATXN10	ATXN10	ataxin 10	HGNC:10549	22q13.31	0.36893082	5.67481552	3.46291644	0.0024874	0.04398417	-1.6400295
ENSG00000100138.15_SNU13	SNU13	small nuclear ribonucleoprotein 13	HGNC:7819	22q13.2	0.42554428	6.29982656	3.46070816	0.00250008	0.04410466	-1.6442289
ENSG00000173163.10_COMMD1	COMMD1	copper metabolism domain containing 1	HGNC:23024	2p15	0.55191946	3.82911445	3.43568495	0.00264827	0.04548777	-1.6474616
ENSG00000261641.2_AL031600.3	AL031600.3				3.23108665	-1.3949125	3.60419116	0.00179529	0.03708041	-1.6480677
ENSG00000102760.13_RGCC	RGCC	regulator of cell cycle	HGNC:20369	13q14.11	-0.5693019	2.9948853	-3.414562	0.00278006	0.04685775	-1.6483253
ENSG00000275993.2_SIK1B	SIK1B	salt inducible kinase 1B (putative)	HGNC:52389	21p12	-2.0887466	-0.0839136	-3.3896419	0.00294383	0.04739373	-1.651216
ENSG00000165675.16_ENOX2	ENOX2	ecto-NOX disulfide-thiol exchanger 2	HGNC:2259	Xq26.1	0.4730237	2.65166755	3.38954195	0.00294451	0.04739373	-1.6551364
ENSG00000134851.13_TMEM165	TMEM165	transmembrane protein 165	HGNC:30760	4q12	-0.6609134	2.9110756	-3.4038779	0.00284915	0.04692616	-1.6554124
ENSG00000174951.11_FUT1	FUT1	fucosyltransferase 1 (H blood group)	HGNC:4012	19q13.33	-1.1399892	0.24579763	-3.3537657	0.00319635	0.04982187	-1.6602941
ENSG00000158042.8_MRPL17	MRPL17	mitochondrial ribosomal protein L17	HGNC:14053	11p15.4	0.44616087	3.89913136	3.43334853	0.00266254	0.04562833	-1.6608512
ENSG00000184076.13_UQCR10	UQCR10	ubiquinol-cytochrome c reductase, complex III subunit X	HGNC:30863	22q12.2	0.60196789	7.27264541	3.4499611	0.0025627	0.04468334	-1.6632285
ENSG00000143499.14_SMYD2	SMYD2	SET and MYND domain containing 2	HGNC:20982	1q32.3	0.54051818	5.67997486	3.45179005	0.00255193	0.04462432	-1.6640242
ENSG00000167283.8_ATP5MG	ATP5MG	ATP synthase membrane subunit g	HGNC:14247	11q23.3	0.52669966	6.90873155	3.44848975	0.00257139	0.04478282	-1.668443
ENSG00000182218.9_HHIPL1	HHIPL1	HHIP like 1	HGNC:19710	14q32.2	-1.4288899	-0.7230561	-3.4472327	0.00257884	0.04485036	-1.6711556
ENSG00000088808.17_PPP1R13B	PPP1R13B	protein phosphatase 1 regulatory subunit 13B	HGNC:14950	14q32.33	-0.4193133	3.24164425	-3.4090447	0.00281553	0.04685775	-1.6744155

ENSG00000164919.11_COX6C	COX6C	cytochrome c oxidase subunit 6C	HGNC:2285	8q22.2	0.58359292	7.59381356	3.44296912	0.00260426	0.04498956	-1.6755038
ENSG00000113141.18_IK	IK	IK cytokine	HGNC:5958	5q31.3	0.4387013	6.05719965	3.44643329	0.00258359	0.04485036	-1.6755416
ENSG00000100348.10_TXN2	TXN2	thioredoxin 2	HGNC:17772	22q12.3	0.42606406	6.28681855	3.44577791	0.00258749	0.04485036	-1.6765878
ENSG00000176171.11_BNIP3	BNIP3	BCL2 interacting protein 3	HGNC:1084	10q26.3	0.54590794	6.59921674	3.4453185	0.00259022	0.04485036	-1.6766421
ENSG00000175768.13_TOMM5	TOMM5	translocase of outer mitochondrial membrane 5	HGNC:31369	9p13.2	0.52815952	4.88469356	3.44166954	0.00261206	0.04507235	-1.6786753
ENSG00000145919.10_BOD1	BOD1	biorientation of chromosomes in cell division 1	HGNC:25114	5q35.2	0.42949159	5.24050909	3.44325693	0.00260254	0.04498956	-1.6808865
ENSG00000183617.5_MRPL54	MRPL54	mitochondrial ribosomal protein L54	HGNC:16685	19p13.3	0.55767331	3.90563368	3.42215074	0.00273199	0.04649958	-1.6835455
ENSG00000118855.19_MFSD1	MFSD1	major facilitator superfamily domain containing 1	HGNC:25874	3q25.32	-0.5490838	2.89112329	-3.3927498	0.0029229	0.04738259	-1.6838749
ENSG00000185721.12_DRG1	DRG1	developmentally regulated GTP binding protein 1	HGNC:3029	22q12.2	0.53228015	5.7949442	3.44016337	0.00262113	0.04517681	-1.6892924
ENSG00000125746.16_EML2	EML2	EMAP like 2	HGNC:18035	19q13.32	-0.8064347	2.07756124	-3.3513654	0.00321398	0.04990939	-1.6897718
ENSG00000148690.12_FRA10AC1	FRA10AC1	FRA10A associated CGG repeat 1	HGNC:1162	10q23.33	0.56469311	3.58221422	3.41061037	0.00280542	0.04685775	-1.6905025
ENSG00000133983.15_COX16	COX16	cytochrome c oxidase assembly factor COX16	HGNC:20213	14q24.2	0.52085232	4.54076282	3.42780403	0.00269671	0.04605593	-1.6997818
ENSG00000104774.13_MAN2B1	MAN2B1	mannosidase alpha class 2B member 1	HGNC:6826	19p13.13	-0.6801472	3.59048014	-3.405781	0.00283672	0.04692616	-1.702435
ENSG00000173226.17_IQCB1	IQCB1	IQ motif containing B1	HGNC:28949	3q13.33	0.44270926	2.29976041	3.35456778	0.00319048	0.04982187	-1.7066835
ENSG00000196498.13_NCOR2	NCOR2	nuclear receptor corepressor 2	HGNC:7673	12q24.31	-0.5916847	6.68467334	-3.4301521	0.00268219	0.04586016	-1.708463
ENSG00000110536.14_PTPMT1	PTPMT1	protein tyrosine phosphatase mitochondrial 1	HGNC:26965	11p11.2	0.45457571	4.026897	3.41375107	0.00278524	0.04685775	-1.710549
ENSG00000082515.18_MRPL22	MRPL22	mitochondrial ribosomal protein L22	HGNC:14480	5q33.2	0.42758078	3.91519537	3.40480592	0.00284308	0.04692616	-1.724172
ENSG00000151491.14_EPS8	EPS8	epidermal growth factor receptor pathway substrate 8	HGNC:3420	12p12.3	-0.4014774	3.558273	-3.3989178	0.00288179	0.04714227	-1.7246729
ENSG00000255310.2_AF131215.5	AF131215.5				-1.7376898	-1.8599012	-3.564561	0.00196754	0.03918091	-1.7252381
ENSG00000024862.17_CCDC28A	CCDC28A	coiled-coil domain containing 28A	HGNC:21098	6q24.1	0.45069847	2.56146994	3.35286548	0.00320295	0.04982187	-1.7263673
ENSG00000169554.20_ZEB2	ZEB2	zinc finger E-box binding homeobox 2	HGNC:14881	2q22.3	-0.4405409	3.95191866	-3.4052468	0.0028402	0.04692616	-1.7291599
ENSG00000101773.19_RBBP8	RBBP8	RB binding protein 8, endonuclease	HGNC:9891	18q11.2	-0.8447686	-0.4547888	-3.3511954	0.00321524	0.04990939	-1.7303017
ENSG00000188612.12_SUMO2	SUMO2	small ubiquitin like modifier 2	HGNC:11125	17q25	0.39757619	5.56517449	3.41972967	0.00274723	0.04665318	-1.7332741
ENSG00000180992.7_MRPL14	MRPL14	mitochondrial ribosomal protein L14	HGNC:14279	6p21.1	0.58287337	5.86785777	3.41795668	0.00275845	0.04673783	-1.7372679
ENSG00000236830.6_CBR3-AS1	CBR3-AS1	CBR3 antisense RNA 1	HGNC:43664	21q22.12	-0.9430005	-0.8263849	-3.4053911	0.00283926	0.04692616	-1.7377883
ENSG00000184988.8_TMEM106A	TMEM106A	transmembrane protein 106A	HGNC:28288	17q21.31	-1.3791771	-0.3264606	-3.3529264	0.00320251	0.04982187	-1.738128
ENSG00000204301.6_NOTCH4	NOTCH4	notch receptor 4	HGNC:7884	6p21.32	-0.7593938	4.41367828	-3.4055423	0.00283827	0.04692616	-1.7463567
ENSG00000108826.16_MRPL27	MRPL27	mitochondrial ribosomal protein L27	HGNC:14483	17q21.33	0.55704434	5.16594366	3.41216776	0.0027954	0.04685775	-1.7468258
ENSG00000214548.17_MEG3	MEG3	maternally expressed 3	HGNC:14575	14q32.2	-0.7891894	6.22528115	-3.4124944	0.0027933	0.04685775	-1.7479475
ENSG00000111752.11_PHC1	PHC1	polyhomeotic homolog 1	HGNC:3182	12p13.31	-0.4645431	3.54092188	-3.383473	0.00298581	0.04788379	-1.7529243
ENSG00000133116.8_KL	KL	klotho	HGNC:6344	13q13.1	-1.195512	-0.5893859	-3.3605436	0.00314707	0.04944745	-1.7586744
ENSG00000241837.7_ATP5PO	ATP5PO	ATP synthase peripheral stalk subunit OSCP	HGNC:850	21q22.11	0.53672837	7.87054089	3.40265058	0.00285719	0.04692616	-1.759229
ENSG00000142168.14_SOD1	SOD1	superoxide dismutase 1	HGNC:11179	21q22.11	0.48748831	6.88523257	3.40505429	0.00284146	0.04692616	-1.7625281
ENSG00000173210.19_ABLIM3	ABLIM3	actin binding LIM protein family member 3	HGNC:29132	5q32	-0.4940546	4.72949093	-3.3970195	0.00289438	0.04714227	-1.772878
ENSG00000173915.16_ATP5MD	ATP5MD	ATP synthase membrane subunit DAPIT	HGNC:30889	10q24.33	0.63130533	6.78889689	3.39863105	0.00288369	0.04714227	-1.7767096
ENSG00000131788.16_PIAS3	PIAS3	protein inhibitor of activated STAT 3	HGNC:16861	1q21.1	-0.5004761	3.45002668	-3.3662619	0.00310607	0.04901307	-1.7777227
ENSG00000112304.11_ACOT13	ACOT13	acyl-CoA thioesterase 13	HGNC:20999	6p22.3	0.61963282	4.30884873	3.38756198	0.00295792	0.04753916	-1.7791356
ENSG00000215021.8_PHB2	PHB2	prohibitin 2	HGNC:30306	12p13.31	0.36600419	7.1098169	3.39328399	0.00291932	0.04738259	-1.7866283
ENSG00000142733.16_MAP3K6	MAP3K6	mitogen-activated protein kinase kinase kinase 6	HGNC:6858	1p36.11	-0.7492176	3.18540625	-3.3545442	0.00319065	0.04982187	-1.7924354
ENSG00000106991.13_ENG	ENG	endoglin	HGNC:3349	9q34.11	-0.7926486	5.28460178	-3.3911501	0.00293366	0.04739373	-1.792494
ENSG00000106733.21_NMRK1	NMRK1	nicotinamide riboside kinase 1	HGNC:26057	9q21.13	0.56628801	4.01090376	3.37462581	0.00304705	0.04840097	-1.7938553
ENSG00000087302.9_RTRAF	RTRAF	RNA transcription, translation and transport factor	HGNC:23169	14q22.1	0.45824092	5.68496567	3.39048041	0.00293817	0.04739373	-1.7965775
ENSG00000170430.10_MGMT	MGMT	O-6-methylguanine-DNA methyltransferase	HGNC:7059	10q26.3	0.57236507	3.96448429	3.37016041	0.00307842	0.048693	-1.7967164
ENSG00000171953.16_ATPAF2	ATPAF2	ATP synthase mitochondrial F1 complex assembly factor 2	HGNC:18802	17p11.2	0.447051	3.79535164	3.360248	0.0031492	0.04944745	-1.8126108
ENSG00000189043.10_NDUFA4	NDUFA4	NDUFA4 mitochondrial complex associated	HGNC:7687	7p21.3	0.56016447	8.66742322	3.3717615	0.00306714	0.04861706	-1.812629
ENSG00000217930.8_PAM16	PAM16	presequence translocase associated motor 16	HGNC:29679	16p13.3	0.47203724	4.46364021	3.37123695	0.00307083	0.0486242	-1.8193917
ENSG00000100234.11_TIMP3	TIMP3	TIMP metallopeptidase inhibitor 3	HGNC:11822	22q12.3	-0.7319181	6.75583895	-3.3777606	0.00302521	0.04825859	-1.8198284
ENSG00000150459.12_SAP18	SAP18	Sin3A associated protein 18	HGNC:10530	13q12.11	0.39663877	6.88795258	3.37704796	0.00303016	0.04828619	-1.8227924
ENSG00000131143.8_COX4I1	COX4I1	cytochrome c oxidase subunit 4I1	HGNC:2265	16q24.1	0.52888892	8.56061261	3.36728834	0.00309877	0.04896318	-1.8239079
ENSG00000174886.13_NDUFA11	NDUFA11	NADH:ubiquinone oxidoreductase subunit A11	HGNC:20371	19p13.3	0.46555229	6.36365559	3.375291	0.0030424	0.04839048	-1.8286438
ENSG00000132329.11_RAMP1	RAMP1	receptor activity modifying protein 1	HGNC:9843	2q37.3	0.51445155	5.83140433	3.37518226	0.00304316	0.04839048	-1.8295829
ENSG00000065268.10_WDR18	WDR18	WD repeat domain 18	HGNC:17956	19p13.3	0.53257234	4.1586265	3.35724919	0.00317093	0.04968452	-1.8353183
ENSG00000178035.12_IMPDH2	IMPDH2	inosine monophosphate dehydrogenase 2	HGNC:6053	3p21.31	0.53191781	6.45737513	3.36290159	0.0031301	0.04925064	-1.8549857
ENSG00000198753.12_PLXNB3	PLXNB3	plexin B3	HGNC:9105	Xq28	-1.4950417	-1.1459072	-3.3659263	0.00310846	0.04901307	-1.8676439
ENSG00000184544.11_DHRS7C	DHRS7C	dehydrogenase/reductase 7C	HGNC:32423	17p13.1	0.52111468	6.13677364	3.35635244	0.00317746	0.0497348	-1.8698045
ENSG00000170681.6_CAVIN4	CAVIN4	caveolae associated protein 4	HGNC:33742	9q31.1	0.56895935	7.35733357	3.35344575	0.0031987	0.04982187	-1.8703473
ENSG00000171291.8_ZNF439	ZNF439	zinc finger protein 439	HGNC:20873	19p13.2	-1.3557665	-1.2267101	-3.3793147	0.00301444	0.048138	-1.8878963
ENSG00000146151.13_HMGCLL1	HMGCLL1	3-hydroxymethyl-3-methylglutaryl-CoA lyase like 1	HGNC:21359	6p12.1	-1.4192916	-1.3245152	-3.3503113	0.00322176	0.0499589	-1.9061925
ENSG00000138166.6_DUSP5	DUSP5	dual specificity phosphatase 5	HGNC:3071	10q25.2	-1.7079611	-1.2767473	-3.357648	0.00316803	0.04968452	-1.931726
ENSG00000205266.10_KRT17P5	KRT17P5	keratin 17 pseudogene 5	HGNC:50723	17p11.2	2.34892788	-1.5760483	3.40909793	0.00281518	0.04685775	-1.9488713
ENSG00000196581.10_AJAP1	AJAP1	adherens junctions associated protein 1	HGNC:30801	1p36.32	-2.0447234	-1.4733323	-3.3638185	0.00312353	0.04919882	-1.9886567

Supplementary Table 3. Top protein coding differentially expressed genes in CKD.

Gene Symbol	Approved Gene Name	log2(Fold Change)	Adjusted P-value
ZBED6	zinc finger BED-type containing 6	6.330096781	0.001324311
TNFRSF10A	TNF receptor superfamily member 10a	-2.002062713	0.010853085
FPR3	formyl peptide receptor 3	-2.152375106	0.018597767
CNTN4	contactin 4	-2.099431388	0.018116692
ALX4	ALX homeobox 4	-2.248772336	0.020512411
EXOC3L2	exocyst complex component 3 like 2	-2.150247955	0.032903406
FBXO16	F-box protein 16	2.086378177	0.031863499
GFRA2	GDNF family receptor alpha 2	-2.120970567	0.04056584
SIK1B	salt inducible kinase 1B (putative)	-2.0887466	0.047393725
AJAP1	adherens junctions associated protein 1	-2.044723353	0.049198819

Supplementary Table 4. Pathway analysis.

p-value	q-value	pathway	source	external_id	members_input_overlap	members_input_overlap_geneids	size	effective_size
2.23E-69	3.64E-67	Eukaryotic Translation Elongation	Reactome	R-HSA-156842	RPS13; RPS12; RPS11; RPS10; RPS17; RPS16; RPL27A; RPL9; RPL6; RP	4736; 6124; 6203; 6137; 6227; 6161	106	105
8.93E-69	5.28E-67	Formation of a pool of free 40S subunits	Reactome	R-HSA-72689	RPS13; RPS12; RPS11; RPS10; RPS17; RPS16; RPL27A; RPL9; RPL6; RP	4736; 6124; 6203; 6137; 6227; 6161	115	113
9.82E-69	5.28E-67	Eukaryotic Translation Termination	Reactome	R-HSA-72764	TRMT112; RPS12; RPS11; RPS10; RPS17; RPS16; RPL27A; RPL9; RPL6;	4736; 6124; 6203; 6137; 6227; 6161	104	103
1.30E-68	5.28E-67	Peptide chain elongation	Reactome	R-HSA-156902	RPS13; RPS12; RPS11; RPS10; RPS17; RPS16; RPL27A; RPL9; RPL6; RP	4736; 6124; 6203; 6137; 6227; 6161	101	100
4.38E-67	1.43E-65	Selenocysteine synthesis	Reactome	R-HSA-2408557	RPS13; RPS12; RPS11; RPS10; RPS17; RPS16; RPL27A; RPL9; RPL6; RP	4736; 6124; 6203; 6137; 6227; 6161	104	103
4.09E-66	1.11E-64	Nonsense Mediated Decay (NMD) independent of the Exon Junction Complex (EJC)	Reactome	R-HSA-975956	RPS13; RPS12; RPS11; RPS10; RPS17; RPS16; RPL27A; RPL9; RPL6; RP	4736; 6124; 6203; 6137; 6227; 6161	106	105
5.01E-66	1.17E-64	L13a-mediated translational silencing of Ceruloplasmin expression	Reactome	R-HSA-156827	RPS13; RPS12; RPS11; RPS10; RPS17; RPS16; RPL27A; RPL9; RPL6; RP	4736; 6124; 6203; 6137; 6227; 6161	125	123
1.26E-65	2.56E-64	GTP hydrolysis and joining of the 60S ribosomal subunit	Reactome	R-HSA-72706	RPS13; RPS12; RPS11; RPS10; RPS17; RPS16; RPL27A; RPL9; RPL6; RP	4736; 6124; 6203; 6137; 6227; 6161	126	124
2.98E-65	5.39E-64	Translation	Reactome	R-HSA-72766	TRMT112; RPL21P16; RPL22; EIF3H; EIF3I; EIF3K; EIF3L; MRPL54; MR	4736; 6124; 6137; 25873; 6227; 904	310	307
1.56E-64	2.54E-63	SRP-dependent cotranslational protein targeting to membrane	Reactome	R-HSA-1799339	RPS13; RPS12; RPS11; RPS10; RPS17; RPS16; RPL27A; RPL9; RPL6; RP	4736; 6124; 6203; 6137; 6227; 6161	124	123
6.36E-64	9.43E-63	Ribosome - Homo sapiens (human)	KEGG	path:hsa03010	RPS13; RPS12; RPS11; RPL37A; RPS17; RPS16; RPL23A; MRPS21; RPL	6144; 6146; 6147; 6152; 6154; 6156	153	153
5.56E-63	6.97E-62	Cap-dependent Translation Initiation	Reactome	R-HSA-72737	RPS13; RPS12; RPS11; RPS10; RPS17; RPS16; RPL27A; RPL9; RPL6; RP	4736; 6124; 6203; 6137; 6227; 6161	133	131
5.56E-63	6.97E-62	Eukaryotic Translation Initiation	Reactome	R-HSA-72613	RPS13; RPS12; RPS11; RPS10; RPS17; RPS16; RPL27A; RPL9; RPL6; RP	4736; 6124; 6203; 6137; 6227; 6161	133	131
5.92E-61	6.43E-60	Nonsense Mediated Decay (NMD) enhanced by the Exon Junction Complex (EJC)	Reactome	R-HSA-975957	RPS13; RPS12; RPS11; RPS10; RPS17; RPS16; RPL27A; RPL9; RPL6; RP	4736; 6124; 6203; 6137; 6227; 6161	118	117
5.92E-61	6.43E-60	Nonsense-Mediated Decay (NMD)	Reactome	R-HSA-927802	RPS13; RPS12; RPS11; RPS10; RPS17; RPS16; RPL27A; RPL9; RPL6; RP	4736; 6124; 6203; 6137; 6227; 6161	118	117
1.97E-59	2.00E-58	Selenoamino acid metabolism	Reactome	R-HSA-2408522	RPS13; KARS; RPS11; RPS10; RPS17; RPS16; RPL27A; RPS12; RPL6; RP	4736; 6124; 6203; 6137; 6227; 6161	130	129
1.35E-32	1.30E-31	Metabolism of RNA	Reactome	R-HSA-8953854	TRMT112; RPL26; PARN; RPL23; FAU; NUP214; RPL14; UTP14A; RPL1	6155; 10436; 10813; 6229; 63892; 6	586	584
3.88E-28	3.52E-27	Metabolism of amino acids and derivatives	Reactome	R-HSA-71291	RPS13; KARS; RPS11; RPL37A; RPS17; RPS16; RPL27A; RPL9; RPL6; RP	6155; 3735; 6191; 6229; 6133; 6144	342	339
3.75E-25	3.22E-24	Formation of the ternary complex, and subsequently, the 43S complex	Reactome	R-HSA-72695	RPS13; RPS12; RPS11; RPS10; RPS17; RPS16; RPS15; RPS19; RPS27A;	6228; 6223; 6203; 6232; 6194; 6227	61	59
3.75E-23	2.91E-22	Translation initiation complex formation	Reactome	R-HSA-72649	RPS13; RPS12; RPS11; RPS10; RPS17; RPS16; RPS15; RPS19; RPS27A;	6228; 6223; 6203; 6232; 6194; 6227	68	66
3.75E-23	2.91E-22	Ribosomal scanning and start codon recognition	Reactome	R-HSA-72702	RPS13; RPS12; RPS11; RPS10; RPS17; RPS16; RPS15; RPS19; RPS27A;	6228; 6223; 6203; 6232; 6194; 6227	68	66
6.81E-23	5.04E-22	Activation of the mRNA upon binding of the cap-binding complex and eIFs, and subsequent binding to 43S	Reactome	R-HSA-72662	RPS13; RPS12; RPS11; RPS10; RPS17; RPS16; RPS15; RPS19; RPS27A;	6228; 27335; 6223; 6203; 6232; 619	69	67
2.17E-20	1.54E-19	Metabolism of proteins	Reactome	R-HSA-392499	TRAPPC2L; TRMT112; RPL26; TUSC3; SUMO2; CPE; TRAPPC2B; RPL23	6155; 54344; 6133; 6144; 6130; 620	2008	1999
1.23E-14	7.68E-14	Mitochondrial translation termination	Reactome	R-HSA-5419276	MRPL27; MRPL21; MRPL20; MRPL22; MRPS15; MRPS14; CHCHD1; M	90480; 55052; 7818; 122704; 54460	89	88
1.23E-14	7.68E-14	Mitochondrial translation elongation	Reactome	R-HSA-5389840	MRPL27; MRPL21; MRPL20; MRPL22; MRPS15; MRPS14; CHCHD1; M	90480; 55052; 7818; 122704; 54460	89	88
1.23E-14	7.68E-14	Mitochondrial translation initiation	Reactome	R-HSA-5368286	MRPL27; MRPL21; MRPL20; MRPL22; MRPS15; MRPS14; CHCHD1; M	90480; 55052; 7818; 122704; 54460	89	88
7.99E-14	4.83E-13	Mitochondrial translation	Reactome	R-HSA-5368287	MRPL27; MRPL21; MRPL20; MRPL22; MRPS15; MRPS14; CHCHD1; M	90480; 55052; 7818; 122704; 54460	95	94
1.59E-13	9.25E-13	Oxidative phosphorylation - Homo sapiens (human)	KEGG	path:hsa00190	ATP6V1F; COX5B; UQCR10; UQCR11; NDUFB4; ATP6V1H; NDUFB1; U	514; 517; 521; 529; 539; 9296; 4707	133	133
5.55E-13	3.12E-12	Respiratory electron transport, ATP synthesis by chemiosmotic coupling, and heat production by uncoupling proteins.	Reactome	R-HSA-163200	COX5B; UQCR10; UQCR11; NDUFB4; NDUFB1; UQCRQ; UQCRH; COX	4725; 10632; 10975; 29796; 1327; 5	123	123
8.64E-12	4.69E-11	Metabolism	Reactome	R-HSA-1430728	TRMT112; HADHA; MAN2B2; RPL12; MAN2B1; NDUFB7; SUMO2; ND	6155; 160428; 6133; 6144; 3707; 61	1972	1960
2.39E-10	1.26E-09	Alzheimer disease - Homo sapiens (human)	KEGG	path:hsa05010	COX5B; UQCR10; UQCR11; NDUFB4; NDUFB1; UQCRQ; UQCRH; COX	514; 517; 539; 4695; 4696; 4697; 47	171	171
2.80E-10	1.43E-09	Thermogenesis - Homo sapiens (human)	KEGG	path:hsa04714	COX5B; NDUFB7; UQCR11; NDUFB4; MGLL; NDUFB1; UQCRQ; RPS6;	514; 51287; 517; 4701; 6194; 11343	229	229
7.01E-10	3.46E-09	Respiratory electron transport	Reactome	R-HSA-611105	COX5B; UQCR10; UQCR11; NDUFB4; NDUFB1; UQCRQ; UQCRH; COX	4725; 10975; 29796; 1327; 4696; 51	100	100
3.35E-09	1.60E-08	Parkinson disease - Homo sapiens (human)	KEGG	path:hsa05012	COX5B; NDUFB7; UQCR11; NDUFB4; NDUFB1; UQCRQ; UQCRH; COX	514; 517; 539; 11315; 4695; 4696; 4	142	142
5.52E-09	2.57E-08	The citric acid (TCA) cycle and respiratory electron transport	Reactome	R-HSA-1428517	COX5B; UQCR10; UQCR11; NDUFB4; NDUFB1; UQCRQ; UQCRH; COX	4725; 10632; 4707; 29796; 1327; 53	173	173
7.79E-08	3.53E-07	Huntington disease - Homo sapiens (human)	KEGG	path:hsa05016	COX5B; NDUFB7; UQCR11; NDUFB4; NDUFB1; UQCRQ; UQCRH; COX	514; 4696; 1340; 539; 517; 4695; 46	193	193
4.05E-06	1.78E-05	Proteasome - Homo sapiens (human)	KEGG	path:hsa03050	POMP; PSMD4; PSMA2; SEM1; PSMA4; PSMC1; PSMB7; PSMB6; PSM	51371; 7979; 5683; 5685; 5689; 569	45	45
7.33E-06	3.14E-05	Non-alcoholic fatty liver disease (NAFLD) - Homo sapiens (human)	KEGG	path:hsa04932	COX5B; NDUFB7; UQCR11; NDUFB4; NDUFB1; UQCRQ; UQCRH; COX	4695; 4696; 4697; 4701; 4707; 4710	149	149
6.77E-05	0.0002828	TP53 Regulates Metabolic Genes	Reactome	R-HSA-5628897	TP63; NDUFA4; MOV10; COX5B; TXN; PRDX1; COX4I1; COX6C; COX6	8626; 1350; 7295; 28956; 1327; 132	86	86
0.0001787	0.0007283	Complex I biogenesis	Reactome	R-HSA-6799198	NDUFA7; NDUFA2; NDUFA3; NDUFB7; NDUFB4; NDUFB1; NDUFS4; N	4707; 4725; 4696; 4695; 4724; 4710	55	55
0.0002491	0.0009904	Formation of TC-NER Pre-Incision Complex	Reactome	R-HSA-6781823	RPS27A; COPS6; GTF2H5; UBA52; POLR2F; RBX1; POLR2L; MNAT1; P	5435; 5439; 4331; 5441; 10450; 543	57	57
0.0002592	0.0010059	Rho GTPase cycle	Reactome	R-HSA-194840	ARHGEF10; PLEKHG2; TRIO; ARHGAP31; ARAP3; ARHGEF15; PLEKHG	23380; 85360; 392; 23263; 57449; 2	144	140
0.0002916	0.0011054	Signaling by NOTCH	Reactome	R-HSA-157118	NEURL1B; HEYL; MOV10; PLXND1; SKP1; RPS27A; NOTCH1; HIF1A; N	4854; 4242; 182; 4343; 2683; 11060	120	119
0.0003175	0.0011763	Gap-filling DNA repair synthesis and ligation in TC-NER	Reactome	R-HSA-6782210	RPS27A; GTF2H5; RFC2; UBA52; POLE4; POLR2F; RBX1; POLR2L; MN	56655; 5435; 5439; 4331; 5441; 104	68	68
0.0003254	0.0011786	Cardiac muscle contraction - Homo sapiens (human)	KEGG	path:hsa04260	SLC9A1; COX5B; UQCR10; UQCR11; ATP1B2; UQCRQ; COX4I1; UQCR	1350; 1327; 1329; 1340; 1346; 1345	78	78
0.0003654	0.0012947	Dual incision in TC-NER	Reactome	R-HSA-6782135	RPS27A; GTF2H5; RFC2; UBA52; POLE4; POLR2F; RBX1; POLR2L; MN	56655; 5435; 5439; 4331; 5441; 104	69	69
0.0003829	0.001328	Signaling by VEGF	Reactome	R-HSA-194138	NOS3; CYFIP1; CALM1; DOCK1; NRP1; BRK1; KDR; CTNND1; ITPR3; FL	3791; 4846; 8828; 23191; 1500; 984	100	100
0.0004754	0.0016144	Transcription-Coupled Nucleotide Excision Repair (TC-NER)	Reactome	R-HSA-6781827	RPS27A; COPS6; GTF2H5; RFC2; UBA52; POLE4; POLR2F; RBX1; POLR	56655; 5435; 5439; 4331; 5441; 104	81	81
0.0004871	0.0016204	mRNA Splicing - Minor Pathway	Reactome	R-HSA-72165	SNRPD2; SNRPD1; SF3B5; POLR2F; ZMAT5; SNU13; POLR2L; POLR2J;	5441; 5435; 55954; 6635; 83443; 54	52	52
0.000739	0.0024091	Axon guidance	Reactome	R-HSA-422475	EFNB2; SCN7A; EFNB1; COL4A1; ITGA1; RPS27A; GAB2; RET; SRGAP2	1605; 1282; 3672; 116986; 219699;	358	357
0.000902	0.0028829	Cell death signalling via NRAGE, NRIF and NADE	Reactome	R-HSA-204998	PLEKHG2; ARHGEF10; TRIO; RPS27A; FGD1; ARHGEF15; PREX1; PLEK	7204; 57580; 7531; 2245; 64857; 73	78	76
0.0011864	0.0037189	HIV Infection	Reactome	R-HSA-162906	RPS27A; ATP6V1H; TAF12; CHMP2A; ELOB; XRCC6; SKP1; NPM1; VPS	4331; 8815; 2547; 9525; 4869; 2332	197	195
0.00125	0.0038445	Neddylation	Reactome	R-HSA-8951664	PSMD4; RPS27A; COPS6; HIF1A; ELOB; NEDD8; COMMD9; SKP1; CON	5691; 5702; 29099; 5694; 6233; 569	235	234
0.0013825	0.0041732	Retrograde endocannabinoid signaling - Homo sapiens (human)	KEGG	path:hsa04723	DAGLA; NDUFA7; NDUFA4; ADCY7; NDUFA2; NDUFA3; NDUFB7; ADC	4695; 54539; 11343; 4696; 4697; 47	148	148
0.001448	0.0042915	UCH proteinases	Reactome	R-HSA-5689603	NEDD8; PSMD4; RPS27A; PSMA2; UBA52; SEM1; PSMA4; PSMC1; PS	5691; 5702; 5694; 5693; 5689; 5700	102	102
0.0016136	0.0046968	Death Receptor Signalling	Reactome	R-HSA-73887	ARHGEF10; PLEKHG2; TRIO; TNFSF10; FGD1; ARHGEF15; YWHAE; PR	7204; 23263; 8743; 22899; 9639; 90	141	138
0.0018577	0.0053125	Extracellular matrix organization	Reactome	R-HSA-1474244	COLGALT1; COL11A2; ITGA1; HSPG2; VCAN; ITGA6; MFAP1; LAMB1;	3791; 1306; 1605; 7837; 4811; 3911	294	294
0.0021488	0.0060389	Signaling by NOTCH1	Reactome	R-HSA-1980143	NEURL1B; HEYL; SKP1; RPS27A; NOTCH1; HIF1A; UBA52; DLL4; RBX1;	54492; 182; 26508; 4851; 7311; 545	74	73
0.0022398	0.006188	Signaling by Receptor Tyrosine Kinases	Reactome	R-HSA-9006934	PDGFRB; ATP6V1F; CYFIP1; RPS27A; PCSK6; ATP6V1H; SPRED2; BRK1	200734; 5439; 9365; 8829; 2324; 50	423	423
0.0030076	0.0081708	Spliceosome - Homo sapiens (human)	KEGG	path:hsa03040	LSM7; SNRPD2; SNRPD1; SF3B5; PRPF18; CTNNBL1; LSM3; SNRPC; Z	27258; 4809; 10450; 10465; 51503;	134	134
0.0032632	0.0087197	Infectious disease	Reactome	R-HSA-5663205	ANTXR1; RPS27A; ATP6V1H; TAF12; CHMP2A; ELOB; CTNND1; XRCC	4331; 8815; 2547; 5435; 5439; 801;	253	251
0.0038179	0.0100375	Nucleotide Excision Repair	Reactome	R-HSA-5696398	RPS27A; POLE4; SUMO2; COPS6; GTF2H5; RFC2; UBA52; POLR2F; PO	10450; 5982; 5439; 4331; 404672; 6	113	113
0.0047118	0.0121909	Collagen formation	Reactome	R-HSA-1474290	COL15A1; LOXL2; COLGALT1; COL11A2; PLOD2; PLOD1; ITGA6; COL5	1306; 4017; 7837; 1302; 79709; 535	92	92
0.0049208	0.0125328	Transcriptional Regulation by TP53	Reactome	R-HSA-3700989	MOV10; COX5B; RPS27A; PRDX1; RFC2; CASP10; TAF12; TNFRSF10A;	1107; 5439; 8797; 1350; 7531; 8626	374	371
0.0051202	0.0128398	HIV Life Cycle	Reactome	R-HSA-162587	NEDD4L; VPS28; RPS27A; TAF12; GTF2H5; BANF1; UBA52; CHMP2A;	4331; 8815; 2547; 9525; 51160; 543	156	154
0.0053933	0.0133199	ECM-receptor interaction - Homo sapiens (human)	KEGG	path:hsa04512	LAMA5; FN1; ITGA1; THBS4; HSPG2; ITGA6; DAG1; COL4A1; LAMB1;	1282; 1284; 1293; 7060; 1605; 2335	82	82
0.0058162	0.01415	Muscle contraction	Reactome	R-HSA-397014	FXYD6; TMOD4; TNNT3; RYR3; CLIC2; TMOD3; SCN7A; ITGA1; KCNK6;	29766; 1193; 859; 29765; 6332; 538	195	195
0.0066383	0.0156817	PTEN Regulation	Reactome	R-HSA-6807070	CHD3; RPS27A; PREX2; MECOM; WWP2; LAMTOR4; UBA52; PHC1; S	1107; 6591; 1911; 28956; 84733; 80	96	96
0.0066383	0.0156817	p75 NTR receptor-mediated signalling	Reactome	R-HSA-193704	ARHGEF10; PLEKHG2; TRIO; RPS27A; FGD1; ARHGEF15; PREX1; PLEK	23263; 22899; 9639; 64857; 26084;	99	96
0.0073788	0.0171822	mRNA Splicing	Reactome	R-HSA-72172	PPIH; DNAJC8; SNRPD2; SNRPD1; SF3B5; POLR2L; LSM7; LSM3; SNRP	10450; 83443; 56259; 51503; 6632;	186	186
0.0084107	0.0193091	G alpha (12/13) signalling events	Reactome	R-HSA-416482	ARHGEF10; PLEKHG2; TRIO; FGD1; ARHGEF15; PREX1; GNB4; PLEKH	23263; 22899; 9639; 64857; 26084;	89	87
0.0088784	0.0200998	Apelin signaling pathway - Homo sapiens (human)	KEGG	path:hsa04371	ADCY4; NOTCH3; ADCY7; APLNR; SLC9A1; GABARAPL2; GNB4; RYR3;	6194; 11337; 11345; 113; 6263; 371	137	137
0.0099009	0.0221075	mRNA Splicing - Major Pathway	Reactome	R-HSA-72163	PPIH; DNAJC8; SNRPD2; SNRPD1; SF3B5; POLR2L; LSM7; LSM3; SNRP	10450; 83443; 56259; 51503; 6632;	178	178

Supplementary Table 5. Gene set enrichment analysis (GSEA) between CKD and control.

Gene Sets	Number of Genes	Direction	P-Value	Adjusted P-value
HALLMARK_OXIDATIVE_PHOSPHORYLATION	200	Up	7.50E-16	3.75E-14
HALLMARK_MYC_TARGETS_V1	193	Up	4.43E-12	1.11E-10
HALLMARK_DNA_REPAIR	146	Up	0.000489754	0.002320234
HALLMARK_MYC_TARGETS_V2	57	Up	0.00177854	0.006204822
HALLMARK_E2F_TARGETS	167	Up	0.004517185	0.01188733
HALLMARK_REACTIVE_OXIGEN_SPECIES_PATHWAY	48	Up	0.019567373	0.039134745
HALLMARK_FATTY_ACID_METABOLISM	141	Up	0.022430812	0.043136177
HALLMARK_EPITHELIAL_MESENCHYMAL_TRANSITION	174	Down	2.61E-06	4.35E-05
HALLMARK_ANGIOGENESIS	28	Down	4.76E-06	5.95E-05
HALLMARK_APICAL_JUNCTION	169	Down	4.20E-05	0.000420087
HALLMARK_WNT_BETA_CATENIN_SIGNALING	37	Down	7.96E-05	0.00066292
HALLMARK_INTERFERON_ALPHA_RESPONSE	89	Down	0.000243326	0.00156728
HALLMARK_TGF_BETA_SIGNALING	51	Down	0.000250765	0.00156728
HALLMARK_INTERFERON_GAMMA_RESPONSE	179	Down	0.000409665	0.002275915
HALLMARK_UV_RESPONSE_DN	137	Down	0.000510451	0.002320234
HALLMARK_HEDGEHOG_SIGNALING	29	Down	0.00058082	0.002420085
HALLMARK_IL2_STAT5_SIGNALING	163	Down	0.001351235	0.005197059
HALLMARK_COAGULATION	100	Down	0.001861447	0.006204822
HALLMARK_IL6_JAK_STAT3_SIGNALING	64	Down	0.003136824	0.009265315
HALLMARK_NOTCH_SIGNALING	31	Down	0.003150207	0.009265315
HALLMARK_COMPLEMENT	162	Down	0.003647576	0.010132154
HALLMARK_KRAS_SIGNALING_UP	156	Down	0.005043272	0.01260818
HALLMARK_APICAL_SURFACE	32	Down	0.006503832	0.015485315
HALLMARK_ESTROGEN_RESPONSE_EARLY	163	Down	0.010805356	0.024557627
HALLMARK_MITOTIC_SPINDLE	176	Down	0.013739249	0.028885432
HALLMARK_INFLAMMATORY_RESPONSE	142	Down	0.013865007	0.028885432

Supplementary Table 6. Gene set enrichment analysis (GSEA) between Dialysis and CKD.

Gene Sets	Number of Genes	Direction	P-Value	Adjusted P-value
HALLMARK_INTERFERON_ALPHA_RESPONSE	89	Up	1.19E-07	5.97E-06
HALLMARK_INTERFERON_GAMMA_RESPONSE	179	Up	3.16E-05	0.00039557
HALLMARK_UV_RESPONSE_DN	137	Down	1.70E-06	4.25E-05
HALLMARK_ANDROGEN_RESPONSE	90	Down	1.30E-05	0.000216151
HALLMARK_PROTEIN_SECRETION	93	Down	0.000452543	0.00452543
HALLMARK_MTORC1_SIGNALING	194	Down	0.00343862	0.028655168
HALLMARK_ANGIOGENESIS	28	Down	0.006345449	0.042921575
HALLMARK_ADIPOGENESIS	195	Down	0.006867452	0.042921575
HALLMARK_BILE_ACID_METABOLISM	83	Down	0.009425769	0.049756731
HALLMARK_MYC_TARGETS_V1	193	Down	0.009951346	0.049756731

Supplementary Table 7. Laboratory Data

	Control	CKD	Dialysis*
Number	16	29	9
Hemoglobin (g/dL)	13.5 ± 1.5	11.3 ± 2.2	10.0 ± 1.2
Bicarbonate (mEq/L)	24.6 ± 2.3	22.0 ± 3.7	23.3 ± 2.8
Calcium (mg/dL)	….	9.5 ± 0.6	8.9 ± 0.5
Phosphate (mg/dL)	….	4.0 ± 0.9	5.6 ± 1.1
Parathyroid hormone (pg/mL)	….	150 (93, 264)	432.3 (246, 507)
Potassium (mEq/L)	….	4.7 ± 0.5	4.4 ± 0.6

Abbreviations: CKD, chronic kidney disease.

Data presented as mean ± standard deviation or median (interquartile range).

*Dialysis category includes 5 participants whose first muscle biopsy occurred after they had started dialysis and 4 participants from the CKD group who underwent a second muscle biopsy after starting dialysis. Calcium, phosphate, and parathyroid hormone values were not available for one participant in the Dialysis group.

Supplementary Table 8. Dietary variables before and after initiation of dialysis

Change in dietary variables after initiation of dialysis (n=4)			
	Pre-dialysis	Post-dialysis	P value
Protein (g/day)	82 (52-117)	65 (53-89)	0.27
% kcal from protein	14.0 (12.3-16.2)	16.7 (15.0-19.9)	0.07
Energy (kcal)	2318 (1689-2884)	1406 (1327-1955)	0.07

Data reported as median (interquartile range). P values calculated using the Wilcoxon matched-pairs signed-rank test.

Supplementary Appendix. Full Methods

Study Population

The population of this study is drawn from two studies which have been previously described (14, 109). All patients with CKD were recruited from the nephrology clinics and faculty practice at Montefiore Medical Center. Participants with CKD stages 4 and 5 and ESRD and healthy sedentary controls were recruited between March 2015 and January 2020 from a prospective cohort study of patients with an eGFR <30 $ml \cdot min^{-1} \cdot 1.73\ m^{-2}$ (14). Eligible patients were ≥21 years of age and able to provide written informed consent for study participation. Exclusion criteria included lower extremity amputation, use of immunosuppressive medication in the previous 3 months, and current cancer diagnosis and/or treatment, and additionally, for muscle biopsy, the use of anticoagulant medications or antiplatelet agents that could not be stopped for at least one week. Healthy, sedentary individuals without evidence of kidney disease (eGFR>60 $mL/min/1.73m^2$ and urine albumin:creatinine ratio <30 mg/g) were recruited as control participants. Sedentary was defined as not participating in physically strenuous work, brisk physical leisure activity, or formal exercise more than once per week in the previous 3 months. Study visits occurred in the Clinical Research Center every 3 months. Dietary protein and energy intake were assessed using the Dialysis FFQ (110), which is a modified version of the Block FFQ (111), a validated instrument that produces estimates of an individual's food intake based on food groups, portion size and frequency. The Dialysis FFQ was administered by a member of the study team. Participants were asked to estimate their food intake for the last 3 months and for seasonal foods to estimate intake at the time of availability. FFQs were analyzed by NutriQuest (Berkeley, CA) using the Minnesota Nutrition Data System for Research (112). In addition, urine urea nitrogen (UUN) measured in timed 24-hour urine collections was used to calculate DPI among CKD patients and controls as follows: DPI=6.25 x (UUN (g/d) + weight (kg) x 0.031) (113). Unilateral knee extensor strength was measured using isometric dynamometry with a handheld dynamometer (Manual Muscle Test System, Lafayette Instrument, Lafayette, IN). To ensure assessment of maximum strength, subjects were instructed to perform a maximal exertion contraction, and two trials were recorded. The highest result achieved in each leg was used for analysis. Endurance capacity was measured by the 2-minute walk test (114): Participants were asked to walk back and forth over a 50-foot course as far as possible over 2 minutes. The distance covered is highly correlated with 6-minute walk distance (115). The SPPB is an established measure of mobility in older adults (116). It includes a 4-meter walk test,

a standing balance test, and a 5-repetition sit-to-stand test, and is scored 0-12, with higher scores indicating better function. Handgrip strength was measured twice in each hand using a handheld dynamometer (North Coast Medical, Morgan Hill, CA). The maximum value attained using the dominant hand was used for analysis. Physical activity level was measured using triaxial accelerometers (Actigraph GT3X-BT, Actigraph, Pensacola, FL) worn around the waist for 7 consecutive days. Data processing was performed using 60-second epochs in ActiLife 6.13.3 and wear-time validation was performed as previously described (117). Intensity levels were defined based on counts-per-minute (cpm): sedentary, <100 cpm; light, 100-1951 cpm; moderate, 1952-5724 cpm; vigorous, 5725-9498 cpm; and very vigorous, ≥9499 cpm (118, 119). Sedentary time was classified according to daily time spent in sedentary bouts of 10 or more consecutive minutes, excluding sleep time (117). We previously reported ECM collagen content by picro-sirius red staining in a subset of these participants (10 CKD patients and 10 control participants). Additional participants with CKD stages 3 and 4 were enrolled in a multicenter, double-blind, randomized, placebo-controlled trial of sodium bicarbonate therapy (ClinicalTrials.gov identifier NCT01452412) and underwent muscle biopsy between September 2011 and May 2015 (109). Only participants enrolled at the Albert Einstein College of Medicine were eligible for the biopsy component of the study. Data reported here, including muscle biopsy results, were collected at baseline prior to initiating study treatment. Exclusion criteria included treatment with alkali therapy in the previous 3 months, serum bicarbonate levels < 20 or >26 mEq/L, New York Heart Association class III or IV heart failure, systolic blood pressure > 180 mm Hg, initiation of kidney replacement therapy planned within 6 months, and use of immunosuppressive medication in the previous 3 months. Medical history was collected by standardized questionnaire and medical record review. Lower extremity performance was assessed using a 10-repetition sit-to-stand test; the split time required to complete 5 repetitions was also recorded. Handgrip strength was measured as described above. Serum creatinine, serum bicarbonate, and hemoglobin were measured at study visits, and other laboratory parameters were measured as part of routine clinical care. All laboratory tests were conducted in the clinical laboratory of Montefiore Medical Center. eGFR was calculated by the Chronic Kidney Disease Epidemiology Collaboration (CKD-EPI) equation (120). Study data were collected and managed using REDCap (Research Electronic Data Capture) electronic data capture tools hosted at the Albert Einstein College of Medicine (121). Race/ethnicity was self-identified using categories consistent with NIH guidelines.

Muscle Biopsies and Tissue Processing

Biopsy procedures have been previously described (14). Subjects were admitted to the Clinical Research Center at 8:00am following an overnight fast. Through an incision site 15 cm proximal to the superior border of the patella, approximately 100-150 mg of muscle tissue was collected from vastus lateralis using a 12-gauge biopsy needle (Bard Monopty, Bard Biopsy Systems, Tempe, AZ). A portion of the muscle biopsy was immediately flash frozen in liquid nitrogen for RNA and biochemical analysis. A portion of the muscle sample selected based on optimal orientation of myofibers was gently separated for immunohistochemical preparation. The remaining portion of muscle tissue was quickly blotted to remove excess blood and immediately flash frozen in liquid nitrogen and stored at -80°C. For immunohistochemical analysis, muscle was covered in Tissue Tek (O.C.T. Compound, Sakura Finetek, Torrance, CA, USA) at resting length and frozen in liquid nitrogen-cooled 2-methylbutane, then stored at -80°C until analysis. Sample size for outcomes listed below was dependent on sufficient tissue quantity; certain participant biopsy samples provided insufficient muscle tissue for all downstream assays.

Immunohistochemistry

Seven μm-thick sections were cut with a cryostat at -25°C (HM525-NX, Thermo Fisher Scientific, Waltham, MA, USA) and air dried on slides for 1 h. Slides were then stored at -20°C until immunohistochemical/histochemical staining was performed. Immunohistochemical techniques were performed as previously described by our group (122).

Picro-sirius red staining to denote ECM collagen content was performed as previously described (28). Sections were fixed in Bouin's solution (#15990-10, Electron Microscopy Sciences) in a water bath at 56°C for 1 h. Following a brief wash, sections were incubated in Sirius Red solution (ab150681, Abcam; 0.1% in saturated picric acid) for 2 h at room temperature. Slides were then washed in 0.5% acetic acid, dehydrated in 95% and 100% ethanol, equilibrated in xylenes, and mounted in xylene-based mounting media.

For immunofluorescent detection of satellite cells and capillaries, sections were fixed in acetone at -20°C for 3 min, washed in phosphate-buffered saline (PBS; pH 7.5), and placed in 3% hydrogen peroxide for 7 min to block endogenous peroxidases. Slides were then washed in PBS and blocked for 1 h at room temperature in 2.5% normal horse serum (NHS; #S-2012,

Vector Laboratories), followed by overnight incubation at 4°C in the following primary antibodies diluted in 2.5% NHS: anti-myosin heavy chain type I mouse IgG2b at 1:75 (BA.D5-C, Developmental Studies Hybridoma Bank (DHSB), University of Iowa), anti-laminin rabbit IgG at 1:200 (#L9393, Sigma), anti-Pax7 mouse IgG at 1:100 (concentrate from DHSB), and rhodamine labeled Ulex Europaeus Agglutinin I at 1:50 to detect capillaries (#RL-1062, Vector Laboratories) (34, 123). The Pax7 antibody was deposited to the DSHB by Kawakami, A. (DSHB Hybridoma Product PAX7), and the BA-D5 antibody was deposited to the DSHB by Schiaffino, S. (DSHB Hybridoma Product BA-D5). The next morning, slides were washed in PBS and incubated for 1 h at room temperature in the following secondary antibodies diluted in 2.5% NHS: goat anti-mouse IgG2b AF647 at 1:500 to detect myosin heavy chain type I (#A21242, Invitrogen), goat anti-rabbit IgG AF647 at 1:500 to detect laminin (#A21245, Invitrogen), and biotinylated goat anti-mouse IgG at 1:1000 (#115-065-205, Jackson Immuno Research). Slides were washed and incubated for 1 h at room temperature in streptavidin-horse radish peroxidase included in a commercially available TSA kit (SA-HRP, Life Technologies/Thermo Fisher Scientific, Waltham, MA USA). Following a PBS wash, slides were incubated in TSA-Alexa Fluor 488 to amplify Pax7. Lastly, sections were incubated for 10 min in 4′,6-diamidino-2-phenylindole (DAPI; 10 nM, LifeTechnologies/Thermo Fisher Scientific), washed in PBS, and mounted with Vectashield fluorescence mounting media (Vector Laboratories, Burlingame, CA, USA).

For identification of ki67+ satellite cells, sections were fixed for 7 min at room temperature in 4% paraformaldehyde (PFA) followed by epitope retrieval in sodium citrate (10mM, pH 6.5) at 92°C. Endogenous peroxidases were blocked by placing slides in 3% hydrogen peroxide for 7 min, followed by a 1 h block at room temperature in 1% blocking reagent included in a commercially available tyramide signal amplification kit (TSA, Life Technologies/Thermo Fisher Scientific). Slides were then incubated overnight at 4°C in rabbit primary antibody against ki67 (#CRM325B, Biocare Medical) at 1:100 in 1% TSA blocking reagent and mouse primary antibody against Pax7 (concentrate from DHSB) at 1:100 in 1% TSA blocking reagent. The following day, slides were washed in PBS and incubated for 80 min at room temperature in goat anti-rabbit IgG secondary antibody conjugated to AF555 (#A21249, Invitrogen) at 1:250 in PBS and biotinylated goat anti-mouse IgG secondary antibody ((#115-065-205, Jackson Immuno Research) at 1:250 in PBS. Slides were washed and incubated for 1 h at room temperature in SA-HRP, followed by amplification of Pax7 using TSA-Alexa Fluor 488 (Life Technologies/Thermo Fisher Scientific). Slides were then washed in PBS, incubated in DAPI

(10 nM, LifeTechnologies/Thermo Fisher Scientific) and mounted with Vectashield fluorescence mounting media (Vector Laboratories).

Image Acquisition and Analysis

Images were captured at x100-400 magnification at room temperature using a Zeiss upright microscope (AxioImager M1; Zeiss, Oberkochen, Germany). Image analysis was performed in a blinded manner using Image J Fiji or Zen software (v3.1, Zeiss). Picro-sirius red staining was quantified to measure collagen content of the ECM using Image J Fiji software as previously described (122). The area of pico-sirius red+ collagen was normalized to the total muscle area (mm^2). Picro-sirius red was also imaged under polarized light to quantify densely packed (red) and loosely packed (green) collagen relative to total muscle area (124, 125).

Satellite cell abundance was determined by co-staining of Pax7 and DAPI within the laminin border. Pax7+/DAPI+ cells within the laminin border were counted as satellite cells and normalized to total number of myofibers. Proliferation of satellite cells was assessed by costaining of ki67 and Pax7. Ki67+/Pax7+/DAPI+ cells inside the laminin border were counted as proliferating satellite cells and normalized to total satellite cell number. Capillaries were measured as *Ulex Europaeus* agglutinin-positive cellular structures outside the myofiber laminin border, as previously described (126). CFPE was used to assess capillary density relative to myofiber perimeter and was quantified as the ratio between the number of capillaries of each myofiber with a correction for capillary sharing and myofiber perimeter, as previously described (35). CFPE was calculated using at least 50 myofibers for each sample per published recommendations (127). Myovision software generated automated analysis of myofiber CSA, myonuclear density (total number of myonuclei normalized to total number of myofibers), and myonuclear domain (area of each individual myofiber normalized to number of myonuclei within that same myofiber) using laminin and DAP**I (128)**.

Hydroxyproline Biochemical Assay

To assess total muscle collagen content, hydroxyproline was assayed from approximately 10 mg of muscle tissue similar to our prior methods (129) using a modified protocol with a commercially available Hydroxyproline Assay Kit (MAK008, Millipore Sigma, Darmstadt, Germany). Following the vastus lateralis muscle biopsy, muscle samples were quickly flash

frozen in liquid nitrogen and stored at -80°C until analysis. The muscle was homogenized in double-distilled water (volume equal to 10x pellet weight: i.e. 100 μl double-distilled water for 10 mg pellet weight). When adequately homogenized, the sample was vortexed thoroughly and 12M HCl (volume equal to double-distilled water used for homogenization) was added to hydrolyze the sample overnight at 105°C. Following hydrolysis, the sample/hydrolysate was vortexed and 20 μl was loaded in duplicate to a 96-well plate with hydroxyproline standard (MAK008, Millipore Sigma). The plate was dried overnight at 60°C. Chloramine T/Oxidation Buffer included in the Hydroxyproline Assay Kit was added to each plate well and incubated at room temperature for 5 min. Next, diluted p-dimethylaminobenzaldehyde (DMAB) Reagent (MAK008, Millipore Sigma) was pipetted into each well followed by incubation for 90 min at 60°C. Absorbance was measured on a microplate reader at 595nm, and hydroxyproline content was calculated by a standard curve then normalized to the loaded sample volume (20 μl).

RNA-Sequencing

RNA isolation

Muscle sample was homogenized in Tri-reagent using Zirconium Oxide beads (2.0 mm, RNase-free, Next Advance, Inc. Troy, NY) homogenization at 4°C. The homogenate was centrifuged at 15,000 g, 4°C for 15 min. The supernatant was used for muscle total RNA extraction using Direct-zolTM RNA MiniPrep Plus (Zymo Research, Irvine, CA) with DNase I treated on column (14).

Sequencing, preprocessing and alignment

Total RNA was sequenced on Illumina HiSeq 4000 system at Novogene Corporation, Chula Vista, CA, using a paired-end 150 bp dual-indexing protocol. Raw fastq reads were passed through quality control using FastQC (v0.11.4) and the results were compiled using MultiQC (v1.7) (130, 131). Reads that were detected for adapter contamination were subjected to adapter trimming, while low quality and too short reads were filtered using default parameters in fastp (v0.19.4)- an all-in-one Fastq preprocessing tool (132). The reads that passed quality control were aligned to GRCh38 primary build of the reference human genome with transcript annotations (gencode.v29.annotation.gtf) obtained from GENCODE, using the STAR aligner (v2.6.1b) (133, 134). The transcripts were quantified using RNA-Sequencing by Expectation Maximization- RSEM (v1.3.0) (135). The raw sequences and the quantified data are available in

the Gene Expression Omnibus (GEO) database under the accession code GSE157712 (publicly available 03/2021).

Statistical Modeling, Differential Gene Expression and GSEA

All statistical tests for RNA-Seq analysis were carried out using the R statistical software R 4.0.2.

The raw counts were filtered using a counts-per-million cutoff of 0.5 (10/minimum library size in millions), in at least 7 samples (number of samples in the smallest group of comparison). The raw counts were normalized using the trimmed mean of M-values (TMM) normalization in limma (v3.42.2). The voom function was applied to the normalized data to minimize heteroskedasticity and include precision weights for the mean-variance relationship for all genes (136). A principal component analysis was run to visually inspect any evident batch effects within the data, following which a linear model was fit on the voom-normalized data, while using sample ID as the blocking variable to account for subject-specific variability between the paired CKD and dialysis samples. Differential gene expression was calculated for the following contrasts using the empirical Bayes statistic in limma- 1) CKD patients vs healthy controls; 2) CKD patients pre vs post dialysis. The raw p-values were adjusted for multiple testing using the Benjamini-Hochberg correction and a threshold of q-value < 0.05 was used to categorize the genes as differentially expressed. Pathway overrepresentation within the differentially expressed genes was carried out using ConsensusPathdb (137). Gene Set Enrichment Analysis was carried out on the voom-normalized gene expression data using the CAMERA algorithm within limma (138). The Hallmark gene sets for humans curated by the Walter and Eliza Hall Institute were used for GSEA and downloaded from http://bioinf.wehi.edu.au/software/MSigDB/human_H_v5p2.rdata.

Statistics

Histochemistry, immunohistochemistry and hydroxyproline data were compared between CKD patients and controls using two-tailed t-tests or Wilcoxon rank-sum tests. Spearman correlation coefficients and linear regression models were used to test associations of eGFR with muscle outcome measures. Analyses including data collected from ESRD patients receiving dialysis were performed using mixed effects models including random intercepts to account for repeated measures in the subset who had undergone serial biopsies before and after the initiation of dialysis or who had second biopsies while non-dialysis dependent. Subjects noted as CKD

Stage 4/5 in Figure 4 represent a subset of all CKD subjects, specifically those with an eGFR <30 mL/min/1.73m^2. Satellite cell abundance, satellite cell-capillary distance, densely packed collagen content, and loosely packed collagen content were log-transformed to satisfy model assumptions. Multivariable linear regression and mixed effects models were adjusted for age, sex, race, and history of diabetes, hypertension, and cardiovascular disease unless otherwise noted. To calculate the CKD severity score, we computed the sum of standardized differences from the mean for serum bicarbonate, potassium, calcium, phosphate, parathyroid hormone, and hemoglobin. For serum bicarbonate, serum calcium, and hemoglobin, we input the negative of the standardized value into the summative score, as progression of non-dialysis dependent CKD induces a decrease in each parameter. All analyses were performed with Stata 13.1 (StataCorp, College Station, TX). A p-value <0.05 was considered statistically significant.

Study Approval

The study protocols were approved by the Institutional Review Board of the Albert Einstein College of Medicine. Before inclusion in the study, written informed consent was provided by all participants.
